# Rare *SLC13A1* variants associate with intervertebral disc disorder highlighting role of sulfate in disc pathology

**DOI:** 10.1038/s41467-022-28167-1

**Published:** 2022-02-02

**Authors:** Gyda Bjornsdottir, Lilja Stefansdottir, Gudmar Thorleifsson, Patrick Sulem, Kristjan Norland, Egil Ferkingstad, Asmundur Oddsson, Florian Zink, Sigrun H. Lund, Muhammad S. Nawaz, G. Bragi Walters, Astros Th. Skuladottir, Sigurjon A. Gudjonsson, Gudmundur Einarsson, Gisli H. Halldorsson, Valgerdur Bjarnadottir, Gardar Sveinbjornsson, Anna Helgadottir, Unnur Styrkarsdottir, Larus J. Gudmundsson, Ole B. Pedersen, Thomas Folkmann Hansen, Thomas Werge, Karina Banasik, Anders Troelsen, Soren T. Skou, Lise Wegner Thørner, Christian Erikstrup, Kaspar Rene Nielsen, Susan Mikkelsen, DBDS Genetic Consortium, Steffen Andersen, Søren Brunak, Kristoffer Burgdorf, Henrik Hjalgrim, Gregor Jemec, Poul Jennum, Per Ingemar Johansson, Kasper Rene Nielsen, Mette Nyegaard, Mie Topholm Bruun, Ole Birger Pedersen, Khoa Manh Dinh, Erik Sørensen, Sisse Ostrowski, Pär Ingemar Johansson, Daniel Gudbjartsson, Hreinn Stefánsson, Unnur Þorsteinsdóttir, Margit Anita Hørup Larsen, Maria Didriksen, Susanne Sækmose, GO Consortium, Eleftheria Zeggini, Konstantinos Hatzikotoulas, Lorraine Southam, Arthur Gilly, Andrei Barysenka, Joyce B. J. van Meurs, Cindy G. Boer, André G. Uitterlinden, Unnur Styrkársdóttir, Lilja Stefánsdóttir, Helgi Jonsson, Thorvaldur Ingvarsson, Tõnu Esko, Reedik Mägi, Maris Teder-Laving, Shiro Ikegawa, Chikashi Terao, Hiroshi Takuwa, Ingrid Meulenbelt, Rodrigo Coutinho de Almeida, Margreet Kloppenburg, Margo Tuerlings, P. Eline Slagboom, Rob R. G. H. H. Nelissen, Ana M. Valdes, Massimo Mangino, Aspasia Tsezou, Eleni Zengini, George Alexiadis, George C. Babis, Kathryn S. E. Cheah, Tian T. Wu, Dino Samartzis, Jason Pui Yin Cheung, Pak Chung Sham, Peter Kraft, Jae Hee Kang, Kristian Hveem, John-Anker Zwart, Almut Luetge, Anne Heidi Skogholt, Marianne B. Johnsen, Laurent F. Thomas, Bendik Winsvold, Maiken E. Gabrielsen, Ming Ta Michael Lee, Yanfei Zhang, Steven A. Lietman, Manu Shivakumar, George Davey Smith, Jonathan H. Tobias, April Hartley, Tom R. Gaunt, Jie Zheng, J. Mark Wilkinson, Julia Steinberg, Andrew P. Morris, Ingileif Jonsdottir, Aron Bjornsson, Ingvar H. Olafsson, Elfar Ulfarsson, Josep Blondal, Arnor Vikingsson, Soren Brunak, Sisse R. Ostrowski, Henrik Ullum, Unnur Thorsteinsdottir, Hreinn Stefansson, Daniel F. Gudbjartsson, Thorgeir E. Thorgeirsson, Kari Stefansson

**Affiliations:** 1 deCODE Genetics/Amgen, Inc., Reykjavik, Iceland; 2 Faculty of Medicine, School of Health Sciences, University of Iceland, Reykjavik, Iceland; 3 School of Engineering and Natural Sciences, University of Iceland, Reykjavik, Iceland; 4 Landspitali University Hospital, Reykjavik, Iceland; 5 Department of Clinical Immunology, Zealand University Hospital, Køge, Denmark; 6 Department of Clinical Medicine, Faculty of Health and Medical Sciences, University of Copenhagen, Copenhagen, Denmark; 7 Danish Headache Center, Dept. Neurology, Rigshospitalet-Glostrup, Glostrup, Denmark; 8 Novo Nordisk Foundation Center for Protein Research, Faculty of Health and Medical Sciences, University of Copenhagen, Copenhagen, Denmark; 9 Institute of Biological Psychiatry, Mental Health Services, Copenhagen University Hospital, Copenhagen, Denmark; 10 Lundbeck Foundation for GeoGenetics, GLOBE Institute, University of Copenhagen, Copenhagen, Denmark; 11 Department of Orthopaedic Surgery, CAG ROAD—Research OsteoArthritis Denmark, Copenhagen University Hospital, Hvidovre, Denmark; 12 Research Unit for Musculoskeletal Function and Physiotherapy, Department of Sports Science and Clinical Biomechanics, University of Southern Denmark, Odense, Denmark; 13 The Research Unit PROgrez, Department of Physiotherapy and Occupational Therapy, Næstved-Slagelse-Ringsted Hospitals, Næstved, Denmark; 14 Department of Clinical Immunology, Copenhagen University Hospital, Copenhagen, Denmark; 15 Department of Clinical Immunology, Aarhus University Hospital, Aarhus, Denmark; 16 Department of Clinical Immunology, Aalborg University Hospital, Aalborg, Denmark; 17 Department of Neurosurgery, Landspitali University Hospital, Reykjavik, Iceland; 18 Health Care Institution of West Iceland, Stykkisholmur, Iceland; 19 Statens Serum Institut, Copenhagen, Copenhagen, Denmark; 20 Department of Finance, Copenhagen Business School, Copenhagen, Denmark; 21 Department of Clinical Neurophysiology, University of Copenhagen, Copenhagen, Denmark; 22 Department of Biomedicine, Aarhus University, Aarhus, Denmark; 23 Department of Clinical Immunology, Odense University Hospital, Odense, Denmark; 24 Institute of Translational Genomics, Helmholtz Zentrum München, German Research Center for Environmental Health, Neuherberg, Germany; 25 Department of Internal Medicine, Erasmus MC, Medical Center, Rotterdam, The Netherlands; 26 Department of Orthopedic Surgery, Akureyri Hospital, Akureyri, Iceland; 27 Estonian Genome Center, Institute of Genomics, University of Tartu, Tartu, Estonia; 28 Laboratory for Bone and Joint Diseases, RIKEN Center for Integrative Medical Sciences, Tokyo, Japan; 29 Laboratory for Statistical and Translational Genetics, RIKEN Center for Integrative Medical Sciences, Kanagawa, Japan; 30 Department of Biomedical Data Sciences, Section Molecular Epidemiology, Leiden University Medical Center, Leiden, The Netherlands; 31 Departments of Rheumatology and Clinical Epidemiology, Leiden University Medical Center, Leiden, The Netherlands; 32 Department of Orthopaedics, Leiden University Medical Center, Leiden, The Netherlands; 33 Faculty of Medicine and Health Sciences, School of Medicine, University of Nottingham, Nottingham, Nottinghamshire UK; 34 Department of Twin Research and Genetic Epidemiology, Kings College London, London, UK; 35 Laboratory of Cytogenetics and Molecular Genetics, Faculty of Medicine, University of Thessaly, Larissa, Greece; 36 4th Psychiatric Department, Dromokaiteio Psychiatric Hospital, Haidari, Athens, Greece; 37 1st Department of Orthopaedics, KAT General Hospital, Athens, Greece; 38 2nd Department of Orthopaedics, National and Kapodistrian University of Athens, Medical School, Nea Ionia General Hospital ‘Konstantopouleio’, Athens, Greece; 39 School of Biomedical Sciences, The University of Hong Kong, Pokfulam, Hong Kong, China; 40 Department of Psychiatry, Li Ka Shing Faculty of Medicine, The University of Hong Kong, Pokfulam, Hong Kong, China; 41 Department of Orthopaedics and Traumatology, The University of Hong Kong, Pokfulam, Hong Kong, China; 42 Li Ka Shing Faculty of Medicine, The University of Hong Kong, Pokfulam, Hong Kong, China; 43 Department of Epidemiology, Harvard T.H. Chan School of Public Health, Boston, MA USA; 44 Department of Medicine, Brigham and Women’s Hospital, Boston, MA USA; 45 K. G. Jebsen Center for Genetic Epidemiology, Department of Public Health and Nursing, Faculty of Medicine and Health Sciences, Norwegian University of Science and Technology, Trondheim, Norway; 46 Department of Clinical and Molecular Medicine, Norwegian University of Science and Technology, Trondheim, Norway; 47 Genomic Medicine Institute, Geisinger Health System, Danville, PA USA; 48 Musculoskeletal Institute, Geisinger Health System, Danville, PA USA; 49 Department of Biostatistics, Epidemiology and Informatics, Perelman School of Medicine, University of Pennsylvania, Philadelphia, PA USA; 50 MRC Integrative Epidemiology Unit (IEU), Bristol Medical School, University of Bristol, Oakfield House, Oakfield Grove, Bristol, UK; 51 Musculoskeletal Research Unit, Translation Health Sciences, Bristol Medical School, University of Bristol, Southmead Hospital, Bristol, UK; 52 Department of Oncology and Metabolism and Healthy Lifespan Institute, University of Sheffield, Sheffield, UK; 53 Centre for Genetics and Genomics Versus Arthritis, Centre for Musculoskeletal Research, University of Manchester, Manchester, UK

**Keywords:** Genome-wide association studies, Pain, Bone

## Abstract

Back pain is a common and debilitating disorder with largely unknown underlying biology. Here we report a genome-wide association study of back pain using diagnoses assigned in clinical practice; dorsalgia (119,100 cases, 909,847 controls) and intervertebral disc disorder (IDD) (58,854 cases, 922,958 controls). We identify 41 variants at 33 loci. The most significant association (OR_IDD_ = 0.92, *P* = 1.6 × 10^−39^; OR_dorsalgia_ = 0.92, *P* = 7.2 × 10^−15^) is with a 3’UTR variant (rs1871452-T) in *CHST3*, encoding a sulfotransferase enzyme expressed in intervertebral discs. The largest effects on IDD are conferred by rare (MAF = 0.07 − 0.32%) loss-of-function (LoF) variants in *SLC13A1*, encoding a sodium-sulfate co-transporter (LoF burden OR = 1.44, *P* = 3.1 × 10^−11^); variants that also associate with reduced serum sulfate. Genes implicated by this study are involved in cartilage and bone biology, as well as neurological and inflammatory processes.

## Abstract (web summary)

Little is known about the biology of back pain, a leading cause of disability. Here the authors report 30 new back pain loci, implicating genes involved in cartilage/bone biology, as well as neurological and inflammatory processes.

## Introduction

Back pain is among the leading causes of years lived with disability worldwide^1^. One-month prevalence is around 20% and it affects up to 40% of people over 40 years of age^1–3^. Repeated debilitating episodes are common, with estimates of one-year recurrence ranging from 24 to 80%^4^. Commonly reported risk factors for back pain are age, lack of exercise, being overweight, smoking, tall stature, back-exertion, stress, anxiety, and depression^5,6^.

There is no single therapy proven effective for the majority of back pain sufferers^7^. Targeted treatments exist for some known causes, e.g., surgical interventions for back pain due to herniated intervertebral discs, vertebral fractures or cancers, but these account for <1% of back pain cases and surgery is not always better than non-surgical treatments in the long-term^5,6,8^. Other known back pain pathologies include intervertebral disc disorders (IDD), muscle spasms, osteoarthritis, and spinal stenosis^6^. Although IDD is a major contributor to back pain, clinical studies show that up to a third of 20-year-old individuals, and over 40% of 80 year olds without back pain have signs of severe IDD on imaging, and that in these groups the prevalence of disk degeneration is 39% and 96%, respectively^9^. Furthermore, signs of IDD detected by imaging do not predict back pain progression, severity or duration^10,11^.

To date, genome-wide association studies (GWAS) have yielded three loci harboring variants associating with self-reported back pain^12,13^ and severe lumbar IDD requiring surgery^14^. These are represented by variants in or near *CHST3*/*SPOCK2* and *SOX5*^12,13^, genes that are involved in regulation of chondrogenesis and the nervous system^15,16^, and an intergenic signal between *GSDMC* and *CCDC26* that associates with both self-reported back pain^12,13^ and lumbar IDD requiring surgery^14^.

Here, we report results of the largest genetic study of back pain phenotypes to date; meta-analyses of GWASs from Iceland (deCODE Genetics), Denmark (Danish Blood Donor Study; DBDS and Copenhagen Hospital Biobank; CHB), and the United Kingdom (UK Biobank; UKB), combined with summary statistics from Finland (FinnGen). We focus on two of the most common physician-assigned back pain diagnoses as defined under the International Statistical Classification of Diseases (ICD-10)^17^ that are IDD (code M51) and dorsalgia (code M54); representing largely known (IDD) and unknown (dorsalgia) etiologies of back pain. In total, we report 41 variants at 33 loci of which new associations with back pain are at 30 loci.

## Results

We meta-analyzed GWAS results (in total total 53.5 million sequence variants) of two back pain diagnoses from four countries; dorsalgia (119,100 cases, 909,847 controls) and intervertebral disc disorder (IDD) (58,854 cases, 922,958 controls, Supplementary Data 1). All subjects were of European descent. Genome-wide significance was determined using a tiered Bonferroni adjustment for variants classified by their expected impact (Methods)^18^.

Due to the complex course of development and clinical evaluation of back pain, IDD and dorsalgia are not mutually exclusive diagnoses^19,20^. In datasets where phenotype overlap could be studied (Iceland, Denmark and UKB), about 15–20% of dorsalgia cases also have an IDD diagnosis, while 30–45% of IDD cases have also received a dorsalgia diagnosis (Supplementary Tables 1–4). Using our data, we find that these comorbid back pain phenotypes are genetically correlated (*r*_*g*_ = 0.92, *P* < 1 × 10^−300^) (Supplementary Data 2). In line with previous studies, we find that both back pain diagnoses show genetic correlations with their most commonly reported risk factors including osteoarthritis, body mass index (BMI), bone mineral density (BMD) of lumbar spine, depression and stress (Supplementary Data 2). Notably, IDD is genetically correlated with height (*r*_*g*_ = 0.10, *P* = 1.3 × 10^−7^) whereas dorsalgia is not (*r*_*g*_ = −0.01, *P* = 0.48). The genetic correlation of dorsalgia with BMI (*r*_*g*_ = 0.28, *P* = 2.2 × 10^−50^) can therefore be explained by its correlation with body weight (*r*_*g*_ = 0.25, *P* = 3.8 × 10^−41^).

**Fig. 1:**
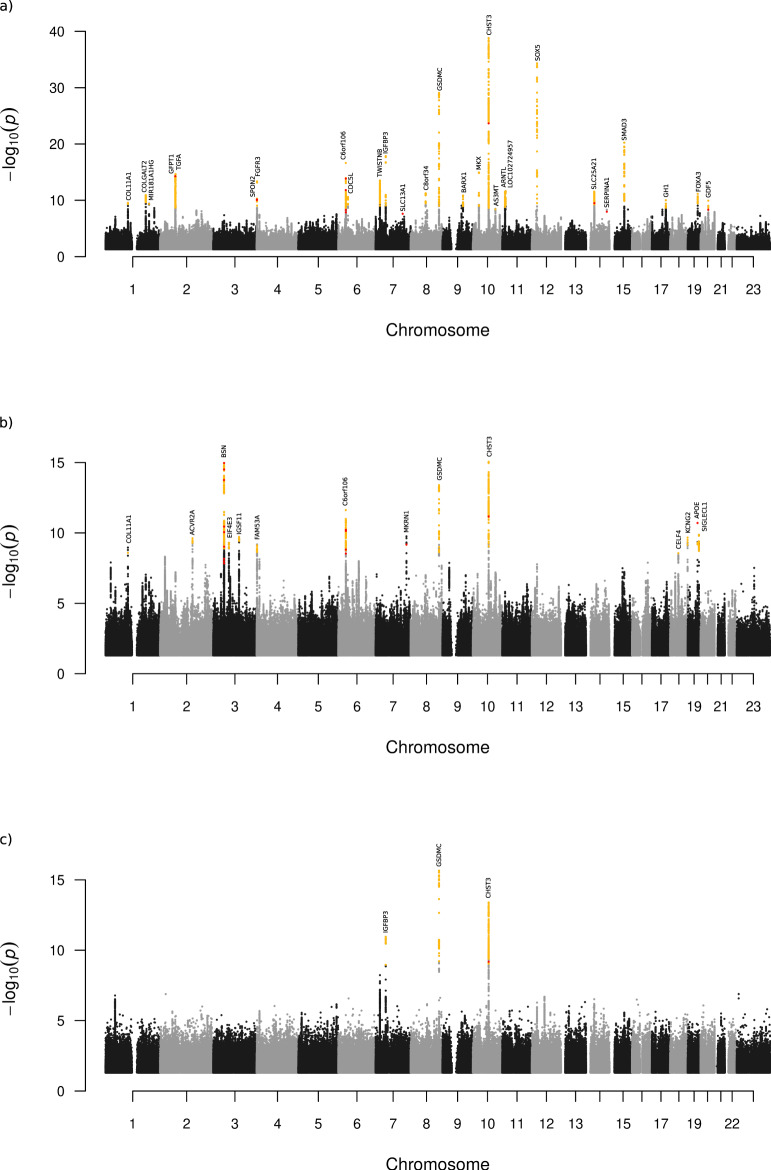
Manhattan plots showing results for meta-analyses of Intervertebral disc disorders (M51), dorsalgia (M54) and lumbar discectomy (LDHsurg). The *P* values (−log 10) from meta-analyses of the studied phenotypes are plotted (*y*-axis) against their respective positions on each chromosome (*x*-axis). **a** Intervertebral disc disorders IDD (M51), additive model (four cohorts; 58,854 cases, 922,958 controls), (**b**) Dorsalgia (M54), additive model (four cohorts, 119,110 cases, 909,847 controls), and (**c**) severe lumbar IDD defined by surgery (LDHsurg) (three cohorts; 9188 cases, 780,233 controls). *P* values are two sided and derived from a likelihood-ratio test. The gray and black dots represent SNPs not reaching genome-wide significance threshold weighted for variant impact^18^. The yellow dots represent genome-wide significant SNPs and the red dots represent genome-wide significant SNPs with moderate or high impact^18^ (Methods).

**Fig. 2:**
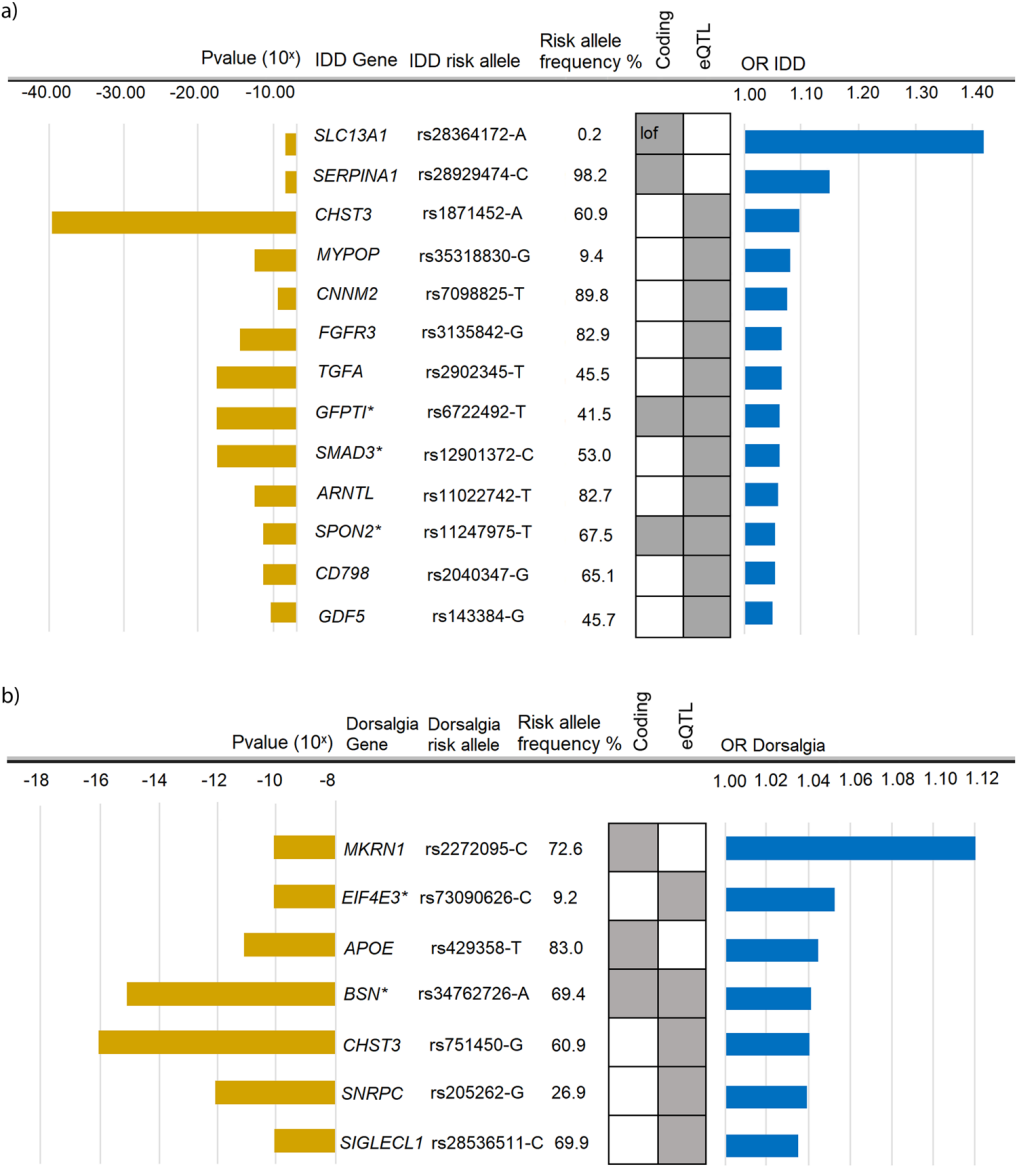
Genes likely to associate with (a) IDD and (b) Dorsalgia. Sequence variants associated with (**a**) IDD and (**b**) Dorsalgia for which functional evidence supports implication of genes in back pain. The variants listed are either protein-coding variants or affect mRNA expression (top cis-eQTL) as depicted by gray boxes (lof loss-of-function) (Supplementary Data 5–8). *Variants also associated in cis with mRNA of other genes (Supplementary Data 7,8). The meta-analyses were performed using logistic regression, the risk (odds ratio OR in yellow) of (**a**) IDD and (**b**) Dorsalgia are here shown for the risk-increasing allele and significance in blue. *COL11A1* and *GSDMC* are not included in the figure as evidence for their association with back pain was derived differently as described in results.

Under the additive model we identified 41 independent sequence variants associating with these back pain phenotypes at 33 loci, of which all but three loci are novel GWAS associations with back pain (Table 1, Fig. 1 and locus plots in Supplementary Figs. 1, 2). Variants at six loci associate with both IDD and dorsalgia, at 19 loci with IDD and at eight loci with dorsalgia (Table 1, Supplementary Data 3, 4). The three top IDD associations, at or near *CHST3*, *SOX5*, and *GSDMC* and the top two dorsalgia associations at *CHST3* and *GSDMC*, are the previously reported GWAS signals for back pain^12–14^. Conditional analyses identify secondary signals at 5 of the loci (*GFPT1*/*TGFA*, *SPON2*/*FGFR3*, *GSDMC*, *SMAD3*, and *KCNG2*) (Table 1). To highlight genes likely mediating the observed effects on back pain, we annotated the identified variants or variants within ±1 MB in high linkage disequilibrium (LD) (*r*^*2*^ ≥ 0.8), to assess if any are: (a) predicted to affect coding/splicing of a protein (VEP; variant effect predictor using Refseq gene set (https://www.ncbi.nlm.nih.gov/refseq/rsg/), Supplementary Data 5, 6), (b) correlate with mRNA expression (top local expression quantitative trait loci (cis-eQTL) in multiple tissues from deCODE, GTEx (https://gtexportal.org) and other public datasets (Supplementary Data 7, 8), and/or (c) correlate with plasma protein levels (top p-QTL) (Supplementary Data 9, 10, Methods). Together, these data highlight at least 19 genes with a functional link to back pain; one linked to both IDD and dorsalgia (*CHST3*), 13 linked to IDD and five to dorsalgia (Fig. 2). As the three previously published self-reported back pain signals were identified in data from UKB and the CHARGE consortium^12^, we also meta-analyzed separately the GWASs of Scandinavian (Icelandic, Danish, and Finnish) samples, and in these sets, excluding UKB data, we also replicate these three signals in both IDD and dorsalgia. Out of seven additional self-reported back pain signals, previously published but not replicated at the time^12^, we find support for four in the Scandinavian meta-analyses of IDD and dorsalgia (in *C8orf34*, *SPON2*, *DCC* and *HTRA1*) (Supplementary Data 11).

**Table 1:** **a)** Sequence variants associated with IDD (*N*_case_ = 58,854, *N*_ctrl_ = 922,958). **b)** Sequence variants associated with Dorsalgia (*N*_case_ = 119,110, *N*_ctrl_ = 909,847).

a) IDD Loci	Position (hg38)	rs name	EA^a^	OA^a^	Close gene	Annotation	Frq^b^	OR (95% CI)^c^	*P*^c^	*P*_bonf_^d^
**1p21.1**	chr1:102875460	rs4907985	A	T	*COL11A1*	Downstream	49.8	1.04 (1.03, 1.06)	3.9E−10	0.0044
1q23.5	chr1:183974675	rs3010044	C	A	*COLGALT2*	Intron	23.8	1.05 (1.04, 1.07)	2.0E−11	0.00045
1q32.1	chr1:198841735	rs71663412	T	TGA	*MIR181A1HG*	Indel	20.3	0.95 (0.93, 0.97)	4.7E−10	0.011
2p13.3	chr2:69345897	rs6722492*	T	C	*GFPT1*	Splice region	41.5	1.05 (1.04, 1.07)	2.2E−17	2.2E−11
**″**	chr2:70489467	rs2902345*	T	C	*TGFA*	Intron	45.5	1.05 (1.04, 1.07)	1.1E−17	2.6E−10
**4p16.3**	chr4:1171342	rs11247975*	G	T	*SPON2*	Missense	32.5	0.96 (0.94, 0.97)	6.5E−11	6.6E−05
**″**	chr4:1794909	rs3135842*	C	G	*FGFR3*	Intron	27.1	0.95 (0.93, 0.96)	4.0E−14	4.5E−07
**6p21.31**	chr6:34578783	rs2814982	T	C	*C6orf106*	Intergenic	11.9	1.09 (1.07, 1.11)	2.4E−17	1.6E−09
6p21.1	chr6:44478351	rs6929734	G	T	*CDC5L*	Intergenic	44.5	0.96 (0.94, 0.97)	2.6E−11	0.00059
7p21.1	chr7:19554541	rs2192477	G	A	*TWISTNB*	Intergenic	34.4	1.05 (1.04, 1.07)	5.3E−14	1.2E−06
7p12.3	chr7:45988978	rs1723939	T	C	*IGFBP3*	Intergenic	48.2	1.06 (1.05, 1.07)	1.6E−18	3.6E−11
7q31.32	chr7:123199913	rs28364172	A	G	*SLC13A1*	Stop gained	0.23	1.41 (1.25, 1.60)	2.5E−08	0.0053
8q13.2	chr8:68665402	rs16934882	A	C	*C8orf34*	Intron	19.9	1.06 (1.04, 1.07)	5.7E−12	0.00013
**8q24.21**	chr8:129707875	rs10110842*^e^	C	T	*GSDMC*	Regulatory region	27.4	1.07 (1.05, 1.09)	5.4E−08	>0.05
**″**	chr8:129726726	rs7826493*	G	A	*GSDMC*	Regulatory region	20.0	0.91 (0.89, 0.92)	2.8E−16	2.6E−22
9q22.32	chr9:93911476	rs58723578	T	C	*BARX1*	Intergenic	10.0	1.08 (1.06, 1.10)	1.4E−11	0.00033
10p12.1	chr10:27612430	rs2637326	G	T	*MKX*	Intergenic	51.1	0.95 (0.94, 0.96)	1.1E−15	2.6E−08
**10q22.1**	chr10:72012903	rs1871452	T	A	*CHST3*	3′UTR	39.1	0.92 (0.90, 0.93)	1.6E−39	1.8E−32
10q24.32	chr10:102868477	rs7098825	C	T	*AS3MT*	Upstream	10.2	0.94 (0.92, 0.96)	4.1E−09	0.047
11p15.3	chr11:13275014	rs11022742	C	T	*ARNTL*	Upstream	27.3	0.95 (0.94, 0.96)	4.3E−12	4.8E−05
11p15.2	chr11:15693077	rs4757353	C	T	*LOC102724957*	Intron	22.4	1.06 (1.04, 1.07)	2.4E−12	5.4E−05
12p12.1	chr12:23822285	rs12310519	T	C	*SOX5*	Intron	15.7	1.11 (1.10, 1.13)	4.8E−35	3.2E−27
14q13.3	chr14:36988829	rs28487989	C	T	*SLC25A21*	Intron	21.1	0.95 (0.93, 0.96)	3.2E−12	7.4E−05
14q32.13	chr14:94378610	rs28929474	T	C	*SERPINA1*	Missense	1.83	0.87 (0.83, 0.92)	1.1E−08	0.011
15q22.33	chr15:67078168	rs12901372*	G	C	*SMAD3*	Intron	47.0	0.94 (0.93, 0.95)	9.0E−17	2.0E−09
**″**	chr15:67083662	rs4776881*^e^	C	T	*SMAD3*	Intron	45.3	1.05 (1.03, 1.06)	7.2E−08	>0.05
17q23.3	chr17:63921519	rs2040347	A	G	*GH1*	Upstream	34.9	0.96 (0.94, 0.97)	9.8E−11	0.0011
**19q13.32**	chr19:45877067	rs35318830	G	T	*FOXA3*	Downstream	9.41	1.08 (1.05, 1.10)	7.3E−12	8.2E−05
20q11.22	chr20:35437976	rs143384	G	A	*GDF5*	5′UTR	45.7	1.04 (1.03, 1.06)	1.2E−10	0.0014

b) Dorsalgia Loci	Position (hg38)	rs name	EA^a^	OA^a^	Close gene	Annotation	Frq^b^	OR (95%CI)^c^	*P*^c^	*P*_bonf_^d^
1p21.1	chr1:102875460	rs4907985	A	T	*COL11A1*	Downstream	49.8	1.03 (1.02, 1.04)	2.6E−09	0.029
2q22.3	chr2:147879893	rs7560502	C	A	*ACVR2A*	Intron	17.6	0.96 (0.95, 0.97)	3.8E−10	0.0087
3p21.31	chr3:49651777	rs34762726	A	G	*BSN*	Missense	32.2	0.96 (0.95, 0.97)	1.1E−15	1.1E−09
3p13	chr3:71732370	rs73090626	C	T	*EIF4E3*	Upstream	9.24	1.05 (1.04, 1.07)	5.1E−10	0.0057
3q13.32	chr3:118057173	rs1995245	C	T	*IGSF11*	Intergenic	18.0	0.96 (0.95, 0.97)	2.5E−10	0.0056
4p16.3	chr4:1688915	rs4865462	G	A	*FAM53A*	Upstream	47.4	0.97 (0.96, 0.98)	7.8E−10	0.0089
6p21.31	chr6:34595387	rs205262	G	A	*C6orf106*	Intron	26.9	1.04 (1.03, 1.05)	2.4E−12	5.4E−05
7q34	chr7:140459051	rs2272095	G	C	*MKRN1*	Missense	27.4	0.97 (0.96, 0.98)	6.8E−10	0.0013
8q24.21	chr8:129707875	rs10110842*	C	T	*GSDMC*	Regulatory region	27.4	1.03 (1.02, 1.05)	2.2E−10	>0.05
″	chr8:129726726	rs7826493*	G	A	*GSDMC*	Regulatory region	20.0	0.96 (0.95, 0.97)	7.2E−14	3.1E−06
10q22.1	chr10:72001257	rs751450	A	G	*CHST3*	Intron	39.1	0.96 (0.95, 0.97)	1.0E−15	2.3E−08
18q12.2	chr18:37570563	rs9953231	A	G	*CELF4*	Upstream	22.8	1.03 (1.02, 1.05)	2.7E−09	0.031
18q23	chr18:79817501	rs71338065*	T	TCA	*KCNG2*	Intergenic	20.8	1.04 (1.03, 1.05)	2.2E−11	0.0005
″	chr18:79873271	rs76838079*^e^	T	C	*KCNG2*	Intron	16.5	0.97 (0.96, 0.98)	7.5E−08	>0.05
19q13.32	chr19:44908684	rs429358	C	T	*APOE*	Missense	17.0	0.96 (0.95, 0.97)	2.0E−11	2.0E−05
19q13.41	chr19:51270257	rs28536511	A	C	*SIGLECL1*	Downstream	30.1	0.97 (0.96, 0.98)	1.6E−10	0.0018

*Bold* are loci with marker significant in both IDD (Table 1a) and dorsalgia (Table 1b).

^a^Effect allele (EA) and other allele (OA).

^b^Average frequency of effect allele in the four cohorts.

^c^OR and *P* value for an inverse-variance weighted meta-analysis of association results for the four cohorts.

^d^*P* value after a variant class-specific Bonferroni adjustment^18^. *For this variant the *P* value and OR presented is adjusted for the effect of the other variant at the locus through conditional analysis.

^e^Second signal is not genome-wide significant. Results per cohort are in Supplementary Data 3, 4. Associations of these and correlated variants (*r*^2^≥ 0.8) with various traits listed in the GWAS catalog (https://www.ebi.ac.uk/gwas/) are in Supplementary Data 16 (IDD variants) and Supplementary Data 17 (dorsalgia variants).

Finally, we meta-analyzed GWASs of a subset of IDD diagnosed, i.e., those with the most homogenous and severe IDD phenotype available to us that is represented by painful herniated lumbar discs requiring surgery (LDHsurg). This phenotype was available for all cohorts except Finland resulting in a total 9188 cases and 780,323 controls. Results show three significant signals, all representing the top IDD signals at or near *GSDMC*, *CHST3* and *IGFBP3*, here with larger effects (Fig. 1, Table 2, Supplementary Data 12). The most significant association with LDHsurg, is with the regulatory region variant rs7833174 near *GSDMC* (OR = 0.851, *P* = 2.2 × 10^−16^), the same signal previously identified in association with the surgical IDD phenotype in Icelandic data only (rs6651255, *r*^*2*^ = 1, D′ = 1)^14^ and subsequently in GWAS meta-analyses of self-reported back pain (rs7814941, *r*^*2*^ = 0.90, D′ = 1)^12^.

**Table 2:** Variants associating with LDHsurg in GWAS meta-analysis of three cohorts; Iceland, UK Biobank and Finland (*N*_cases_ = 9188, *N*_controls_ = 780,323) compared to association with IDD (M51) in all four cohorts.

Loci	rs name	EA^a^	Close gene	Annotation	Frq^b^	OR_LDHsurg_ (95% CI)^c^	*P*_LDHsurg_^c^	OR _IDD_ (95% CI)^c^	*P*_IDD_^c^
8q24.21	rs7833174	C	*GSDMC*	Regulatory region	23.4	0.85 (0.82, 0.88)	2.1 × 10^−16^	0.96 (0.95, 0.97)	7.2 × 10^−14^
10q22.1	rs4148948	G	*CHST3*	3′UTR	38.2	0.88 (0.85, 0.91)	4.1 × 10^−14^	0.92 (0.90, 0.93)	1.6 × 10^−39^
7p12.3	rs1723939	T	*Near IGFBP3*	Regulatory region	50.2	1.12 (1.08, 1.15)	1.4 × 10^−11^	1.06 (1.05, 1.07)	1.6 × 10^−18^

^a^Effect allele (EA).

^b^Average frequency of effect allele in the three cohorts for which the surgical phenotype was available (Iceland, UKB, and Denmark).

^c^OR and *P* value for an inverse-variance weighted meta-analysis of association results for three cohorts (LDHsurg) and all four cohorts (IDD).

### Mendelian randomization analyses of IDD and dorsalgia

**Fig. 3:**
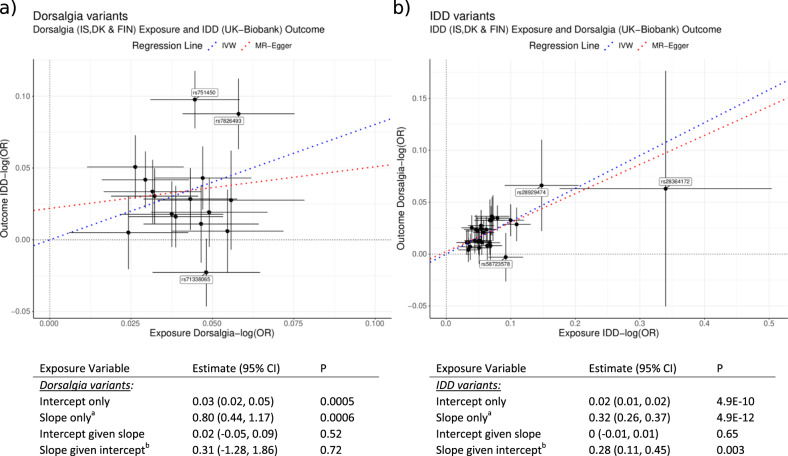
Mendelian randomization (MR) analyses of the genetic relationship between IDD and dorsalgia in terms of causality. **a** shows effects of variants associating with Dorsalgia at genome-wide significance, on IDD and dorsalgia. **b** shows the effects of variants associating with IDD at genome-wide significance, on IDD and dorsalgia. Effects are expressed as logarithms of odds ratios (log(OR)) and black crosses indicate 95% confidence intervals (CI) around effects. To avoid sample overlap, exposure effects are from the cohorts from Iceland (IS), Denmark (DK) and Finland (FIN), while outcome effects are from UK-Biobank. The dashed blue lines show the linear regression fit through the origin, weighting variants according to the square of the standard error of their effect estimates (also known as inverse-variance weighted, IVW)^a)^). The IVW-MR method is a multiplicative random effects model, where the test statistic is from a t-distribution, the test is two sided. No multiple comparison adjustments were made. The dashed red lines show the weighted linear regression fit not constrained to go through the origin (also known as MR Egger^b)^). For the IDD variants, the slopes of both regression lines are different from zero. For the dorsalgia variants, the slope of the regression line (IVW) through the origin is different from zero, but not the slope of the unconstrained regression line (MR Egger). Further, the effects of the dorsalgia variants deviate substantially more from the regression lines than the IDD variants. These results are not sensitive to outlier removal (Methods, Supplementary Fig. 3).

To explore the genetic relationship between IDD and dorsalgia in terms of causality, we performed Mendelian randomization (MR) analyses using the genome-wide significant IDD and dorsalgia variants (Table 1) as independent variables and studying their respective dorsalgia and IDD effects in non-overlapping samples^21^ (Methods). We find that variants associated with IDD at genome-wide significance, consistently also associate with dorsalgia (Fig. 3); the logarithm of ORs for dorsalgia was 0.32 times that of the logarithm of IDD ORs for these variants. Conversely, the variants associated with dorsalgia at genome-wide significance were enriched for variants also associating with IDD, but the strength of association with dorsalgia was not proportional to the association of these variants with IDD (Fig. 3).

### Rare LoF variants in *SLC13A1* confer high risk of IDD

A rare stop-gained variant (rs28364172-A, p.Arg12Ter) in *SLC13A1* (Solute carrier family 13 member 1) at 7q31.32 with a minor allele frequency (MAF) ranging from 0.07 to 0.32% in the four studied populations, confers the largest risk effect observed in this study. It associates with IDD (OR = 1.41, *P* = 2.5 × 10^−8^), and weaker with dorsalgia (OR = 1.14, *P* = 0.0066). *SLC13A1* encodes a 595-amino-acid protein that functions as a high-affinity sodium-dependent sulfate transmembrane transporter^22,23^. It is primarily expressed in the proximal renal tubules and small intestine, where it mediates the first step of sulfate (re)absorption^22,23^. We observe other rarer LoF variants in *SLC13A1*, the second most frequent being rs138275989 (p.Trp48Ter, MAF = 0.01–0.24%), that also associates with IDD (OR = 1.39, *P* = 1.2 × 10^−4^). Combined, *SLC13A1* LoF variants associate with IDD in a LoF burden test (OR = 1.44, *P* = 3.1 × 10^−11^), with comparable effects observed in the three datasets holding individual level genotypes (OR_Iceland_ = 1.57, *P* = 8.6 × 10^−4^; OR_UKB_ = 1.39, *P* = 1.9 × 10^−3^; OR_Denmark_ = 1.43, *P* = 1.0 × 10^−6^) (Supplementary Data 13).

A previous study in an Amish population reported that both p.Arg12Ter and p.Trp48Ter associate with reduced blood sulfate levels, or by 27.6% (*P* = 2.7 × 10^−8^) and 27.3% (*P* = 6.9 × 10^−14^) respectively, as well as jointly compared to non-carriers of either variant (*P* = 8.8 × 10^−20^)^24^. In a sample of 315 Icelanders with serum-sulfate measures, we replicate the sulfate-level association for p.Arg12Ter; finding that carriers have a 32.6% reduction compared to non-carriers (standardized effect = −1.5 SD, *P* = 0.0045), but could not test p.Trp48Ter as none of the Icelandic carriers had serum-sulfate measurements (Supplementary Data 14 and Supplementary Fig. 4). Consistently, loss of *SLC13A1* function associates with reduced sulfate availability in mice, sheep and dogs, under a recessive mode of inheritance and in these animal models, lack of sulfate links to a range of severe metabolic, musculoskeletal and neurological phenotypes^25–27^. However, no human disease associations have been reported for this gene in the GWAS catalog (https://www.ebi.ac.uk/gwas/)^28^ or OMIM (https://www.omim.org). In GWASs from Iceland and UKB we find evidence of p.Arg12Ter associating with sitting height (representing length of spine^29^) (Meta ß_Ice-UKB_ = −0.12 SD, *P* = 4.70 × 10^−7^, *N* = 416,923), but not with standing height (Meta ß_Ice-UKB_ = 0.04 SD, *P* = 0.41, *N* = 546,274). The rarer LoF variant p.Trp48Ter also affects sitting height (Meta ß_Ice-UKB_ = −0.09 SD, *P* = 0.02, *N* = 416,923). However, we found no other disease or disease-related associations with p.Arg12Ter or p.Trp48Ter heterozygotes, in UKB or Icelandic datasets. Traits tested were akin to those suggested by reports of *SLC13A1* LoF BMD, cholesterol levels, liver parameters, dehydroepiandrosterone levels, epilepsy autism, anxiety and depression (Supplementary Data 15). Among sequenced Icelanders, we identify four homozygous carriers of the p.Arg12Ter mutation. These are adults between 60 to 80 years old, all have children and according to available diagnostic data, all have accumulated several painful musculoskeletal diagnoses over their lifetimes and three out of the four have IDD (Supplementary Fig. 5).

The most significant signal in the GWAS of serum sulfate in Amish^24^ was a missense variant, rs148386572-A (p.Leu348Pro, MAF = 6%) in another sulfate transporter gene, *SLC26A1*, at 4p16.3 (Effect = −0.046 SD, *P* = 4.4 × 10^−12^). This sulfate transporter is located on the basolateral membrane of intestine and proximal tubules of kidneys that in addition to sulfate, also transports bicarbonate and oxalate (Supplementary Note 1). The missense variant in *SLC26A1* associates nominally with IDD in our meta-analysis (OR = 1.12, *P* = 0.012) (Supplementary Data 14 and Supplementary Fig. 4). No LoF mutations were identified in *SLC26A1* in our study.

### *CHST3* is another sulfate-related gene associating with back pain

The top signal in both IDD and dorsalgia is represented by 62 correlated variants (*r*^*2*^ ≥ 0.8) in and near the 3′UTR region of *CHST3* (*Carbohydrate sulfotransferase 3*). *CHST3* is widely expressed in tissues with highest expression in peripheral nerve tissue (GTEx, (https://gtexportal.org). CHST3 catalyzes sulfation of chondroitin, an extracellular matrix proteoglycan of various tissues and the major proteoglycan of cartilage and intervertebral discs^23^. The strongest association observed in this study is with a common variant in the 3′UTR region of *CHST3*, rs1871452-T (MAF = 39.1%) that associates with reduced risk of IDD (OR = 0.916, *P* = 1.57 × 10^−39^) and dorsalgia (OR = 0.962, *P* = 2.27 × 10^−16^). A correlated variant, rs3180-A (*r*^*2*^ = 0.51) has previously been shown to associate with self-reported back pain (OR = 0.946, *P* = 1.65 × 10^−11^)^12^. Other correlated *CHST3* variants (*r*^*2*^ = 0.81 and 1.00) associate with tall stature (rs12258400, *P* = 5.0 × 10^−16^)^30^ and early onset lumbar disc degeneration (rs4148941, *P* = 4.0 × 10^−8^)^16^. For the latter variant, rs4148941, which is fully correlated with our lead IDD variant, the allele that associates with protection against early onset lumbar disc degeneration (rs4148941-C) was reported to associate with higher CHST3 mRNA expression in intervertebral disc cells^16^. Analyzing RNA sequencing data from blood (we did not have access to intervertebral disc tissue) we find that our lead protective back pain variant, rs1871452-T, is the top cis-eQTL at this locus, associating with reduced CHST3 mRNA expression (Effect = −0.36 SD, *P* = 1.42 × 10^−166^, *N* = 13,175). The protective IDD variants at this locus thus affect expression of CHST3 mRNA in both blood and intervertebral disc tissue but in opposite direction (Supplementary Data 7,8). The location of the lead variant in 3′UTR of *CHST3* and other correlated variants at this locus, overlaps with those of microRNAs and other regulatory factor binding sites that may affect CHST3 mRNA expression and stability. We did not identify any cis-pQTLs at the locus (Supplementary Data 9).

### The top novel back pain signals

The most significant association with dorsalgia (OR = 0.97, *P* = 1.1 × 10^−15^) is at 3p21.3. It consists of 59 correlated (*r*^*2*^ ≥ 0.8) variants, represented by rs34762726-A, a common (MAF = 32.2%) missense variant (p.Ala741Thr) in *BSN* (*Bassoon presynaptic cytomatrix protein*). Among the correlated markers at this locus are several other missense variants in nearby genes; in *MST1* (*Macrophage stimulating 1*) (rs3197999-A, p.Arg703Cys, *r*^*2*^ = 0.98) and *GPX1* (*Glutathione peroxidase 1*) (rs1050450-A, p.Pro200Leu, *r*^*2*^ = 0.86), with comparable protective effects on dorsalgia (OR ~0.96). Incidentally, cis-eQTL (multiple tissues) and cis-pQTL (plasma protein) analyses suggest a number of likely mediation genes at the locus, including *MST1* and *GPX1*, as well as *APEH* (*Acylaminoacyl-peptide hydrolase*) that is the top cis-eQTL at this locus (Supplementary Data 8, 10). Thus, our results highlight a number of genes at this novel dorsalgia locus, however, without resolving which gene is the most likely culprit.

Previous studies have associated the *MST1* variant (rs3197999-A) with increased risk of inflammatory bowel disease (IBD) and other chronic inflammatory conditions, including ankylosing spondylitis; a form of spinal arthritis that can lead to back pain^31,32^. However, since the associated variants have opposing effects on IBD and dorsalgia it is unlikely that the association with dorsalgia is mediated through the painful IBD condition. To study the relationship between IBD and dorsalgia further, we performed a MR analysis, using 222 known IBD variants^32^ and found no evidence for a causal effect of IBD on dorsalgia (Supplementary Fig. 6), indicating pleiotropy, rather than a causal link between these traits.

The most significant novel IDD association is with rs12901372-G, a common (MAF = 42.7%) intronic variant in *SMAD3* at 15q22.33, (OR = 0.94, *P* = 5.6 × 10^−21^). The locus conferring protection against IDD associates with a 27.6% higher (*P* = 8.23 × 10^−19^) *SMAD3* RNA expression in muscle/skeletal tissue (top cis-eQTL) (Supplementary Data 7). *SMAD3* encodes one of a group of intracellular signaling proteins that play a role in the TGFB pathway. Rare missense and LoF mutations in this gene are linked to aneurysms-osteoarthritis syndrome and Loeys-Dietz Syndrome, a connective tissue disorder, under a dominant mode of inheritance (OMIM#603109, https://www.omim.org/entry/602931). This variant also associates with, hip, knee-, and spinal osteoarthritis with the same direction of effect as for IDD^33^ (Supplementary Data 16). Using as instruments 18 known osteoarthritis variants^34^, we studied their effects on IDD in non-overlapping samples^21^ (Methods), finding that as a group they exert causal effects on IDD (Inverse-Variance Weighted (IVW) estimate = 1.46 (1.05, 2.05), *P* = 0.04), but less on Dorsalgia (IVW estimate = 1.01 (1.00, 1.02), *P* = 0.005) (Supplementary Fig. 7).

### 19 back pain genes are highlighted

By annotation of the identified variants (or variants in high LD (*r*^*2*^ > 0.8 and within ±1MB), as being coding variants or variants affecting mRNA expression (cis-eQTL) or protein levels (cis- p-QTL), we identify 19 back pain genes (Fig. 2), of which 17 (all but *CHST3* and *GSDMC*) are new for back pain phenotypes. More genes are functionally associated with the etiologically more specific phenotype IDD than with the more heterogenous phenotype dorsalgia. For IDD these include *SERPINA1* (*Serpin family A member 1*), that encodes a serine protease inhibitor belonging to the serpin superfamily whose targets include elastase, plasmin, thrombin, trypsin, chymotrypsin, and plasminogen activator; the transcription factor *MYPOP* (*Myb-related transcription factor, partner of profilin*); *CNNM2* (*Cyclin M2*) that encodes a transmembrane protein involved in magnesium transport; *FGFR3* (*Fibroblast growth factor receptor 3*) and *TGFA* (*Transforming growth factor alpha*), both encoding growth factors involved in bone development; *GFPT1*, encoding *Glutamine fructose-6-phosphate amidotransferase 1*, which is the first and rate-limiting enzyme of the hexosamine biosynthetic pathway and has been linked to recessive congenital myasthenic syndrome and synthesis of proteoglycans^35^. Of the six novel dorsalgia genes, five associate more strongly with dorsalgia than IDD, including *MKRN1* (*Makorin ring finger protein-1*), an E3 ubiquitin ligase involved in protein homeostasis of Eag1 potassium channels^36^, *EIF4E3* (*Eukaryotic translation initiation factor 4E family member 3*) and *SNRPC* (*Small nuclear ribonucleprotein polypetide C*); both widely expressed in tissues and involved in mRNA translation. *SIGLECL1* (Sialic acid-binding immunoglobulin-like lectin 12) encodes a cell surface protein of the Ig superfamily and is mainly expressed in the immune system^37^.

We note that among the new back pain genes highlighted in this study is *APOE*. The missense variant (p.Cys130Arg, rs429358-C), representing the *APOE4* allele that increases risk of Alzheimer’s disease^38^, also associates with dorsalgia (OR = 0.96, *P* = 1.97 × 10^−11^), but not with IDD (OR = 0.99, *P* = 0.20). The reduced risk of dorsalgia associated with this variant is consistent across all four datasets with *P*_*het*_ = 0.148 (Supplementary Data 4) (For additional details on IDD and dorsalgia genes, see Supplementary Note 2).

Finally, we find other sources of evidence pointing to two additional back pain genes; *GSDMC* and *COL11A1*. In addition to the *CHST3* locus, two other variants showed significant associations with both IDD and dorsalgia; the intergenic signals near *GSDMC* and the novel back pain variant downstream of *COL11A1* (Table 1). The primary and secondary signals close to *GSDMC* are both located in distal enhancers for *GSDMC*, suggesting they may affect transcription of *GSDMC* at this locus. In terms of the *COL11A1* association, it is the only gene at the locus (see locus plots in Supplementary Figs. 1, 2) and encodes one of three α-chains that are building blocks for Type-XI collagen, a cartilage-specific extracellular matrix protein^39^.

## Discussion

Back pain is considered a symptom rather than a disease, and for the vast majority of individuals affected, it is not possible to identify the cause of back pain or a specific nociceptive source^6^. Here we study two diagnostically defined back pain phenotypes; one associated with an identified pathogenesis i.e., secondary to IDD, and the other, dorsalgia, representing severe back pain of heterogenous origins that is largely non-diagnostic of an underlying pathology^6^. Although these phenotypes are highly genetically correlated, MR analyses show that while IDD variants consistently associate with dorsalgia, and variants associating with dorsalgia are enriched for IDD variants, the strength of their association with dorsalgia was not proportional to the association of these variants with IDD. In other words, while IDD is diagnosed in the context of back pain and can result in a dorsalgia diagnosis, dorsalgia is a phenotype governed by other genetic properties than its association with IDD. By analyzing these phenotypes separately in GWAS meta-analyses, we identified in total 41 sequence variants at 33 loci associated back pain, the majority with IDD. All but three loci are novel back pain associations and fine-mapping, annotation and functional studies highlight 19 genes likely mediating the effects of the associated variants on the development of IDD and/or dorsalgia. For comparison with the IDD phenotype, we performed a GWAS meta-analysis of the etiologically most specific and painful IDD phenotype available to us; herniated lumbar discs requiring surgery (LDHsurg), confirming the top three signals identified in association with all IDD. In addition to the previously detected signal near *GSDMC*^14^, which remains the top LDHsurg signal, the *CHST3* signal and the intergenic signal near *IGFBP3* reached GW significance. All three confer somewhat larger effects on this surgical phenotype than on IDD in general. *IGFBP3* encodes insulin-like growth factor binding protein 3 that has been shown to play a role both in the inflammatory processes and bone destruction observed in rheumatoid arthritis, and is considered a therapeutic agent candidate for treatment of this autoimmune and inflammatory disease^40^. Several genes identified by our IDD and dorsalgia associations have also been implicated in inflammatory processes and consequential pain involved in the pathogenesis of osteoarthritis, such as the *GSDMC*, *CHST3*, *SERPINA1*, *SPON2*, *SMAD3*, *TGFA*, *GDF5*, *COL11A1*, and *COL2A1*^33,34^. Indeed, by MR analysis indicates that as a group, osteoarthritis variants do have causal effects on IDD and to a lesser extent on dorsalgia, although evidently other mechanisms are also involved.

Importantly, our results also point to other proteins as potential therapeutic or preventive targets. As such, the *SLC13A1* LoF variants that associate with back pain secondary to IDD and with reduced serum-sulfate, are of special interest. Sulfate is the fourth most abundant anion in human plasma with normal serum levels between 0.3 and 0.5 mM, and plays an important role in numerous physiological processes^22,23^. Sulfate availability in blood is regulated by the apical sodium-sulfate co-transporter (Nas1) encoded by *SLC13A1*, and on the basolateral membrane, by the sulfate-anion transporter 1 encoded by *SLC26A1*. Both are primarily expressed in the intestine (duodenum to colon) where dietary sulfate is absorbed and in the proximal tubules of kidneys where reabsorption occurs^22,23^. The sulfonation of glycosaminoglycans in human articular cartilage, which requires the enzyme encoded by *CHST3*, appears to be very sensitive to even small deviations in sulfate concentration^41^.

The polyanionic nature of chondroitin sulfates within the intervertebral disc, allows the disc tissue to maintain disc hydration and thereby disc height by retaining water and interacting with growth factors and cytokines^42^. The association of the *SLC13A1* LoF variants with decreased sitting height (a proxy for spinal height), but not with standing height, spinal BMD or osteoarthritis, is consistent with their effects on spinal length being through decreased height of the intervertebral discs, rather than the cartilage or bones of the spinal column. Depletion of chondroitin sulfates, although also a process of normal ageing, can be expedited by lack of enzymatic activity or sulfate availability, resulting in decreased disc hydration, loss of fluid movement, cell apoptosis, and consequently loss of disc function^42^, in some, but not all cases resulting in pain^9^. In addition to sulfate’s importance for maintaining proteoglycans of cartilage and bone, it is also involved in the biotransformation of multiple compounds including neurotransmitters, drugs and hormones^24^. Sulfonation leads to inactivation of steroids and plays a major role in liver detoxification of several drugs, including the commonly used pain-medication acetaminophen^24^. Furthermore, the importance of sulfate for human fetal development is evidenced by elevation in maternal plasma sulfate levels in pregnancy^43,44^.

Despite its impact on human health, sulfate is almost never measured clinically^23^. Our findings raise the question whether screening for reduced sulfate levels could identify those that would benefit from supplementation. Dietary supplements such as chondroitin-sulfate for osteoarthritis, have been shown to slow cartilage breakdown of affected joints and reduce pain^45^. Future studies are needed to address the potential preventive or therapeutic role of sulfate supplementation to reduce risk of IDD or other conditions related to sulfate metabolism.

Pain is defined as “An unpleasant sensory and emotional experience associated with, or resembling that associated with, actual or potential tissue damage”^46^. While the majority of variants identified in this study associate with pain secondary to deterioration of intervertebral discs and/or the adjacent vertebral endplates, it is also evident from clinical studies that extent of tissue damage does not correlate with the perception or progression of pain^9–11^. Pain is ultimately experienced in the brain upon reception of nociceptive signals from the peripheral nervous system. Of the variants identified in this study, about half are in or near genes expressed in the brain. These include *FGFR3*, the gene encoding *fibroblast growth factor receptor 3* that influences development of cortical and hippocampal neurons^47^, *KCNG2*, encoding a voltage-gated potassium channel expressed in hippocampus and harboring variants influencing educational attainment^48^, depression^49^ and response to opiates^50^, and *GH1*, expressed in the pituitary and linked to hypersensitivity to pain and chronic pain development^51^. Future studies are needed to address what roles these, and other genes suggested by our findings, have in the development of back pain.

In summary, using a genome-wide approach we have identified 41 variants associating with back pain secondary to IDD and/or of unknown etiology (dorsalgia). Co-localization studies and other data implicate several specific genes and their products involved in the biology of back pain, including *CHST3* and *SLC13A1* that highlight the key role of sulfate in the underlying processes leading to painful IDD.

## Methods

### Study samples and ethics declarations

Icelandic data for this study were analyzed under National Bioethics Committee (NBC) Licenses #VSN-17-035 and #VSN-12-162 (with amendments), issued following review by the Icelandic Data Protection Authority (DPA). Participants donated blood or buccal samples under informed consent allowing the use of their samples and data in NBC-approved projects at deCODE Genetics. All personal identifiers of participants’ data were encrypted by a third-party system (IPS-Identity Protection System^52^) approved and monitored by the Icelandic DPA. The phenotype data were obtained in collaboration with Icelandic physicians, from diagnostic data repositories of the Landspitali National University Hospital in Reykjavik, Iceland, the Registry of Primary Health Care Contacts, and the Registry of Contacts with Medical Specialists in Private Practice, spanning the years 1983-2017. The primary phenotypes analyzed were defined by physician-assigned International Classification of Diseases ICD-10 codes^53^; M54 Dorsalgia and M51 Other IDD.

The UK Biobank (UKB) study is a large prospective cohort study of ~500,000 study volunteers from across the UK who were 40–69 years old at time of recruitment in 2006-2011^54^. The UKB phenotype and genotype data were collected following an informed consent and the study is overseen by The North West Research Ethics Committee that reviewed and approved UKB’s scientific protocol and operational procedures (REC Reference Number: 06/MRE08/65). Data for this study were obtained and research conducted under the UKB application license number 24898. The phenotypes were defined by International Classification of Diseases (ICD-10) codes^53^; M54 Dorsalgia and M51 Other IDD, obtained from General Practice (GP) clinical event records and other sources (Field IDs 42040, 131929 and 131925) and hospital diagnoses (Field IDs 41270 and 41271). Of the about 500,000 participants in the UKB study, 408,653 were genotypically verified of white British/European descent and included in this study.

Danish samples were obtained through collaboration with the Danish Blood Donor Study (DBDS) and the Copenhagen Hospital Biobank (CHB). The Danish Blood Donor Study (DBDS) GWAS study is a large prospective cohort study of ~110,000 blood donors across Denmark^55^. The Danish Data Protection Agency (P-2019-99) and the Danish National Committee on Health Research Ethics (NVK-1700704) approved the studies under which genetic data on DBDS participants were obtained. The DBDS data requested for this study was approved by the DBDS steering committee. Patients with IDD and dorsalgia were genotyped under the Genetics of pain and degenerative disease protocol approved by the Danish National Committee on Health Research Ethics (NVK-1803812) and the Danish Protection Agency (P-2019-51). CHB is a research sample repository, which contains left-over samples obtained from diagnostic procedures on hospitalized and outpatient patients in the Danish Capital Region hospitals. Samples from the CHB were included as part of the study on pain-related diseases under the genetics of pain and degenerative musculoskeletal disease protocol (NVK-1803012).

Finnish data were obtained from the FinnGen project (https://www.finngen.fi/en), which gathers samples and phenotype data from a nationwide network of Finnish biobanks and national health registers. The Coordinating Ethics Committee of the Helsinki and Uusimaa Hospital District evaluated and approved the FinnGen research project which complies with existing legislation (in particular the Biobank Law and the Personal Data Act). The official data controller of the study is the University of Helsinki. The summary statistics for GWASs on IDD (M51) and dorsalgia (M54), were imported on November 30, 2020 from a source available to consortium partners (version 3; http://r3.finngen.fi). Sample sizes and variants analyzed for each cohort are listed in Supplementary Data 1.

### Genotyping and imputation

Genotyping and imputation in Icelandic samples were performed at deCODE Genetics in Iceland, using methods described in detail by Jonsson et al.^56^ and Gudbjartsson et al.^57^. In short, a large fraction of the 360,000 inhabitants in Iceland have participated in various studies at deCODE. At the time of this study, deCODE had sequenced whole genomes of 49,962 Icelanders using GAIIx, HiSeq, HiSeqX, and NovaSeq Illumina technology to a mean depth of at least 17.8×. SNPs and insertions and deletions (indels) were identified and their genotypes called using joint calling with Graphtyper^58^. Genotype calls were improved by using information about haplotype sharing, taking advantage of the fact that all sequenced individuals had also been chip-typed and long-range phased. Over 38 million sequence variants that passed high-quality thresholds (all variants with info >0.8) were then imputed into 166,281 Icelanders who had been genotyped with various Illumina SNP chips and their genotypes phased using long-range phasing methods^59^. In Icelandic data, we used genealogic information, to impute sequence variants into relatives of the chip-typed to further increase the sample size for association analysis and increase power to detect associations. To account for inflation in test statistics due to stratification or cryptic relatedness, we applied LD-score regression^60^.

Chip-typing of Danish samples was performed using the Illumina Infinium Global Screening Array. Quality control, and subsequent imputation of CHB and DBDS samples was performed at deCODE genetics. In total, over 332,000 samples from the CHB and DBDS, together with ~238,000 genotyped samples from North-western Europe were long-range phased using Eagle2^61^. Samples and variants with less than 98% yield were excluded. We used the same methods described above for the Icelandic data^56,57^, to create a haplotype reference panel by phasing previously whole-genome sequenced Danish genotypes (*N* = 8635) using phased chip data (*N* = 332,949), and to impute the genotypes from the haplotype reference panel into the phased chip data.

Samples of UKB participants were genotyped with a custom-made Affymetrix chip, UK BiLEVE Axiom, in the first 50,000 individuals^62^, and the Affymetrix UK Biobank Axiom array in the remaining participants^63^. Imputation was performed by the Welcome Trust Center for Human Genetics using a combination of the Haplotype Reference Consortium^64^ and the UK10K haplotype resources^65^, and 1000Genomes phase 3 panels^66^. A total of ~38.0 million variants were analyzed in the UKB dataset (Supplementary Data 1).

A custom-made FinnGen ThermoFisher Axiom array (>650,000 SNPs) was used to genotype ~135,600 FinnGen samples at ThermoFisher genotyping service facility in San Diego. Genotype calls were made with AxiomGT1 algorithm (https://finngen.gitbook.io/documentation/methods/genotype-imputation). Imputation was performed using the Finnish population-specific and high coverage WGS backbone and the population-specific SISu v3 imputation reference panel with Beagle 4.1. A total of 14.5 million variants were analyzed in the Finnish dataset (Supplementary Data 1).

### Association analyses

To test for association between sequence variants and IDD and dorsalgia and using software developed at deCODE genetics^57^, we performed logistic regression assuming the additive model using the Icelandic, UKB, and Danish data for each phenotype in each dataset respectively, and then combined in meta-analyses with the GWAS results acquired from FinnGen. We used LD-score regression to account for distribution inflation due to cryptic relatedness and population stratification in the Icelandic, UKB, and Danish data^60^. In the Icelandic association analyses, we adjusted for sex, county of origin, current age or age at death (first and second order term included), genotype availability for the individual, and an indicator function for the overlap of the lifetime of the individual with the time span of phenotype collection. In the UKB association analyses, we adjusted for sex, age, and the first 40 principal components to adjust for population stratification. In the Danish association analyses, we adjusted for sex and the first 20 principal components. The FinnGen association analyses were adjusted for sex, age, the genotyping batch, and the first 10 principal components.

### GWAS meta-analyses

For the meta-analyses, we used a fixed-effects inverse-variance method^67^ to combine results from the four datasets in which each dataset was assumed to have a common OR but allowed to have different population frequencies for alleles and genotypes. Variants with imputation information below 0.8 were excluded from the analyses. Sequence variants were mapped to NCBI Build38 and matched on position and alleles to harmonize the four GWAS datasets for each meta-analysis (see Supplementary Data 1 for variants analyzed per cohort). We estimated the genome-wide significance threshold and corrected for multiple testing with a Bonferroni procedure weighted for variant classes and predicted functional impact^18^. The adjusted significance thresholds were 1.95 × 10^−7^ for variants with high impact, 3.91 × 10^−8^ for variants with moderate impact, 3.55 × 10^−9^ for low-impact variants, 1.78 × 10^−9^ for low-impact variants in DNase I hypersensitivity sites and 5.92 × 10^−10^ for all other variants, including those in intergenic regions. The primary signal at each genomic locus was defined as the sequence variant with the lowest Bonferroni adjusted *P* value using the adjusted significance thresholds described above and in Table 1. Conditional analyses were performed to identify possible secondary signals, on all variants within 500 kb from index variants (*P* < 1 × 10^−8^, excluding the HLA region), based on linkage disequilibrium (LD) results from 8700 whole-genome sequenced Icelandic individuals. We also tested whether the lead signals in the IDD and dorsalgia GWASs associated with other diseases in Iceland, UKB, Denmark and Finland and in combined meta-analyses assuming multiplicative model, as above. A linear mixed-model implemented by BOLT-LMM^68^ was used to test for association between the IDD and dorsalgia associated variants and quantitative traits, assuming an additive genetic model. For quantitative measurements, we assume they follow a normal distribution with a mean that depends linearly on the expected allele at the variant and a variance-covariance matrix proportional to the kinship matrix^68^. We used LD-score regression^60^ to account for inflation in test-statistics due to cryptic relatedness and stratification. We used a likelihood-ratio test to compute *P* values.

### Genetic correlations and Mendelian randomization

We calculated genetic correlations between pairs of diseases selected on the basis of being among the most commonly reported risk factors for back pain (Supplementary Data 2) as follows: We used cross-trait LD-score regression and summary statistics from traits in the deCODE and UKB datasets or available meta-analyses. In these analyses, we used results for about 1.2 million well imputed variants, and for LD information we used precomputed LD scores for European populations (downloaded from: https://data.broadinstitute.org/alkesgroup/LDSCORE/eur_w_ld_chr.tar.bz2).

To avoid bias due to overlapping samples, we calculated the genetic correlation between a meta-analysis of Icelandic and Danish data sets for dorsalgia and IDD and the UKB summary statistics (osteoarthritis, BMI, height, weight, DXA area L1234), or a meta-analysis of UKB data and GEFOS^69^ for BMD of the lumbar spine, and between a meta-analysis of UKB and Finnish data sets for dorsalgia and IDD and the deCODE summary statistics (osteoarthritis, BMI, height, weight, DXA area L1234), or a meta-analysis of deCODE and Danish data sets for BMD of the lumbar spine. The results of the two analyses were subsequently meta-analyzed. For major depressive disorder and stress, we calculated the genetic correlation between published meta-analyses and our meta-analysis of Icelandic, Danish, UKB and Finnish data sets for dorsalgia and IDD.

To assess genetic relationships between IDD and dorsalgia with regards to causality, we performed MR analyses using the genome-wide significant variants for each trait respectively as instruments^70^. We used linear regression without an intercept term, weighted by the inverse-variance of the outcome associations (inverse-variance weighted, IVW), MR coupled with an intercept test, and weighted linear regression with an intercept term, usually referred to as MR-Egger. To avoid sample overlap^21^, exposure effects were from the cohorts from Iceland (IS), Denmark (DK) and Finland (FIN) while outcome effects were from UK-Biobank (see Supplementary Data 1 for numbers of cases and controls). To assess the sensitivity of our MR analysis to outliers, we also ran the results with an outlier removal method (Supplementary Fig. 3). Similarly, to evaluate the causal effects of OA variants on IDD, we performed MR analysis using as instruments 18 osteoarthritis variants^34^ and studied their effects from 16 OA GWASs on individuals of European descent with total cases *N* = 78,610 and controls *N* = 100,164. For the MR analysis on the causal effects of IBD variants on dorsalgia, we used as instruments 222 IBD variants^32^ with effects from 15 IBD GWASs and Immunochip meta-analysis on individuals of European descent with total cases *N* = 38,155 and controls *N* = 48,485.

### Functional data

To highlight genes associating with IDD and/or dorsalgia, we use various functional data, including annotation of the identified variants or variants in high linkage disequilibrium (LD) (*r*^*2*^ ≥ 0.8 and within ±1 MB) that are predicted to affect protein coding or splicing (VEP; variant effect predictor using Refseq gene set (https://www.ncbi.nlm.nih.gov/refseq/rsg/), mRNA expression (top local expression quantitative trait loci i.e., cis-eQTL in multiple tissues from deCODE, GTEx (https://gtexportal.org) and other public datasets, and/or plasma protein correlations (p-QTL) (Supplementary Data 5–10).

#### Transcriptomics

We performed RNA sequencing of 14,248 genes in whole blood samples from 13,175 Icelanders and of 9396 genes in subcutaneous adipose tissue samples from 700 Icelanders. We computed gene expression based on personalized transcript abundances^71^. Association between variants and gene expression was estimated using a generalized linear regression, assuming additive genetic effect and quantile normalized gene expression estimates, adjusting for measurements of sequencing artefacts, demographic variables, blood composition, and hidden covariates^72^.

#### Proteomics

We used SomaLogic^®^ SOMAscan (version 4) proteomics assay to test association of identified IDD and dorsalgia sequence variants with protein levels in plasma. The assay scanned 4907 aptamers that measure 4719 proteins in samples from 35,559 Icelanders who also have contributed genetic data to NBC-approved projects at deCODE genetics^73^. Plasma protein levels were standardized and adjusted for year of birth, gender, and year of sample collection (2000–2019).

#### Gene set enrichment analysis

We performed a gene-based and gene set enrichment analysis using MAGMA^74^, as implemented by FUMA v.1.3.2^75^ (Supplementary Note 3).

### Reporting summary

Further information on research design is available in the Nature Research Reporting Summary linked to this article.

## Supplementary information

Supplementary Information

Description of Additional Supplementary Files

Supplementary Data 1-17

Reporting Summary

## Data Availability

The GWAS summary statistics from this study are available at deCODE’s summary statistics repository, https://www.decode.com/summarydata/. Other data generated or analyzed in this study are included in this article and its Supplementary Data and Information Files. Source data underlying the main figures is provided in Supplementary Data.

## Code availability

We used the following publicly available software to analyze data: GraphTyper (v2.0-beta, GNU GPLv3 license) is available at https://github.com/DecodeGenetics/graphtyper. Eagle2 is available at http://www.hsph.harvard.edu/alkes-price/software/. BOLT-LMM is available at http://www.hsph.harvard.edu/alkes-price/software/. R (version 3.6.3) is available at https://www.r-project.org/, R package ggplot for visualization (version 3.3.3), is available at https://ggplot2.tidyverse.org/, R package (v1.0.9) for GSMR is available at https://cnsgenomics.com/software/gsmr/. MAGMA (v1.08) is available at http://ctglab.nl/software/magma and FUMA at https://fuma.ctglab.nl/. No custom code was written for this study.
