# Author Correction: Rare *SLC13A1* variants associate with intervertebral disc disorder highlighting role of sulfate in disc pathology

**DOI:** 10.1038/s41467-022-30129-6

**Published:** 2022-04-27

**Authors:** Gyda Bjornsdottir, Lilja Stefansdottir, Gudmar Thorleifsson, Patrick Sulem, Kristjan Norland, Egil Ferkingstad, Asmundur Oddsson, Florian Zink, Sigrun H. Lund, Muhammad S. Nawaz, G. Bragi Walters, Astros Th. Skuladottir, Sigurjon A. Gudjonsson, Gudmundur Einarsson, Gisli H. Halldorsson, Valgerdur Bjarnadottir, Gardar Sveinbjornsson, Anna Helgadottir, Unnur Styrkarsdottir, Larus J. Gudmundsson, Ole B. Pedersen, Thomas Folkmann Hansen, Thomas Werge, Karina Banasik, Anders Troelsen, Soren T. Skou, Lise Wegner Thørner, Christian Erikstrup, Kaspar Rene Nielsen, Susan Mikkelsen, DBDS Genetic Consortium, Steffen Andersen, Søren Brunak, Kristoffer Burgdorf, Henrik Hjalgrim, Gregor Jemec, Poul Jennum, Per Ingemar Johansson, Kasper Rene Nielsen, Mette Nyegaard, Mie Topholm Bruun, Ole Birger Pedersen, Khoa Manh Dinh, Erik Sørensen, Sisse Ostrowski, Pär Ingemar Johansson, Daniel Gudbjartsson, Hreinn Stefánsson, Unnur Þorsteinsdóttir, Margit Anita Hørup Larsen, Maria Didriksen, Susanne Sækmose, GO Consortium, Eleftheria Zeggini, Konstantinos Hatzikotoulas, Lorraine Southam, Arthur Gilly, Andrei Barysenka, Joyce B. J. van Meurs, Cindy G. Boer, André G. Uitterlinden, Unnur Styrkársdóttir, Lilja Stefánsdóttir, Helgi Jonsson, Thorvaldur Ingvarsson, Tõnu Esko, Reedik Mägi, Maris Teder-Laving, Shiro Ikegawa, Chikashi Terao, Hiroshi Takuwa, Ingrid Meulenbelt, Rodrigo Coutinho de Almeida, Margreet Kloppenburg, Margo Tuerlings, P. Eline Slagboom, Rob R. G. H. H. Nelissen, Ana M. Valdes, Massimo Mangino, Aspasia Tsezou, Eleni Zengini, George Alexiadis, George C. Babis, Kathryn S. E. Cheah, Tian T. Wu, Dino Samartzis, Jason Pui Yin Cheung, Pak Chung Sham, Peter Kraft, Jae Hee Kang, Kristian Hveem, John-Anker Zwart, Almut Luetge, Anne Heidi Skogholt, Marianne B. Johnsen, Laurent F. Thomas, Bendik Winsvold, Maiken E. Gabrielsen, Ming Ta Michael Lee, Yanfei Zhang, Steven A. Lietman, Manu Shivakumar, George Davey Smith, Jonathan H. Tobias, April Hartley, Tom R. Gaunt, Jie Zheng, J. Mark Wilkinson, Julia Steinberg, Andrew P. Morris, Ingileif Jonsdottir, Aron Bjornsson, Ingvar H. Olafsson, Elfar Ulfarsson, Josep Blondal, Arnor Vikingsson, Soren Brunak, Sisse R. Ostrowski, Henrik Ullum, Unnur Thorsteinsdottir, Hreinn Stefansson, Daniel F. Gudbjartsson, Thorgeir E. Thorgeirsson, Kari Stefansson

**Affiliations:** 1 deCODE Genetics/Amgen, Inc., Reykjavik, Iceland; 2 Faculty of Medicine, School of Health Sciences, University of Iceland, Reykjavik, Iceland; 3 School of Engineering and Natural Sciences, University of Iceland, Reykjavik, Iceland; 4 Landspitali University Hospital, Reykjavik, Iceland; 5 Department of Clinical Immunology, Zealand University Hospital, Køge, Denmark; 6 Department of Clinical Medicine, Faculty of Health and Medical Sciences, University of Copenhagen, Copenhagen, Denmark; 7 Danish Headache Center, Dept. Neurology, Rigshospitalet-Glostrup, Glostrup, Denmark; 8 Novo Nordisk Foundation Center for Protein Research, Faculty of Health and Medical Sciences, University of Copenhagen, Copenhagen, Denmark; 9 Institute of Biological Psychiatry, Mental Health Services, Copenhagen University Hospital, Copenhagen, Denmark; 10 Lundbeck Foundation for GeoGenetics, GLOBE Institute, University of Copenhagen, Copenhagen, Denmark; 11 Department of Orthopaedic Surgery, CAG ROAD—Research OsteoArthritis Denmark, Copenhagen University Hospital, Hvidovre, Denmark; 12 Research Unit for Musculoskeletal Function and Physiotherapy, Department of Sports Science and Clinical Biomechanics, University of Southern Denmark, Odense, Denmark; 13 The Research Unit PROgrez, Department of Physiotherapy and Occupational Therapy, Næstved-Slagelse-Ringsted Hospitals, Næstved, Denmark; 14 Department of Clinical Immunology, Copenhagen University Hospital, Copenhagen, Denmark; 15 Department of Clinical Immunology, Aarhus University Hospital, Aarhus, Denmark; 16 Department of Clinical Immunology, Aalborg University Hospital, Aalborg, Denmark; 17 Department of Neurosurgery, Landspitali University Hospital, Reykjavik, Iceland; 18 Health Care Institution of West Iceland, Stykkisholmur, Iceland; 19 Statens Serum Institut, Copenhagen, Copenhagen, Denmark; 20 Department of Finance, Copenhagen Business School, Copenhagen, Denmark; 21 Department of Clinical Neurophysiology, University of Copenhagen, Copenhagen, Denmark; 22 Department of Biomedicine, Aarhus University, Aarhus, Denmark; 23 Department of Clinical Immunology, Odense University Hospital, Odense, Denmark; 24 Institute of Translational Genomics, Helmholtz Zentrum München, German Research Center for Environmental Health, Neuherberg, Germany; 25 Department of Internal Medicine, Erasmus MC, Medical Center, Rotterdam, The Netherlands; 26 Department of Orthopedic Surgery, Akureyri Hospital, Akureyri, Iceland; 27 Estonian Genome Center, Institute of Genomics, University of Tartu, Tartu, Estonia; 28 Laboratory for Bone and Joint Diseases, RIKEN Center for Integrative Medical Sciences, Tokyo, Japan; 29 Laboratory for Statistical and Translational Genetics, RIKEN Center for Integrative Medical Sciences, Kanagawa, Japan; 30 Department of Biomedical Data Sciences, Section Molecular Epidemiology, Leiden University Medical Center, Leiden, The Netherlands; 31 Departments of Rheumatology and Clinical Epidemiology, Leiden University Medical Center, Leiden, The Netherlands; 32 Department of Orthopaedics, Leiden University Medical Center, Leiden, The Netherlands; 33 Faculty of Medicine and Health Sciences, School of Medicine, University of Nottingham, Nottingham, Nottinghamshire UK; 34 Department of Twin Research and Genetic Epidemiology, Kings College London, London, UK; 35 Laboratory of Cytogenetics and Molecular Genetics, Faculty of Medicine, University of Thessaly, Larissa, Greece; 36 4th Psychiatric Department, Dromokaiteio Psychiatric Hospital, Haidari, Athens, Greece; 37 1st Department of Orthopaedics, KAT General Hospital, Athens, Greece; 38 2nd Department of Orthopaedics, National and Kapodistrian University of Athens, Medical School, Nea Ionia General Hospital ‘Konstantopouleio’, Athens, Greece; 39 School of Biomedical Sciences, The University of Hong Kong, Pokfulam, Hong Kong, China; 40 Department of Psychiatry, Li Ka Shing Faculty of Medicine, The University of Hong Kong, Pokfulam, Hong Kong, China; 41 Department of Orthopaedics and Traumatology, The University of Hong Kong, Pokfulam, Hong Kong, China; 42 Li Ka Shing Faculty of Medicine, The University of Hong Kong, Pokfulam, Hong Kong, China; 43 Department of Epidemiology, Harvard T.H. Chan School of Public Health, Boston, MA USA; 44 Department of Medicine, Brigham and Women’s Hospital, Boston, MA USA; 45 K. G. Jebsen Center for Genetic Epidemiology, Department of Public Health and Nursing, Faculty of Medicine and Health Sciences, Norwegian University of Science and Technology, Trondheim, Norway; 46 Department of Clinical and Molecular Medicine, Norwegian University of Science and Technology, Trondheim, Norway; 47 Genomic Medicine Institute, Geisinger Health System, Danville, PA USA; 48 Musculoskeletal Institute, Geisinger Health System, Danville, PA USA; 49 Department of Biostatistics, Epidemiology and Informatics, Perelman School of Medicine, University of Pennsylvania, Philadelphia, PA USA; 50 MRC Integrative Epidemiology Unit (IEU), Bristol Medical School, University of Bristol, Oakfield House, Oakfield Grove, Bristol, UK; 51 Musculoskeletal Research Unit, Translation Health Sciences, Bristol Medical School, University of Bristol, Southmead Hospital, Bristol, UK; 52 Department of Oncology and Metabolism and Healthy Lifespan Institute, University of Sheffield, Sheffield, UK; 53 Centre for Genetics and Genomics Versus Arthritis, Centre for Musculoskeletal Research, University of Manchester, Manchester, UK

**Keywords:** Genome-wide association studies, Pain, Bone

Correction to: *Nature Communications* 10.1038/s41467-022-28167-1, published online 2 February 2022.

The original version of this Article contained an error in Fig. 3, in which the blue and red trend lines on the left plot were incorrect. In addition, the text “Dorsalgia variants” in the table should have been italicized and underlined. The correct version of Fig. 3 is:
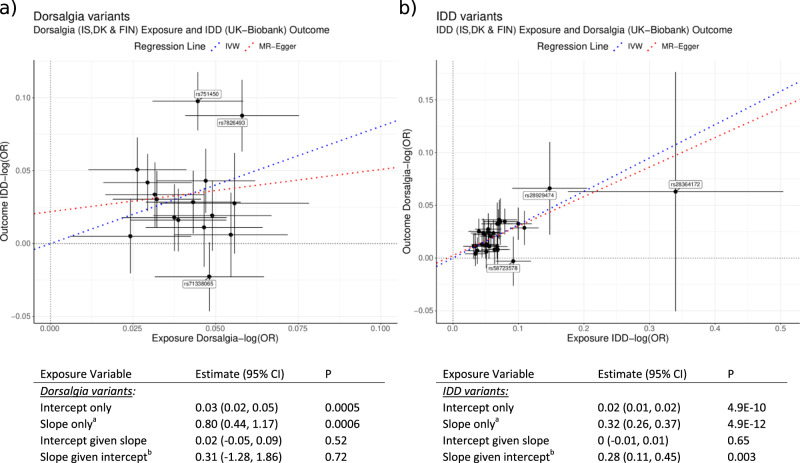


which replaces the previous incorrect version:
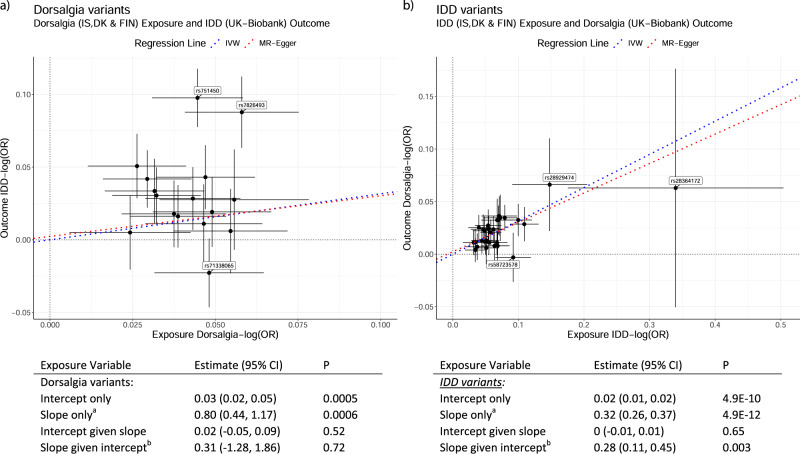


This has been corrected in both the PDF and HTML versions of the Article.

