# Supplementary material for: Rare SLC13A1 variants associate with intervertebral disc disorder highlighting role of sulfate in disc pathology
Source: Nat Commun. 2022 Feb 2;13:634. doi: 10.1038/s41467-022-28167-1 (PMC8810832; doi:10.1038/s41467-022-28167-1)
Supplement: Supplementary file 1 — Supplementary Information [file 41467_2022_28167_MOESM1_ESM.pdf]

Supplementary Information for Bjornsdottir et al.:

Rare *SLC13A1* mutations associate with intervertebral disc disorder highlighting role of sulfate in disc pathology

Contents

Supplementary Information Tables 2
Table 1. Diagnostic codes used to define phenotype M54 Dorsalgia 2
Table 2. Diagnostic codes used to define phenotype M51 IDD 3
Table 3. Distribution of sub-diagnoses within M51 and M54 4
Table 4. Phenotype overlap by study sample 5
Supplementary Information Figures 6
Figure 1. Locus plots for lead IDD variants 6
Figure 2. Locus plots for lead Dorsalgia variants 21
Figure 3. Mendelian Randomization Sensitivity Analysis 30
Figure 4. *SLC13A1* and *SLC26A2* variants' association with IDD and sulfate levels 31
Figure 5. Diagnoses and prescribed drugs among *SLC13A1* p.Arg12Ter homozygotes 32
Figure 6. Mendelian randomization: Inflammatory Bowel Disease (IBD) and dorsalgia 33
Figure 7. Mendelian randomization: Osteoarthritis – IDD & Dorsalgia 34
Supplementary Notes 36
Note 1. Role of *SLC13A1* and *CHST3* in sulfate availability and sulfation in proteoglycan synthesis 36
Note 2. Back pain variants with functional evidence implicating specific genes 36
Note 3. Gene set enrichment and pathway analyses 40
3.1. Intervertebral disc disorder (IDD) gene sets 41
3.2. Dorsalgia gene sets 46
References 49

Supplementary Information Tables

Table 1. Diagnostic codes used to define phenotype M54 Dorsalgia

For all datasets, phenotypes were defined by diagnostic codes assigned by physicians in clinical settings using the International statistical classification of diseases and related health problems: tenth revision[1] (ICD10: https://icd.who.int/browse10/2010/en#). In Denmark, the 8th revision of the ICD classification was also used by physicians, so for the Danish dataset phenotypes were also defined by corresponding ICD8 codes (ICD8: http://www.wolfbane.com/icd/icd8.htm).

**Coding system**	**Code**	**Diagnosis**<br>*Incl:.*	**Exclusions**
ICD10<br><br>ICD8	M54<br><br>728	**Dorsalgia**<br><br>**Vertebrogenic pain syndrome**	Psychogenic dorsalgia (F45.4)
ICD10	M54.0	**Panniculitis affecting regions of neck and back**	panniculitis:<br>• NOS (M79.3)<br>• lupus (L93.2)<br>• relapsing (Weber-Christian) (M35.6)
ICD10	M54.1	**Radiculopathy**<br>Neuritis or radiculitis:<br>• brachial NOS<br>• lumbar NOS<br>• lumbosacral NOS<br>• thoracic NOS<br>Radiculitis NOS	neuralgia and neuritis NOS (M79.2)<br>radiculopathy with:<br>• cervical disc disorder (M50.1)<br>• lumbar and other intervertebral disc disorder (M51.1)<br>• spondylosis (M47.2)
ICD10	M54.2	**Cervicalgia**	cervicalgia due to intervertebral cervical disc disorder (M50.-)
ICD10	M54.3	**Sciatica**	lesion of sciatic nerve (G57.0)<br>sciatica:<br>• due to intervertebral disc disorder (M51.1)<br>• with lumbago (M54.4)
ICD10	M54.4	**Lumbago with sciatica**	that due to intervertebral disc disorder (M51.1)
ICD10	M54.5	**Low back pain**<br>Loin pain<br>Low back strain<br>Lumbago NOS	lumbago:<br>• due to intervertebral disc displacement (M51.2)<br>• with sciatica (M54.4)
ICD10	M54.6	**Pain in thoracic spine**	pain due to intervertebral disc disorder (M51.-)
	M54.8	**Other dorsalgia**	
	M54.9	**Dorsalgia, unspecified**<br>Backache NOS	

Table 2. Diagnostic codes used to define phenotype M51 IDD

For all datasets, phenotypes were defined by diagnostic codes assigned by physicians in clinical settings using the International statistical classification of diseases and related health problems: tenth revision[1] (ICD10: https://icd.who.int/browse10/2010/en#). In Denmark, the 8th revision of the ICD classification was also used by physicians, so for the Danish dataset phenotypes were also defined by corresponding ICD8 codes (ICD8: http://www.wolfbane.com/icd/icd8.htm).

**Coding system**	**Code**	**Diagnosis**<br>*Incl.:*	**Exclusions**
ICD10<br><br><br><br><br>ICD8	M51<br><br><br><br><br>725	**Other intervertebral disc disorders,**<br>thoracic, thoracolumbar and lumbosacral disc disorders<br><br>**Displacement of intervertebral disk**	lumbar radiculitis NOS (M54.1)
ICD10	M51.0	**Lumbar and other intervertebral disc disorders with myelopathy** (G99.2*)	
ICD10	M51.1	**Lumbar and other intervertebral disc disorders with radiculopathy** (G55.1*)<br>Sciatica due to intervertebral disc disorder	lumbar radiculitis NOS (M54.1)
ICD10	M51.2	**Other specified intervertebral disc displacement**<br>Lumbago due to displacement of intervertebral disc	
ICD10	M51.3	**Other specified intervertebral disc degeneration**	
ICD10	M51.4	**Schmorl nodes**	
ICD10	M51.8	**Other specified intervertebral disc disorders**	
ICD10	M51.9	**Intervertebral disc disorder, unspecified**	

Table 3. Distribution of sub-diagnoses within M51 and M54

The distribution of subcategories within each ICD10 code studied were not available for all datasets. In the UK Biobank (from Data Showcase on UKB website, date June 15th, 2020).

M51 Other intervertebral disk disorders N = 13,517:

- M51.0 Lumbar and other intervertebral disk disorders with myelopathy N = 412 (3.0%)
- M51.1 Lumbar and other intervertebral disk disorders with radiculopathy N = 4928 (36.5%)
- M51.2 Other specified intervertebral disk displacement N = 3828 (28.3%)
- M51.3 Other specified intervertebral disk degeneration N= 3885 (28,7%)
- M51.4 Schmorl's nodes N = 54 (0.4%)
- M51.8 Other specified intervertebral disk disorders N = 224 (1.7%)
- M51.9 Intervertebral disk disorder, unspecified N = 186 (1.4%)

M54 Dorsalgia N = 30,367:

- M54.0 Panniculitis affecting regions of neck and back N = 30 (0.1%)
- M54.1 Radiculopathy N = 1507 (5.0%)
- M54.2 Cervicalgia N = 3010 (9.8%)
- M54.3 Sciatica 3049 N = (10.0%)
- M54.4 Lumbago with sciatica 961 N = (3.2%)
- M54.5 Low back pain N =13840 (45.6%)
- M54.6 Pain in thoracic spine N = 420 (1.4%)
- M54.8 Other dorsalgia N = 276 (0.9%)
- M54.9 Dorsalgia, unspecified N = 7274 (24.0%)

The UK Biobank distribution of M54 subcodes is comparable to the proportions of diagnostic codes within M54 reported in a study of 10,843 Germans seen in family practice and diagnosed with M54 Dorsalgia[2]. The distribution of diagnoses under M54 at the four-digit level in Germans were as follows and as in the UK Biobank cohort, the majority (61.6%) with lumbago or lower back pain:

- M54.0 Panniculitis, neck and back - 0.3%
- M54.1 Radiculopathy – 20.1%
- M54.2 Cervicalgia – 8.2%
- M54.3 Sciatica – 2.5%
- M54.4 Lumbago with sciatica – 28.5%
- M54.5 Low back pain – 33.1%
- M54.6 Pain in thoracic spine – 1.8%
- M54.8 Other dorsalgia – 1.4%
- M54.9 Dorsalgia, unspecified – 4.1%

Table 4. Phenotype overlap by study sample

Below are listed percentages of cases with diagnostic codes M54 and M51 (horizontally in the table), who also have received the alternate diagnosis, M54 or M51 (vertically in the table)

M51-M54 overlap in UK Biobank

% of cases also with:	M54	M51
M54	-	45.9
M51	14.8	-

M51-M54 overlap in Iceland

% of cases also with:	M54	M51
M54	-	29.4
M51	15.6	-

M51-M54 overlap in UK Biobank

% of cases also with:	M54	M51
M54	-	34.1
M51	20.9	-

Supplementary Information Figures

Figure 1. Locus plots for lead IDD variants

Following are regional visualization figures (Locus Plots) of the IDD lead variants. The leading variant at each locus is colored in black. Other variants are colored by the degree of correlation (r^2) with the lead variant (rsname and chromosomal position hg38_other allele_effect allele). The − log10 *P*-values on the left y-axis (two-sided logistic regression) are plotted for each variant against their chromosomal position (x-axis). The right y-axis shows calculated recombination rates based on Icelandic data at the chromosomal location, plotted as solid black lines.

rs4907985

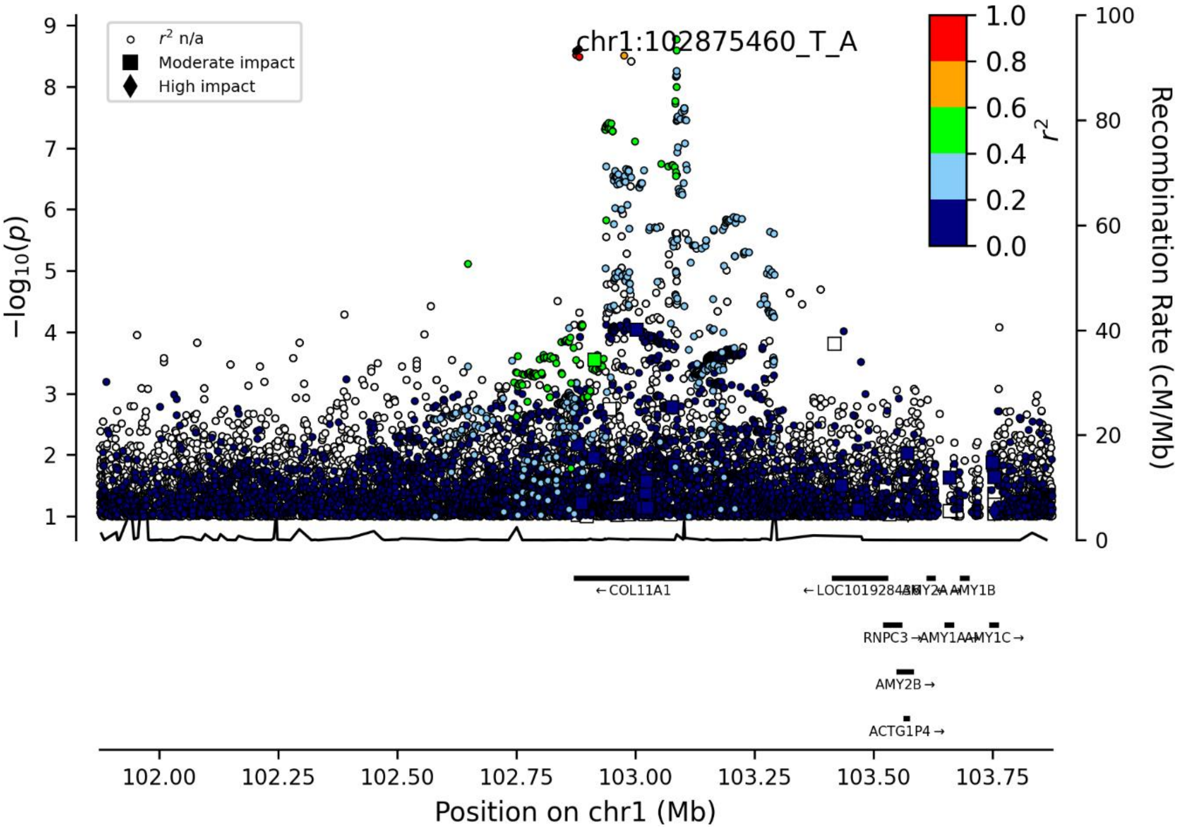

rs3010044

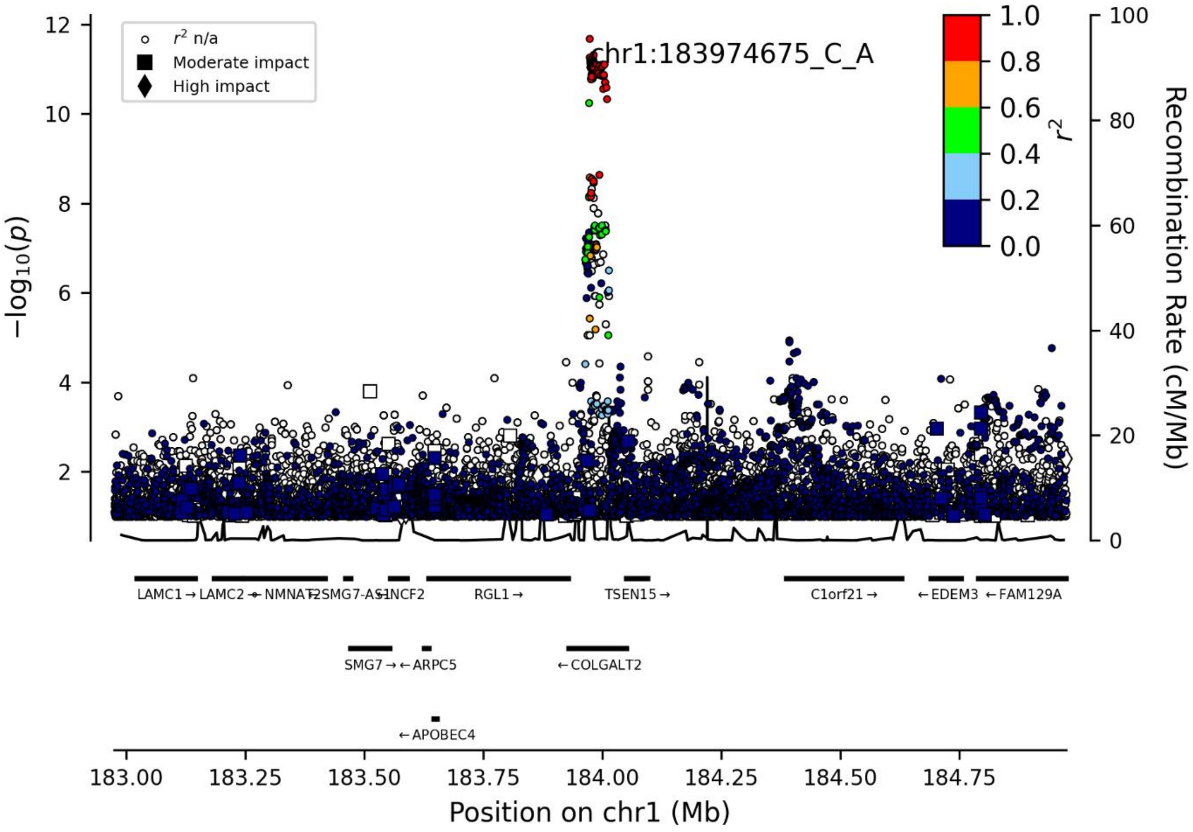

rs71663412

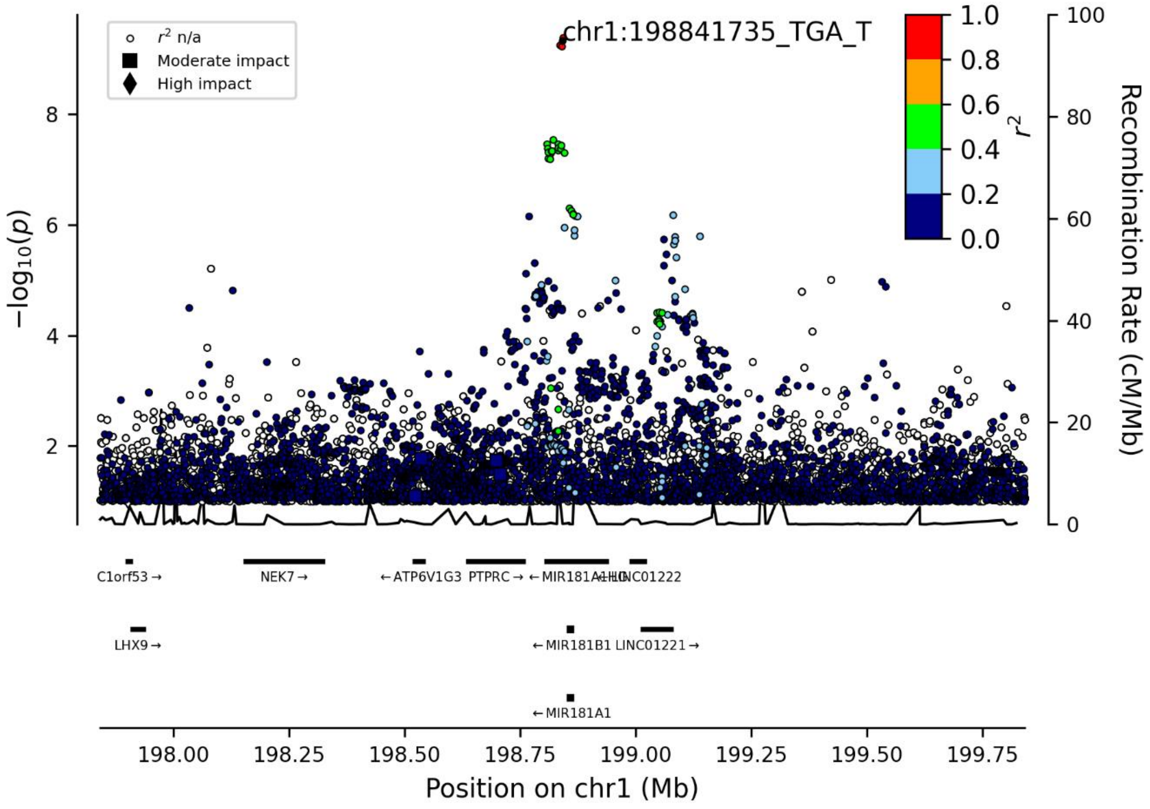

rs6722492

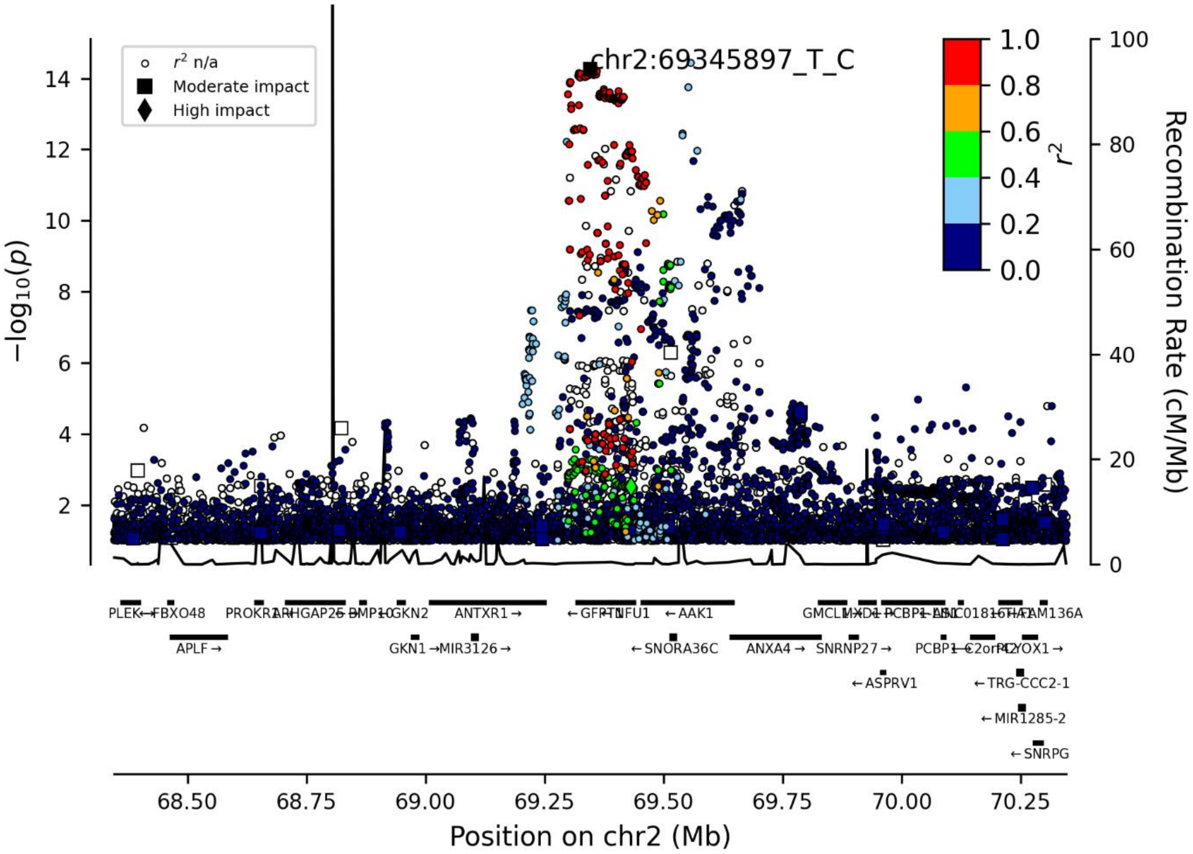

rs2902345

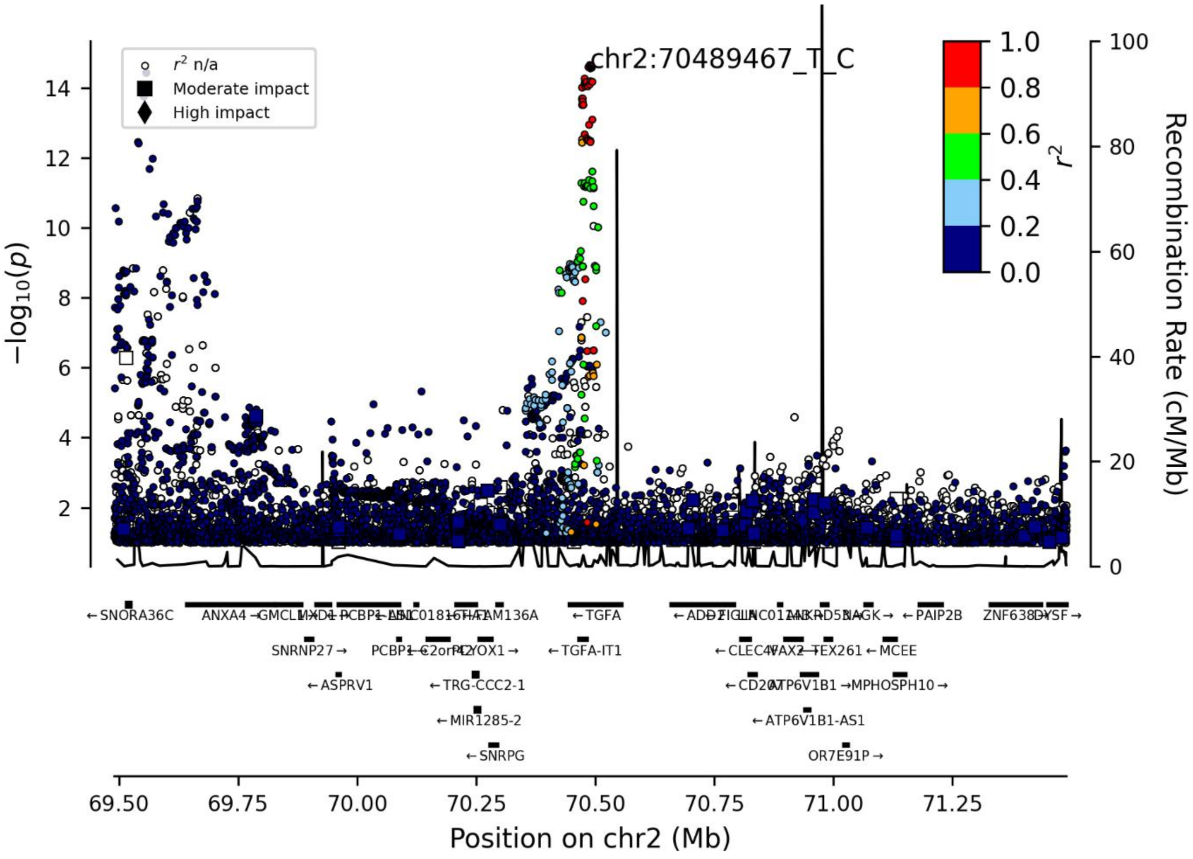

rs11247975

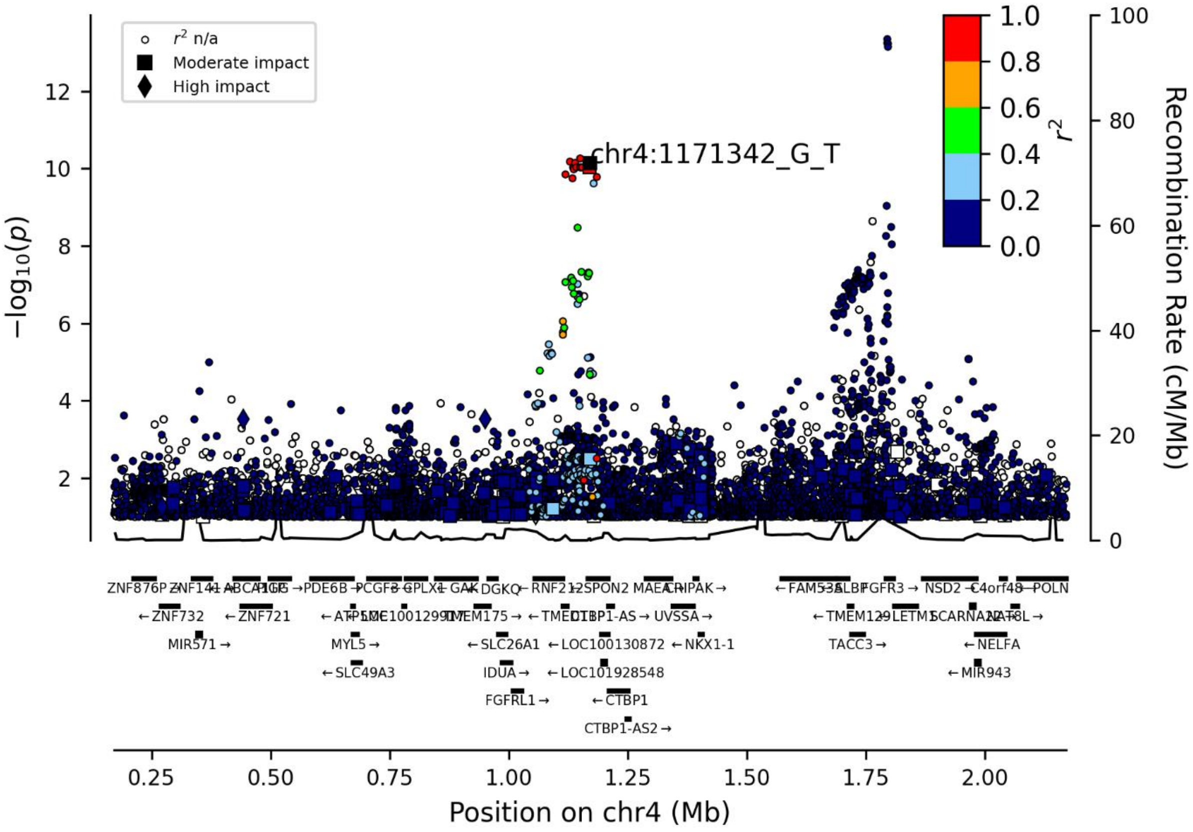

rs3135842

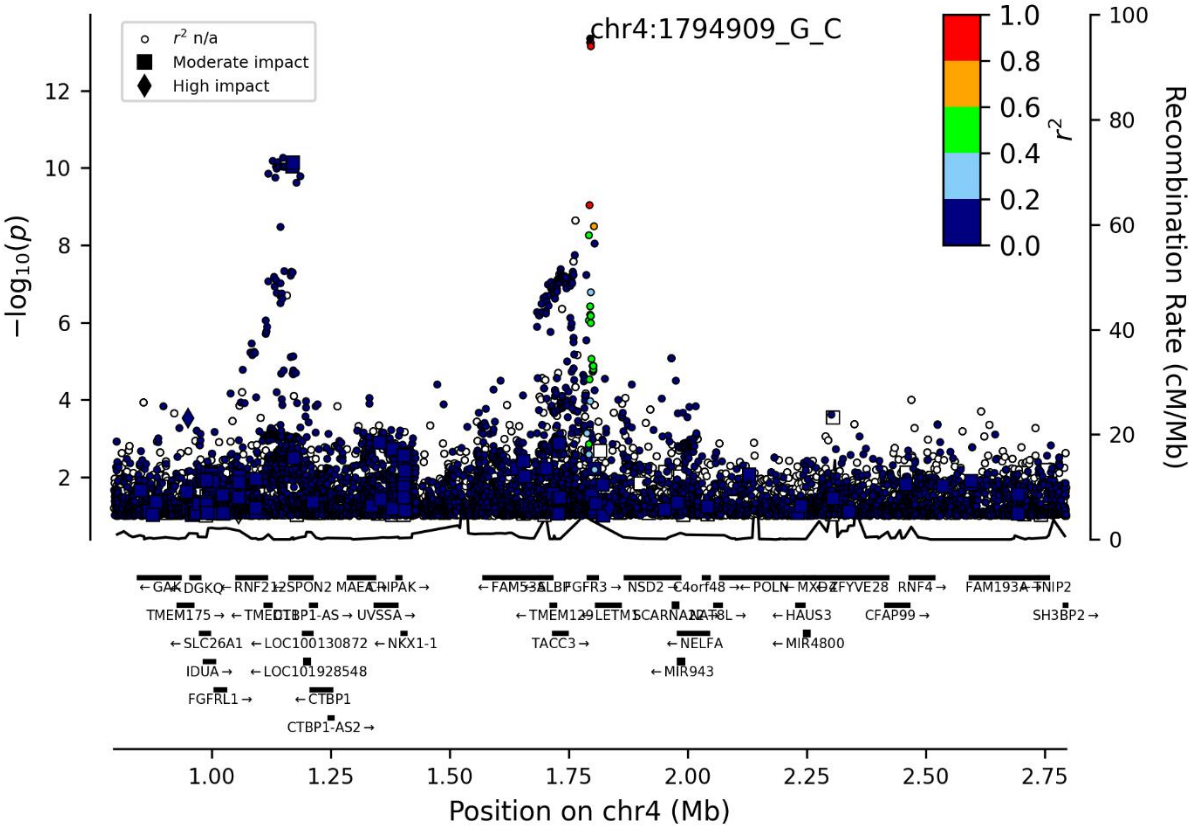

rs2814982

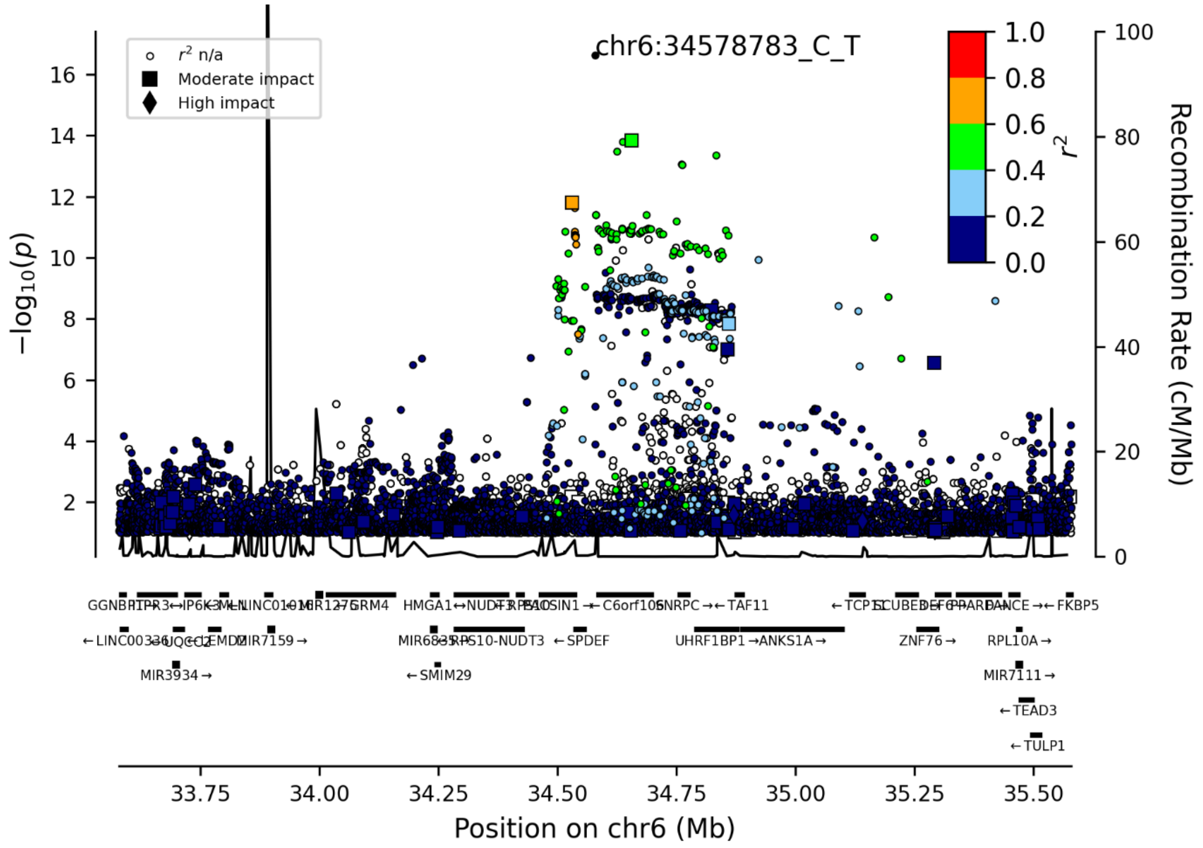

rs6929734

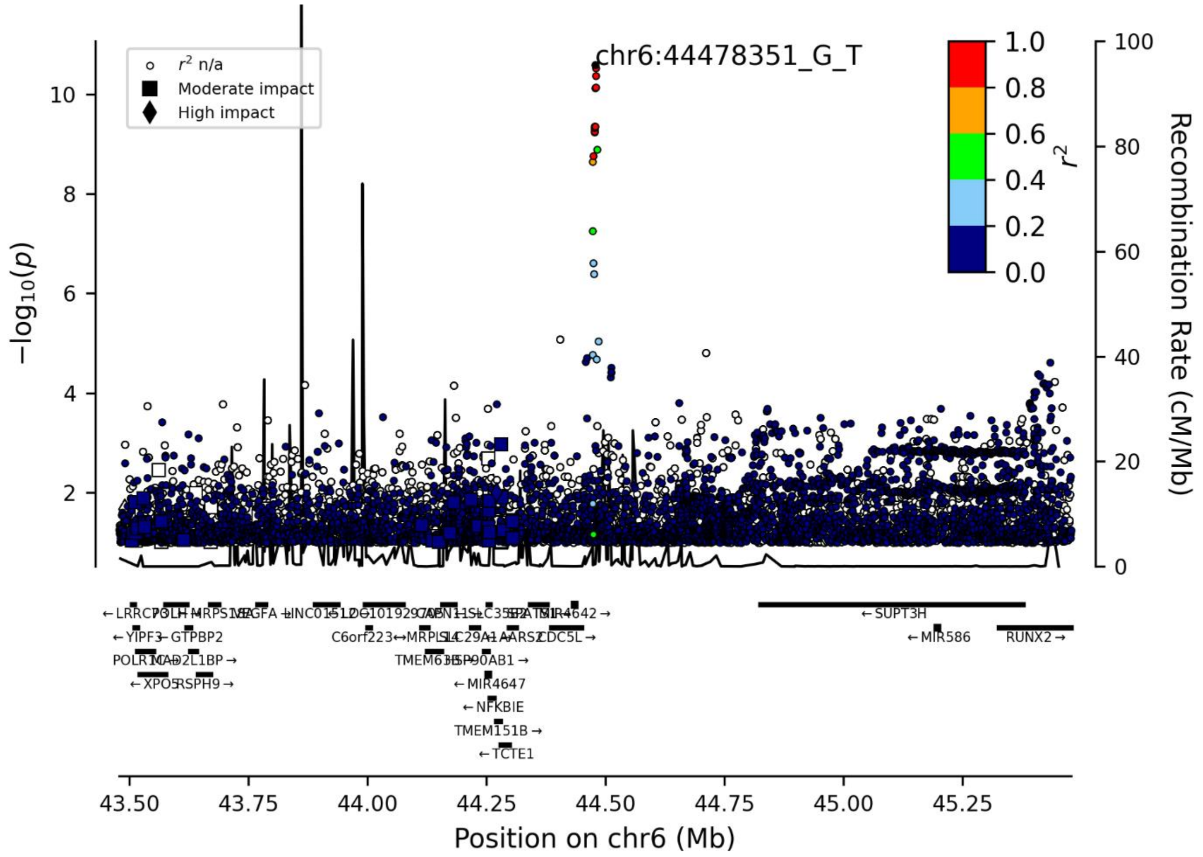

rs2192477

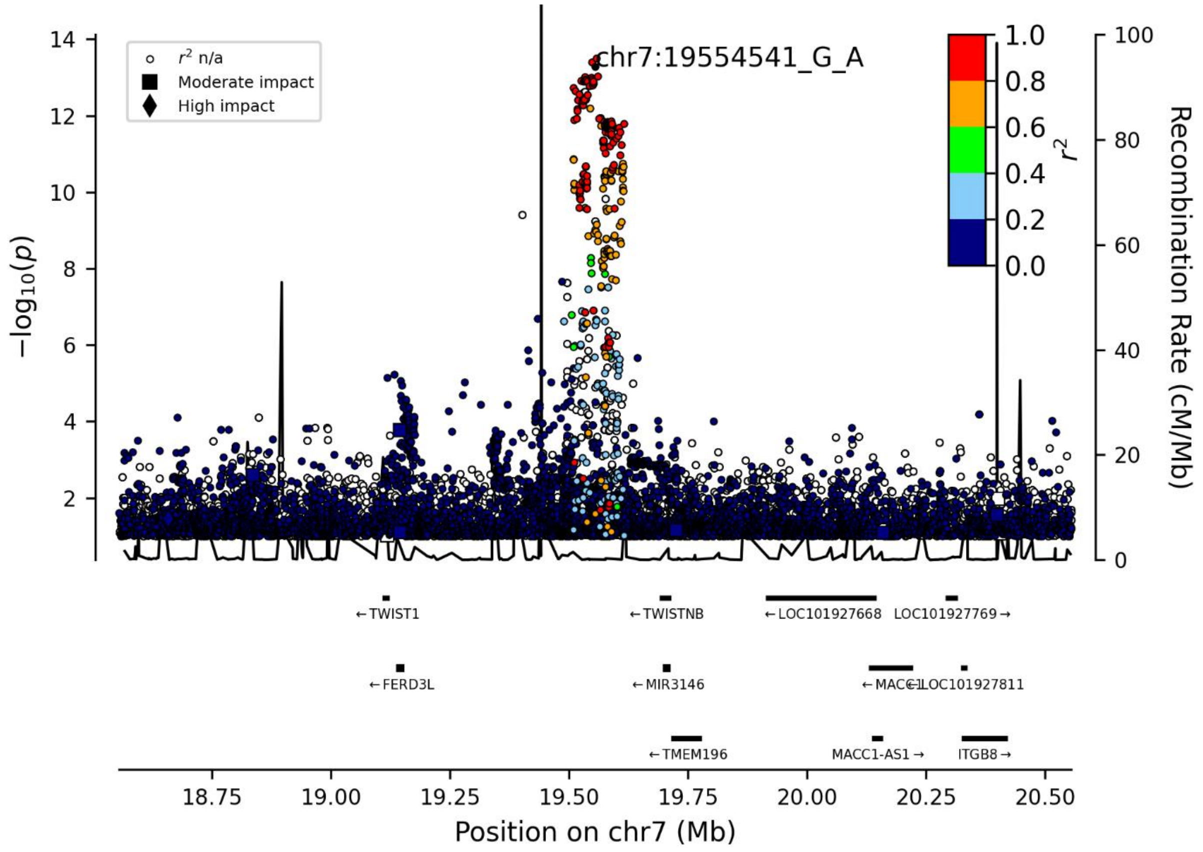

rs1723939

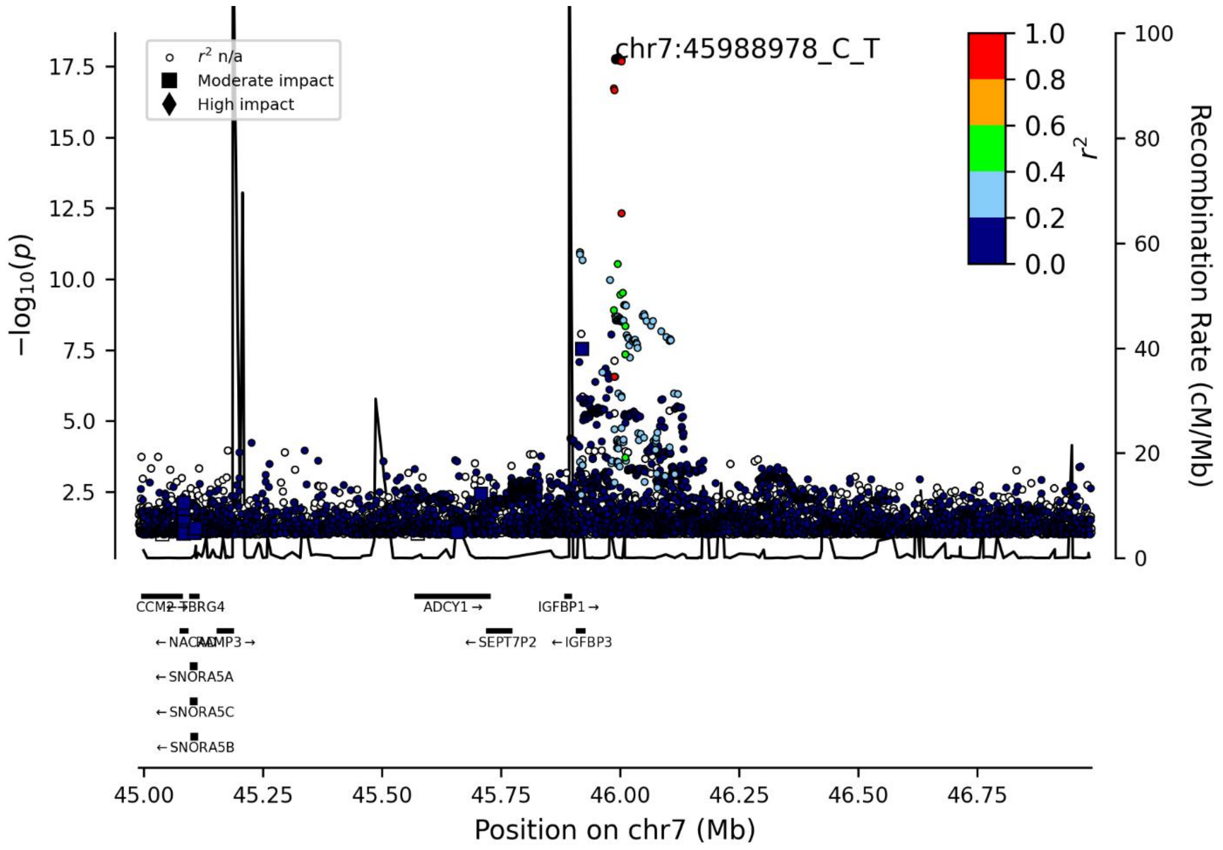

rs28364172

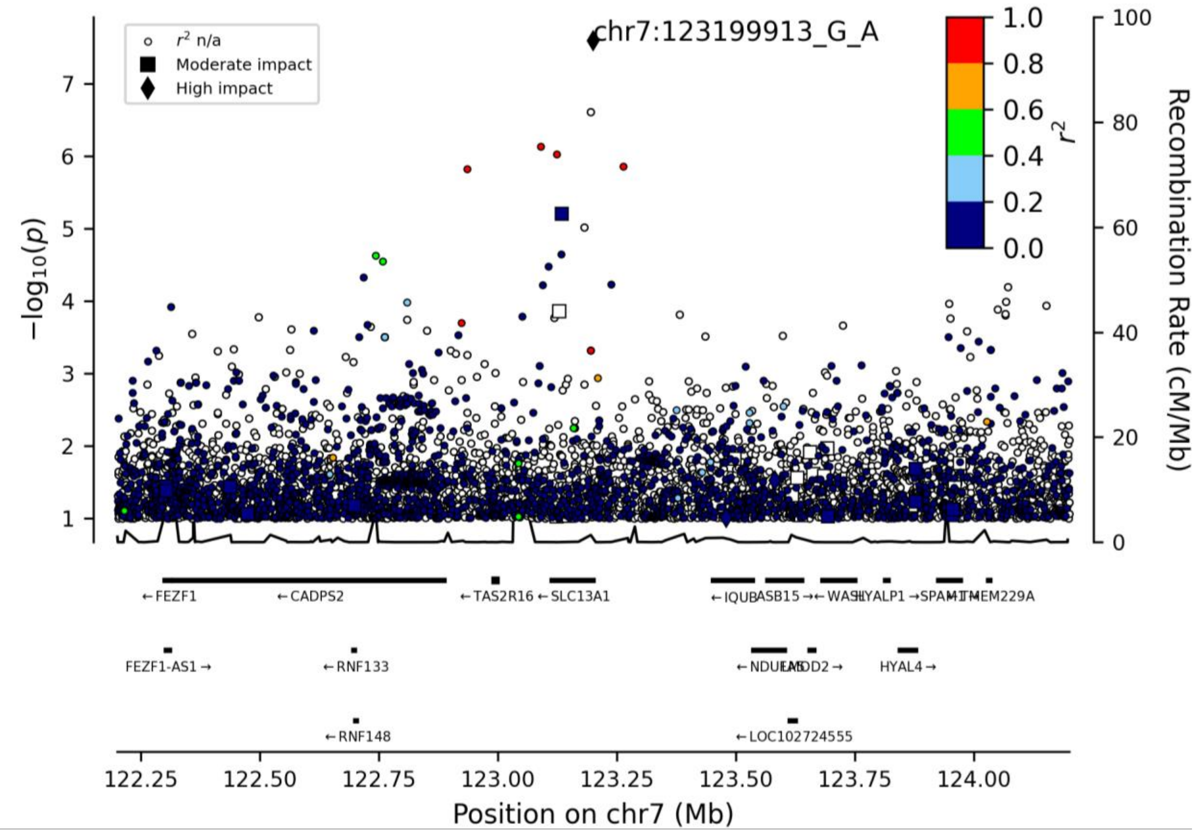

rs16934882

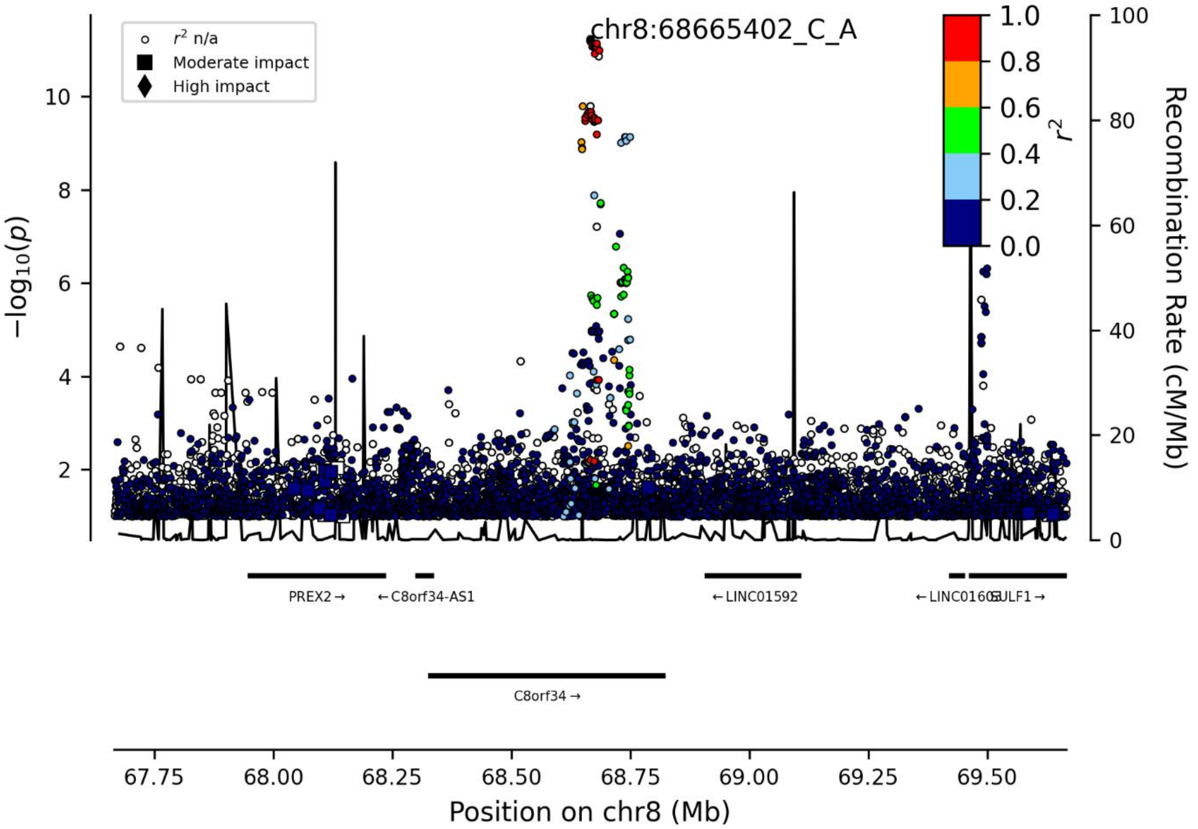

rs10110842

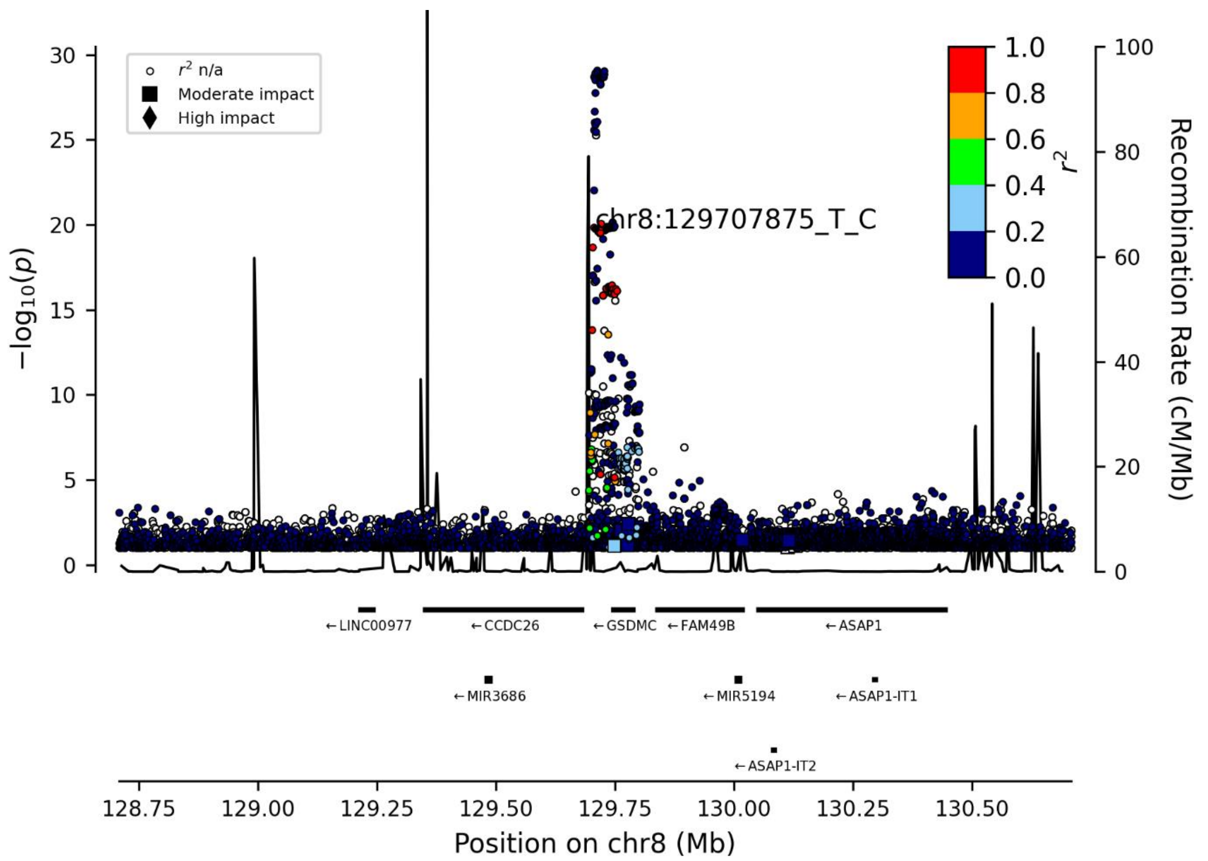

rs7826493

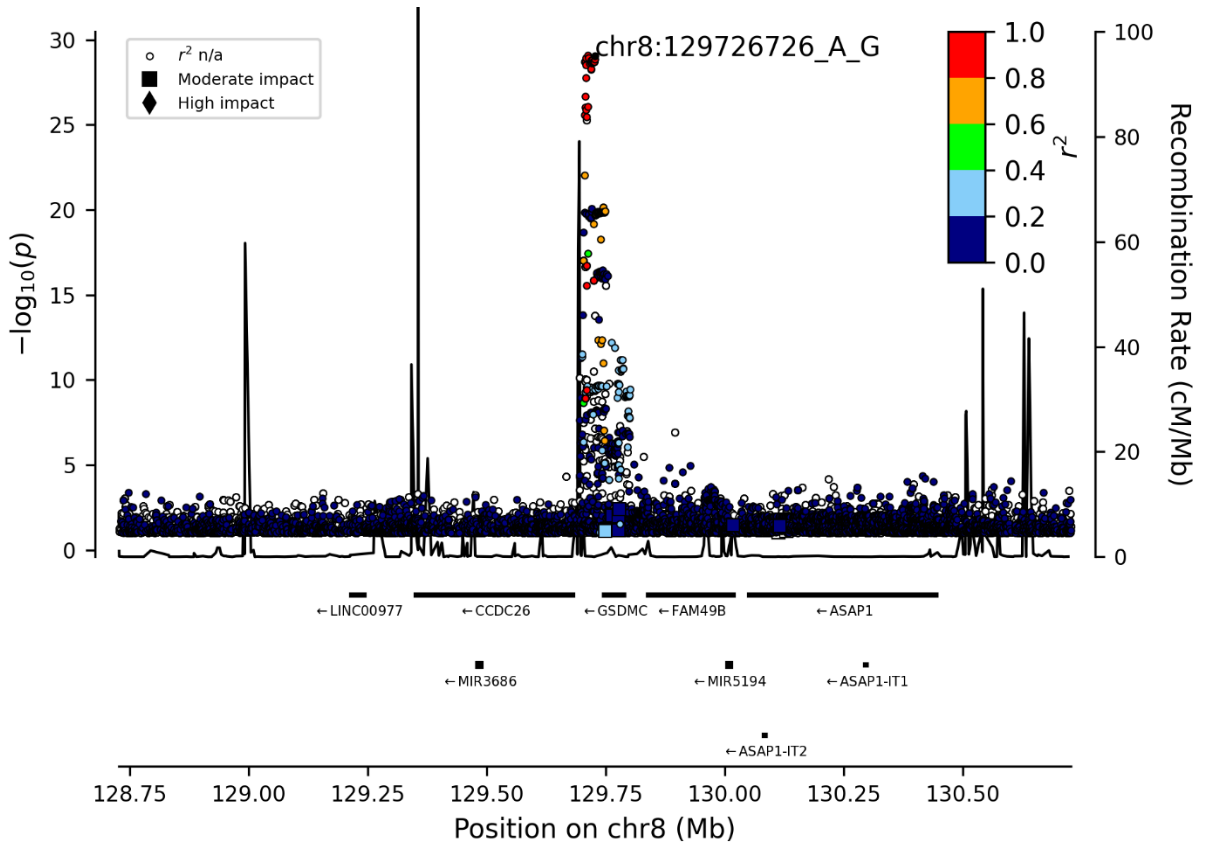

rs58723578

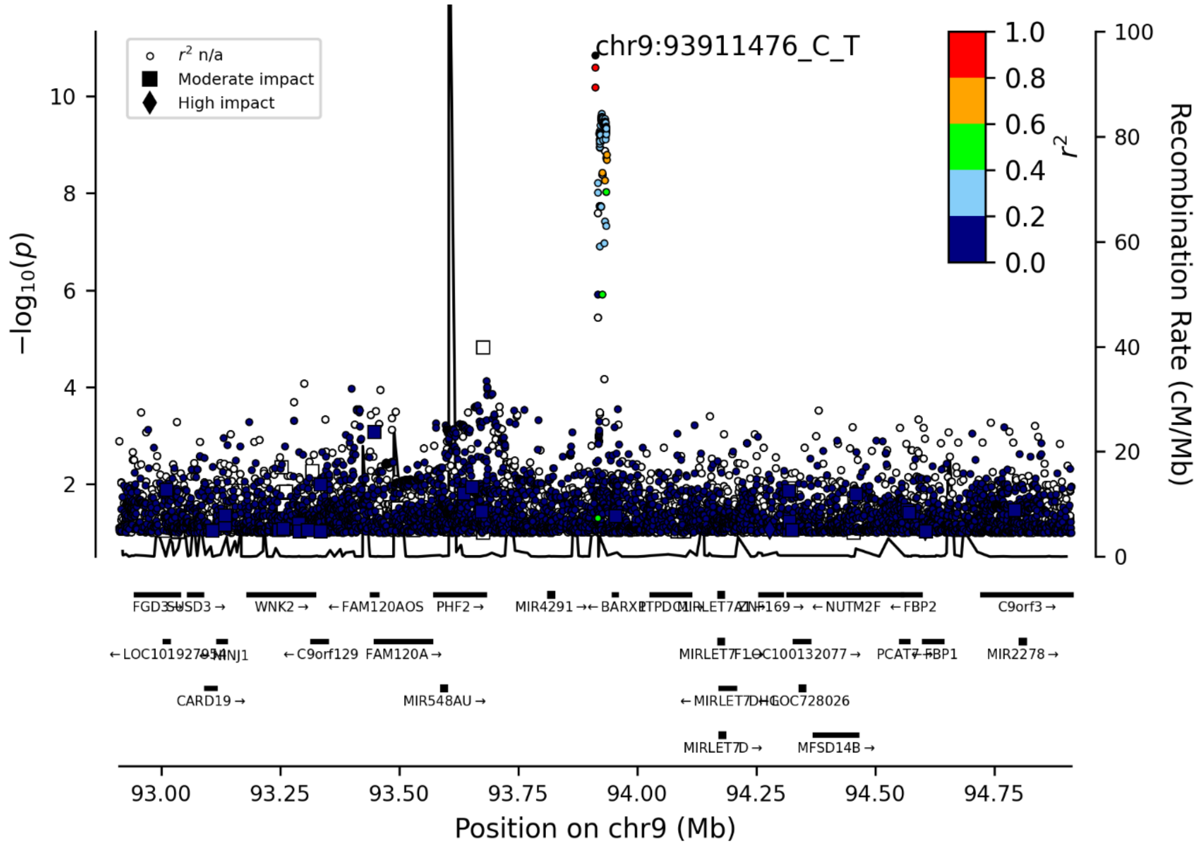

rs2637327

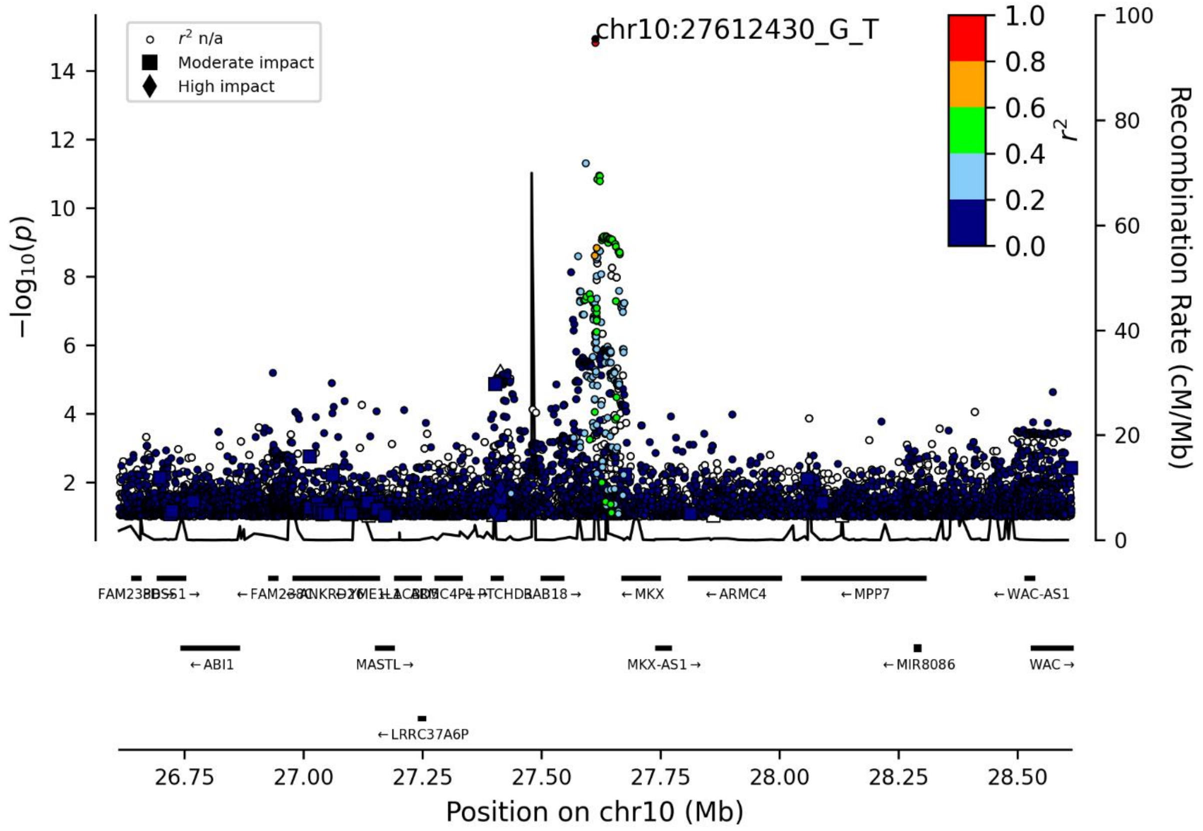

rs1871452

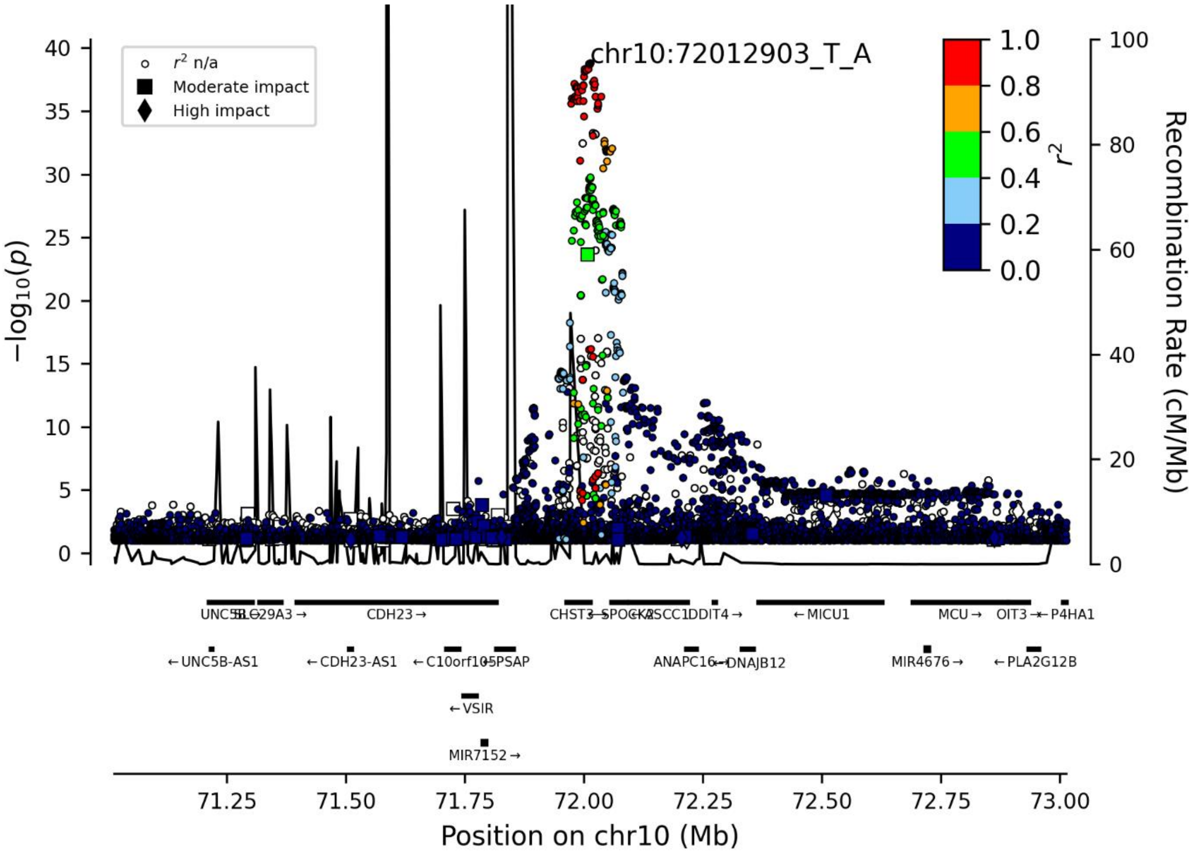

rs7098825

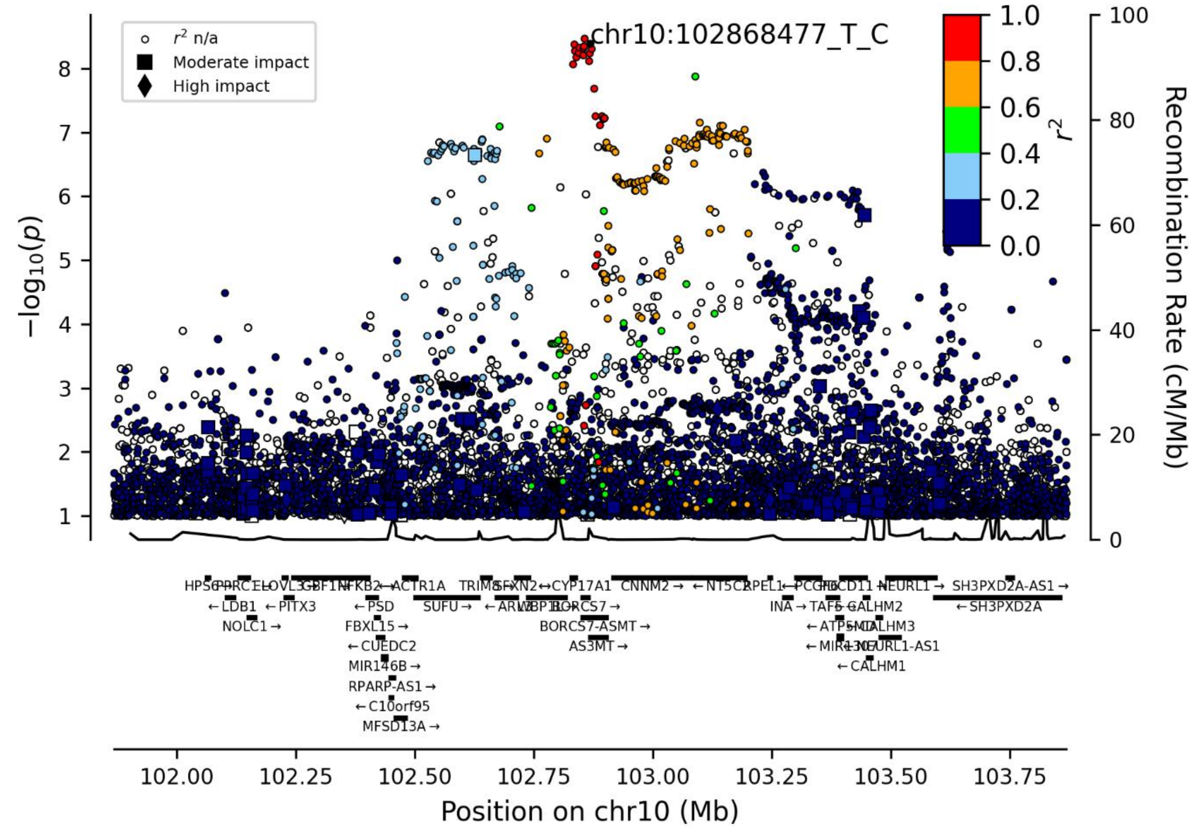

rs11022742

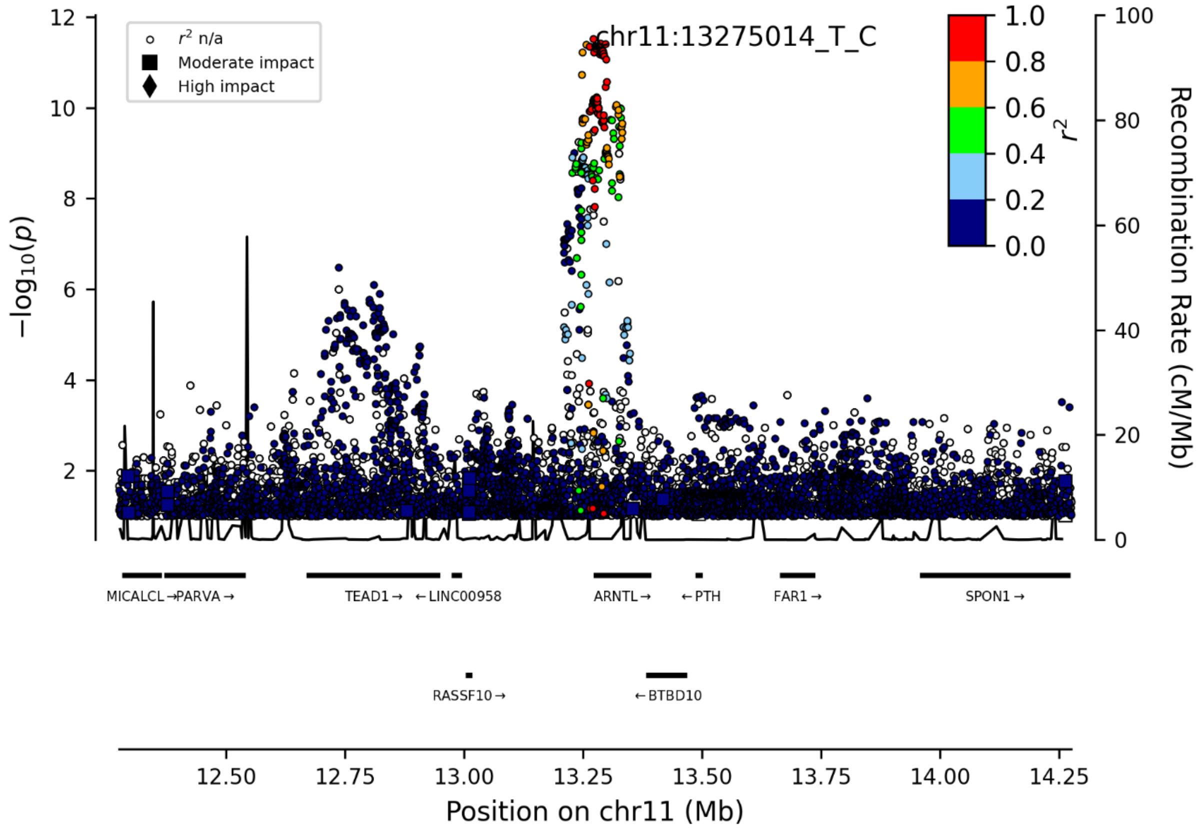

rs4757353

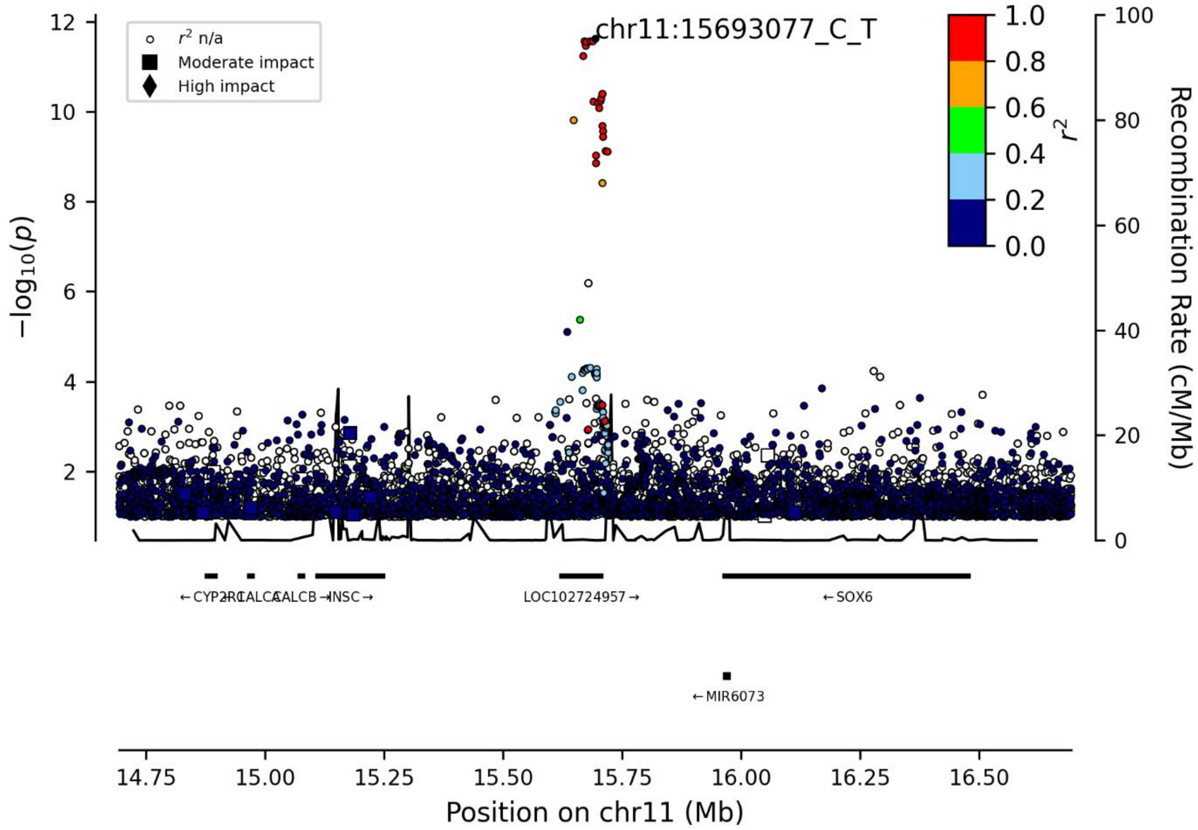

rs12310519

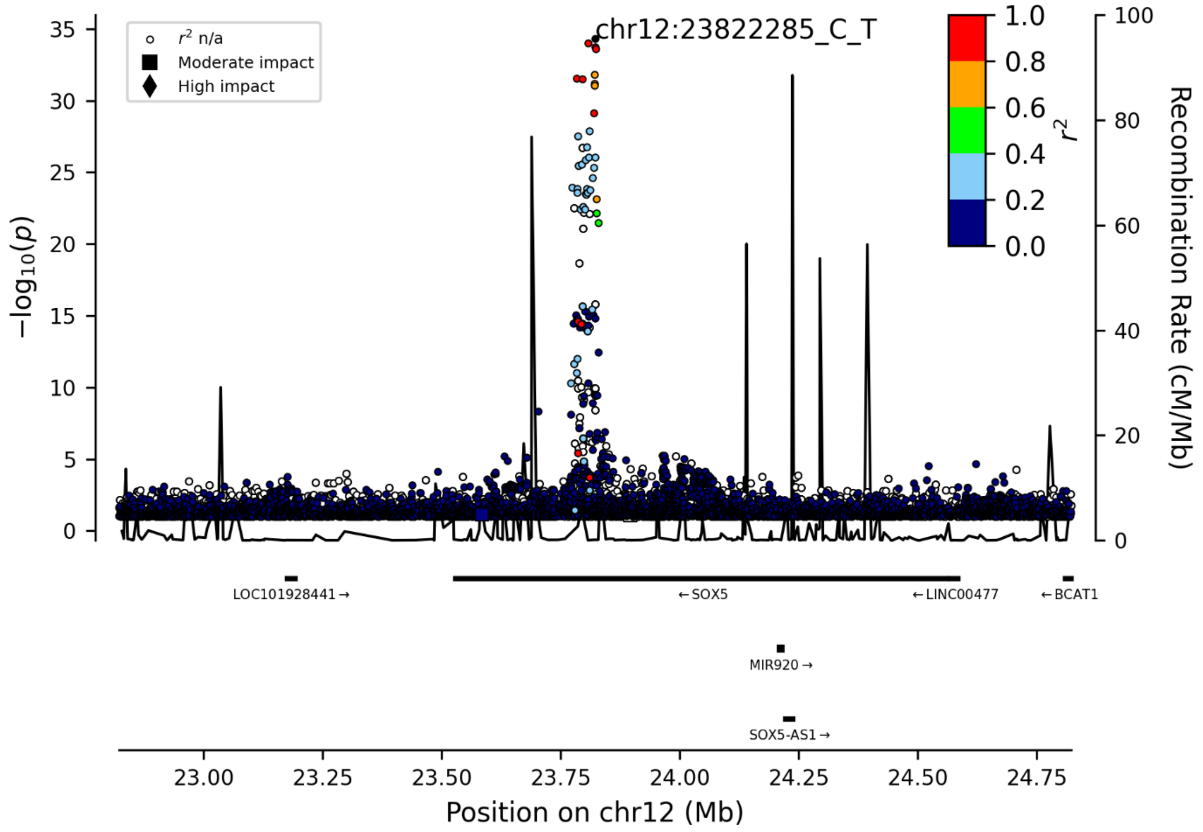

rs28487989

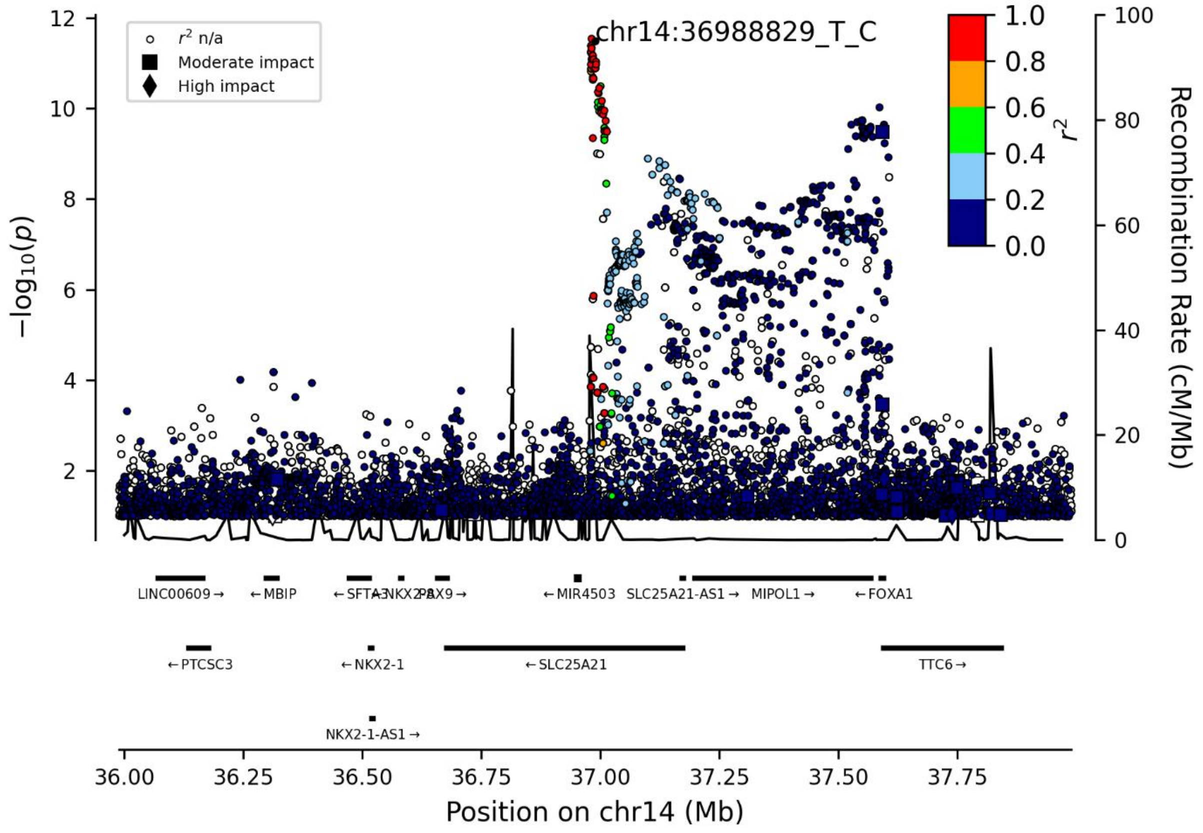

rs28929474

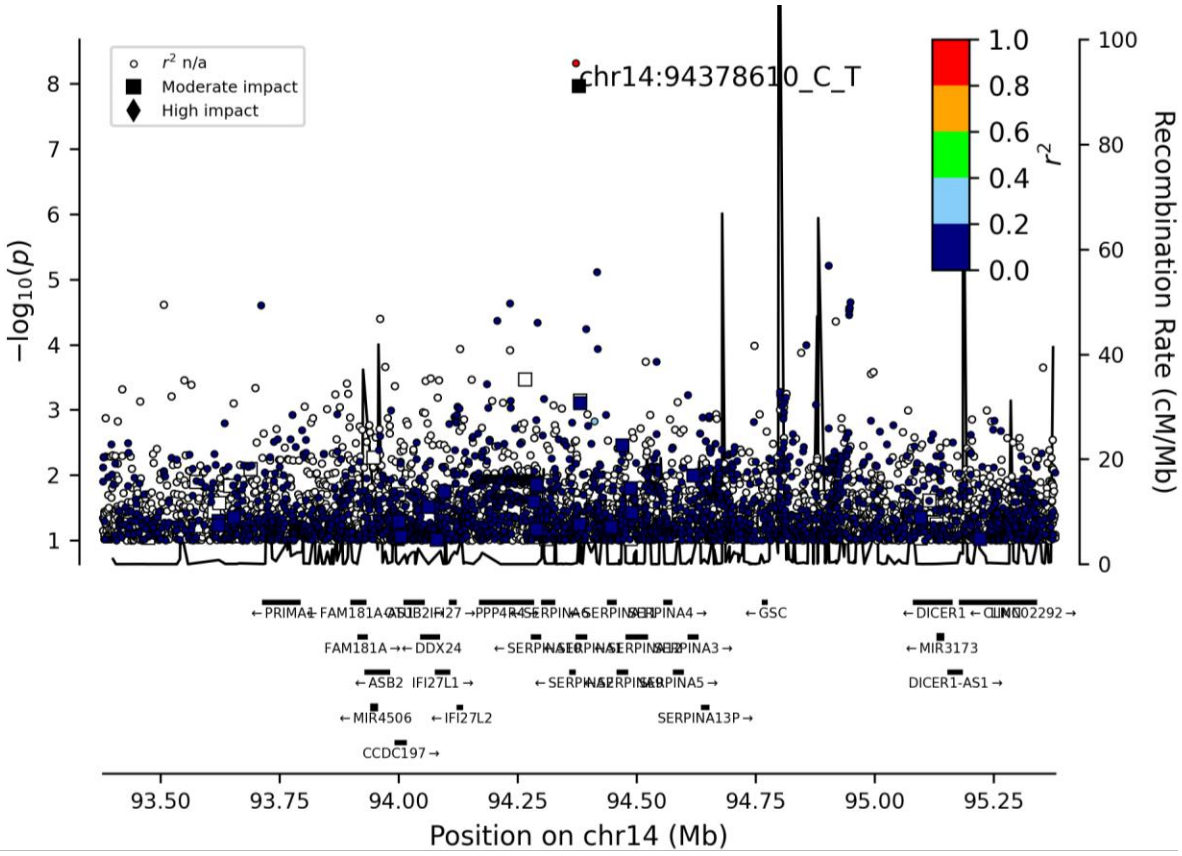

rs12901372

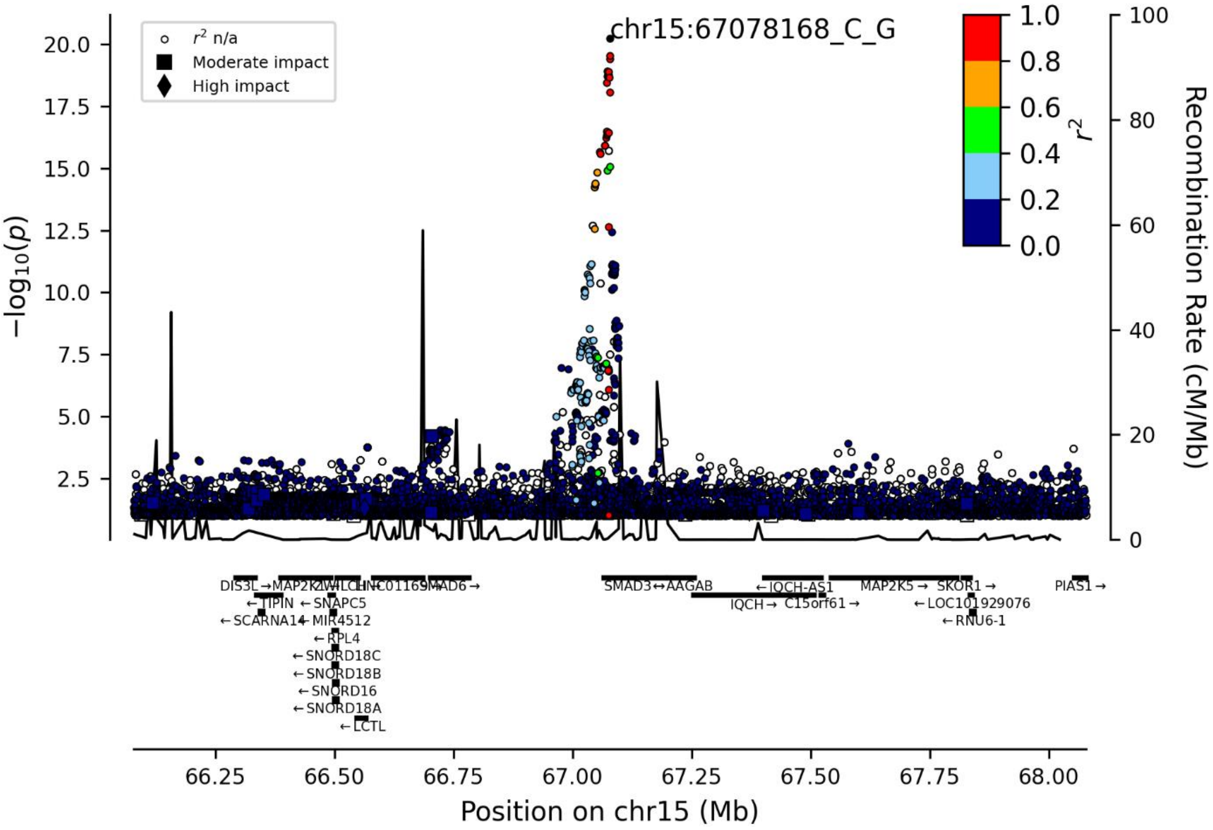

rs4776881

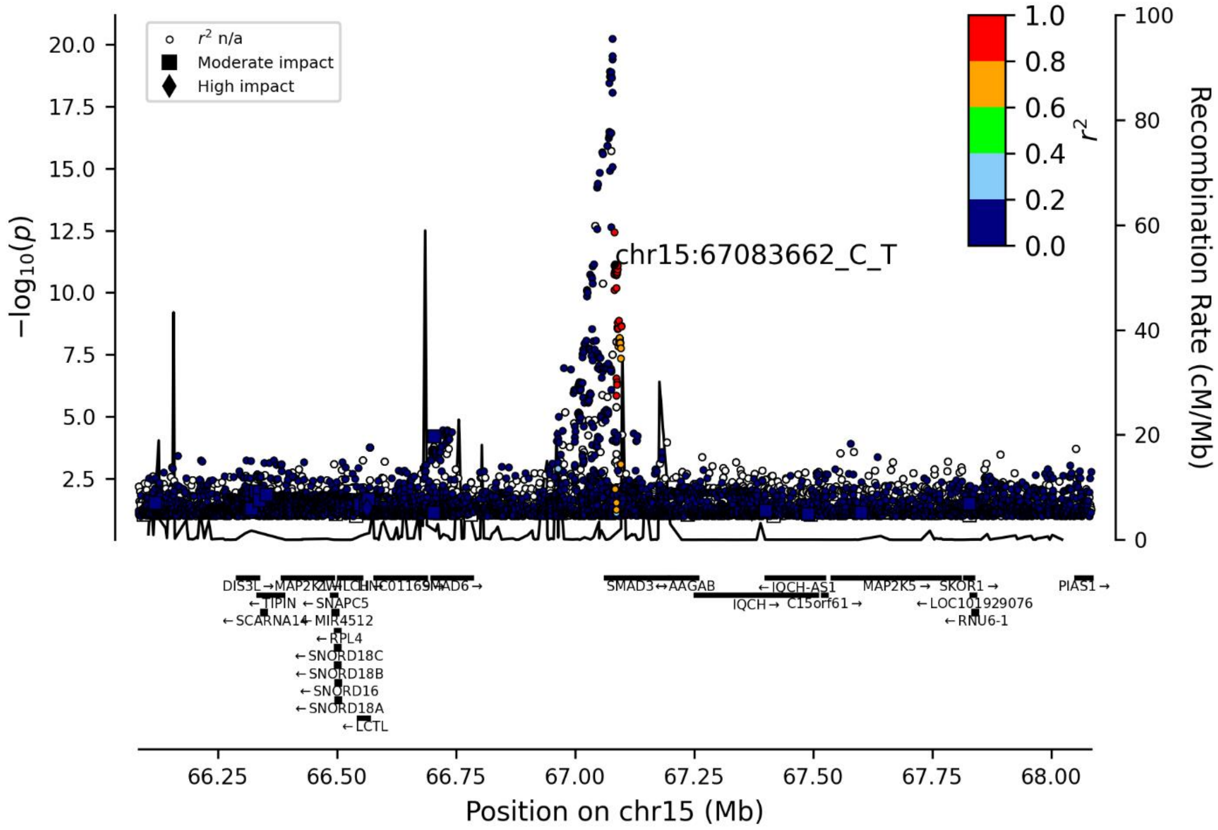

rs2040347

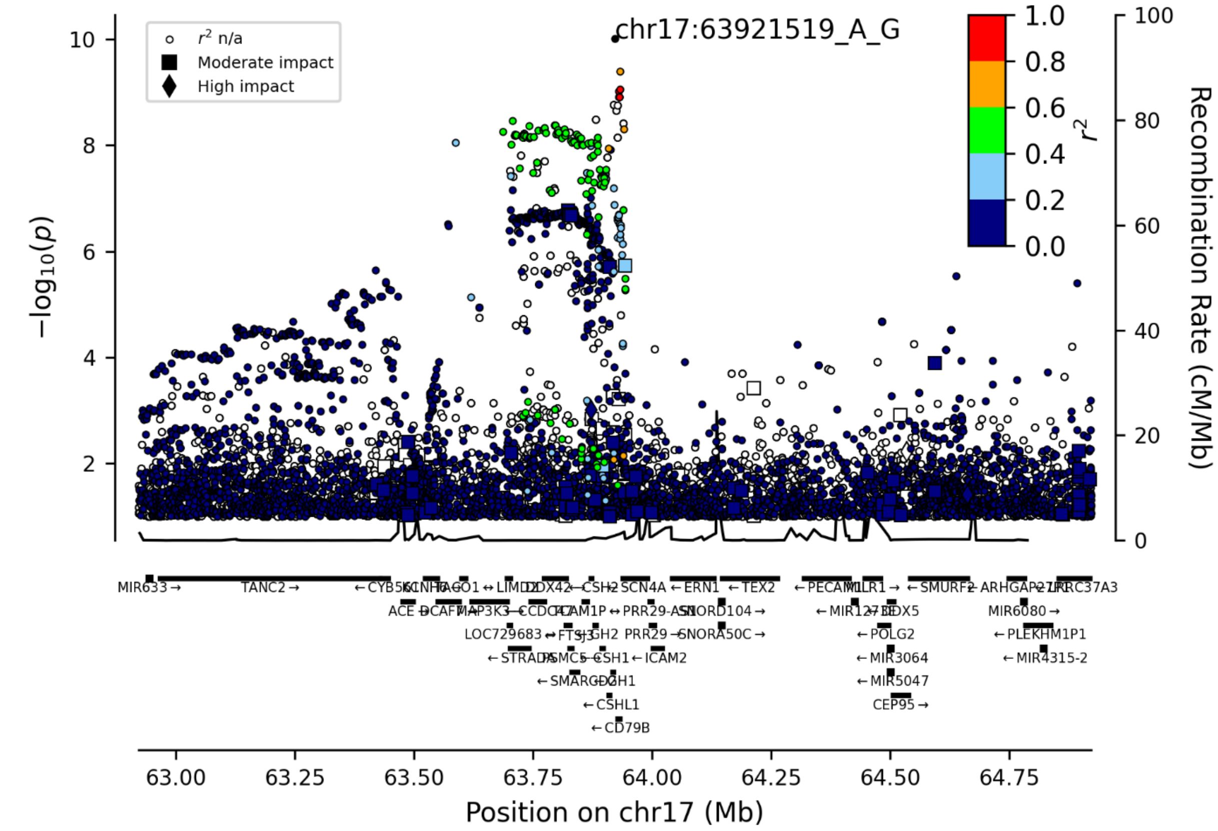

rs35318830

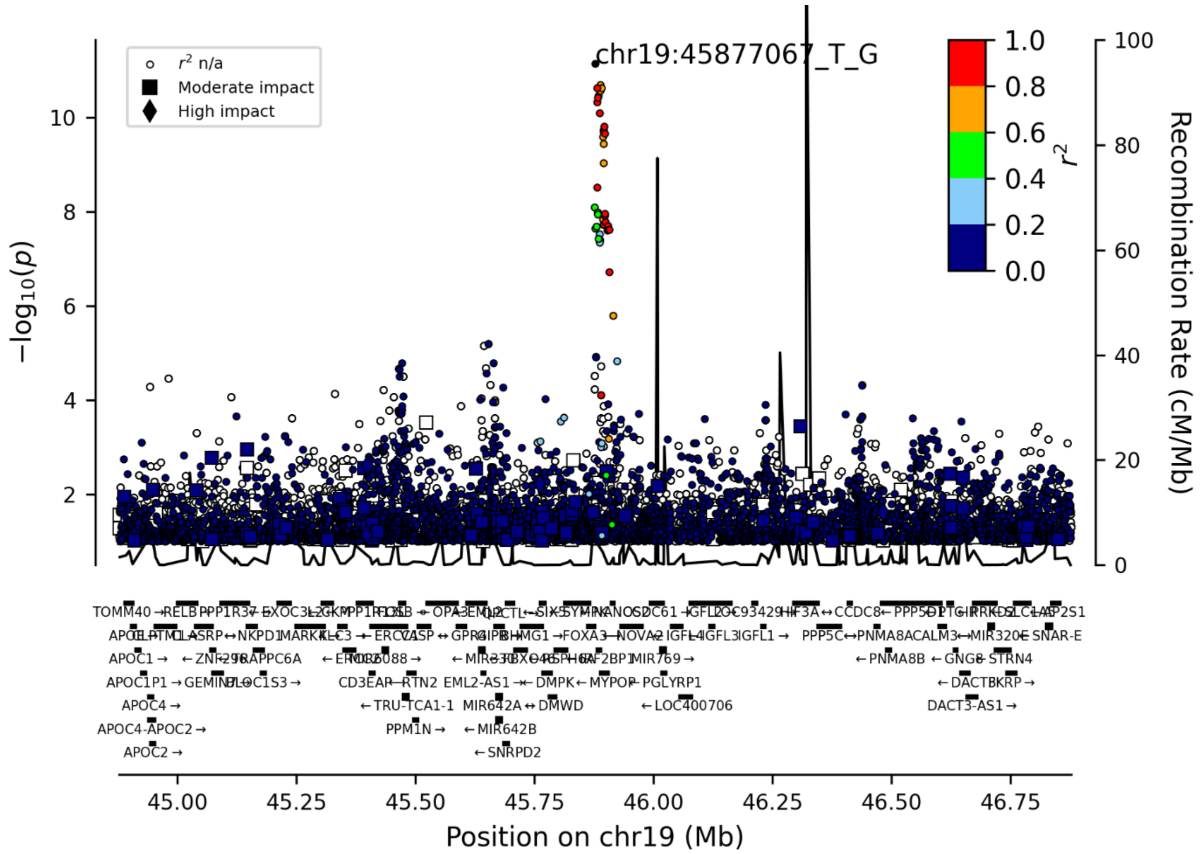

rs143384

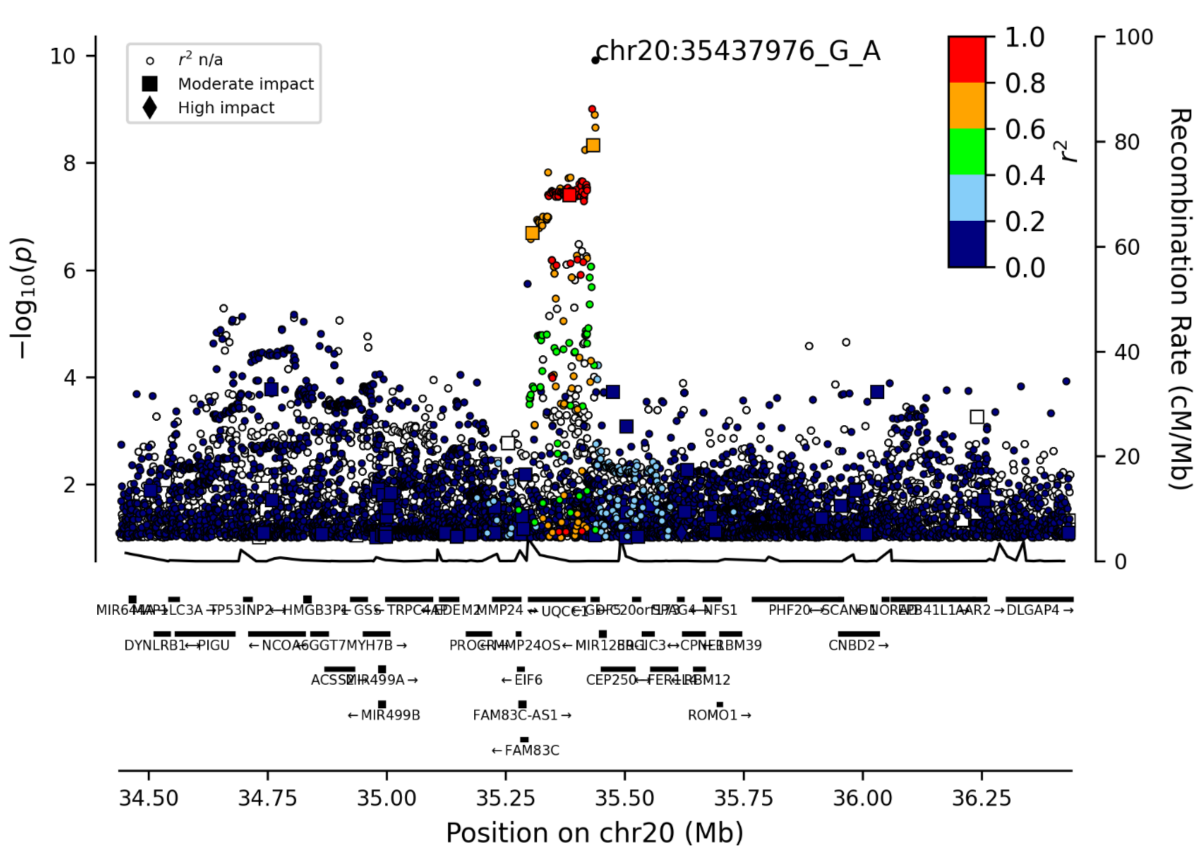

Figure 2. Locus plots for lead Dorsalgia variants

Following are regional visualization figures (Locus Plots) of the dorsalgia lead variants. The leading variant at each locus is colored in black. Other variants are colored by the degree of correlation (r^2) with the lead variant (rsname and chromosomal position hg38_other allele_effect allele). The − log10 *P*-values on the left y-axis (two-sided logistic regression) are plotted for each variant against their chromosomal position (x-axis). The right y-axis shows calculated recombination rates based on Icelandic data at the chromosomal location, plotted as solid black lines.

rs4907985

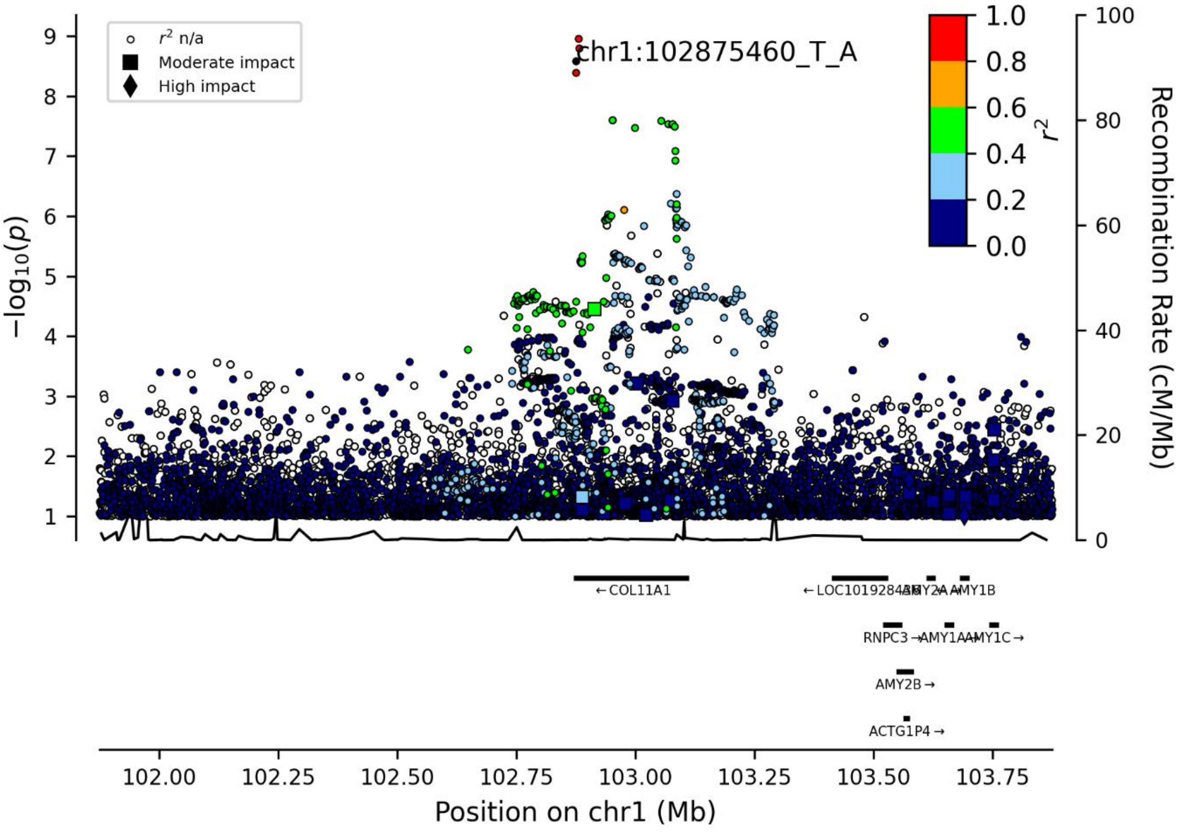

rs7560502

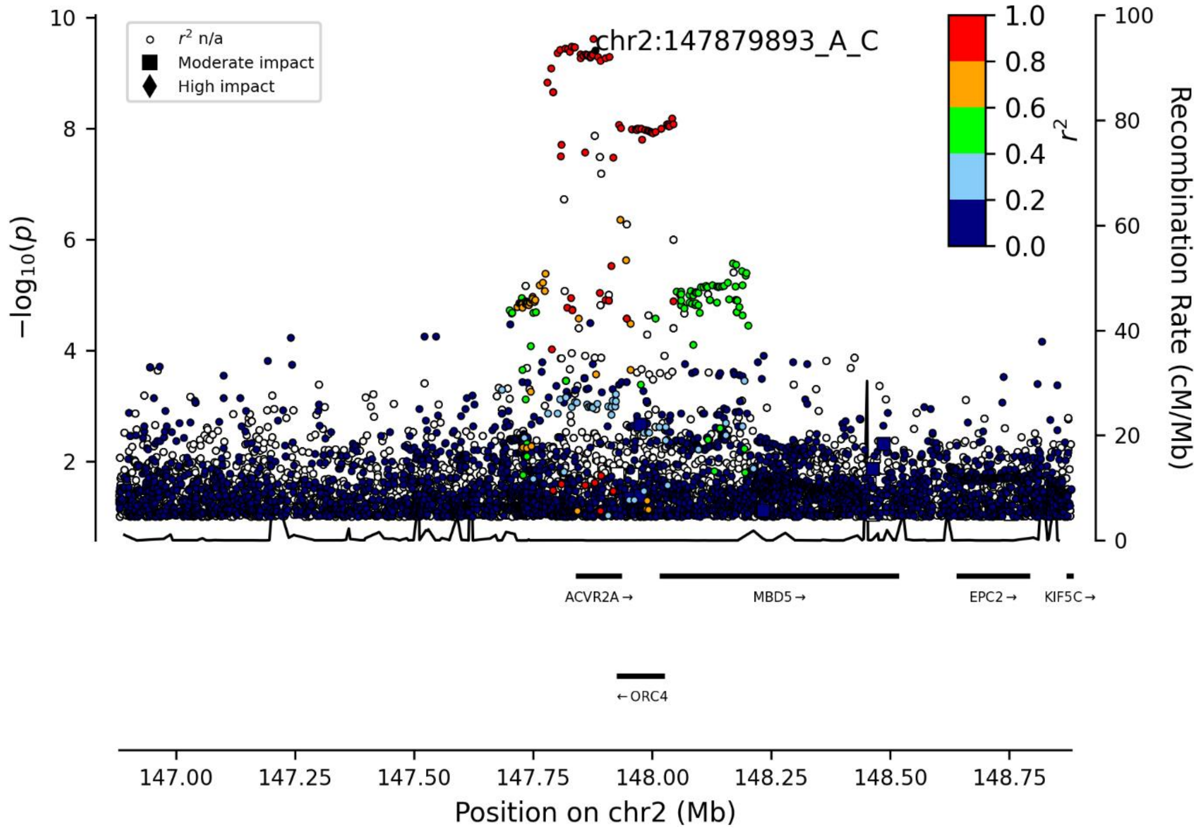

rs34762726

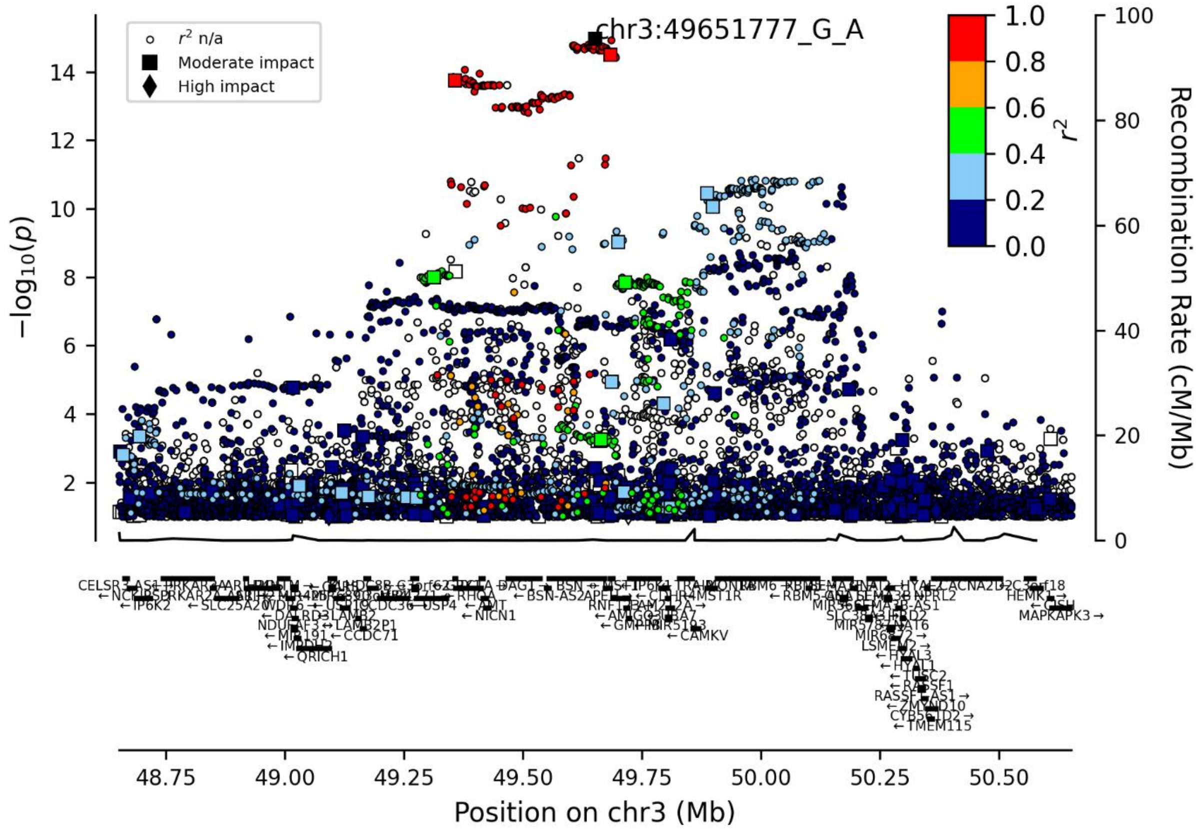

rs73090626

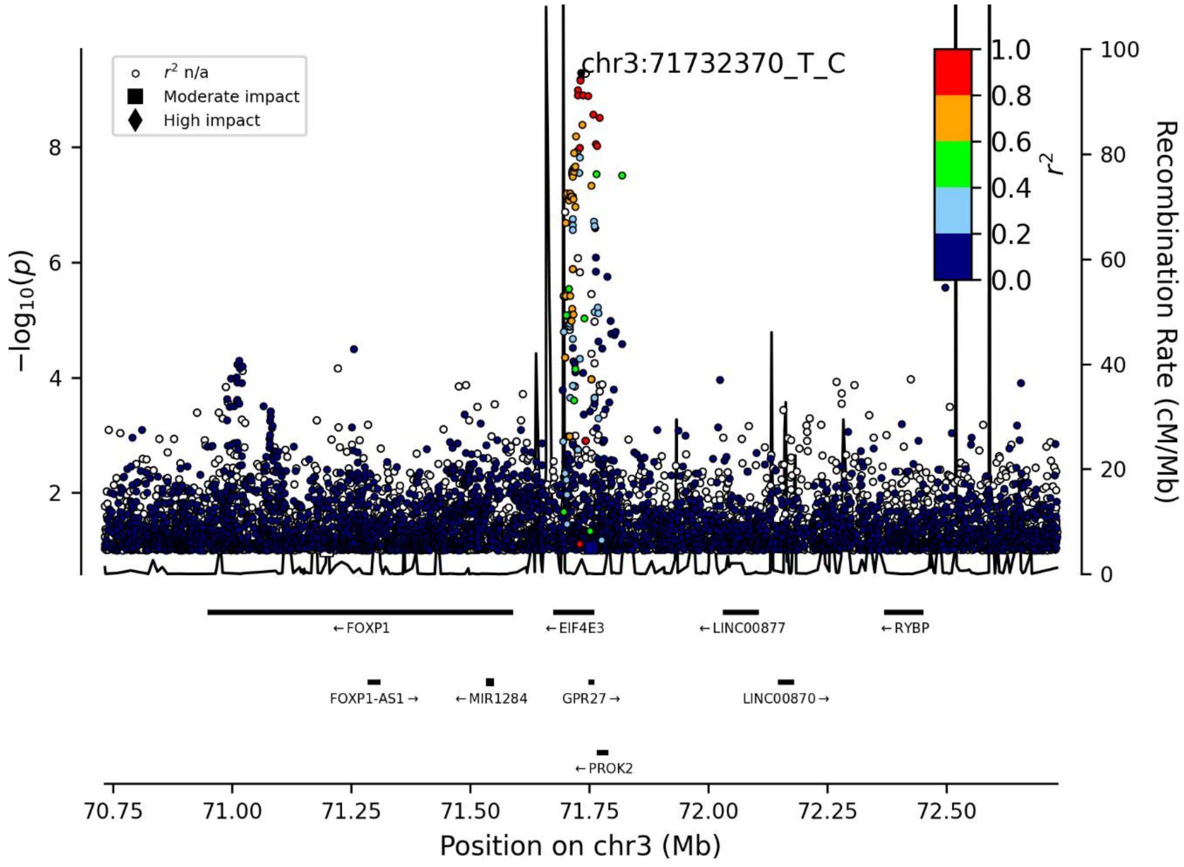

rs1995245

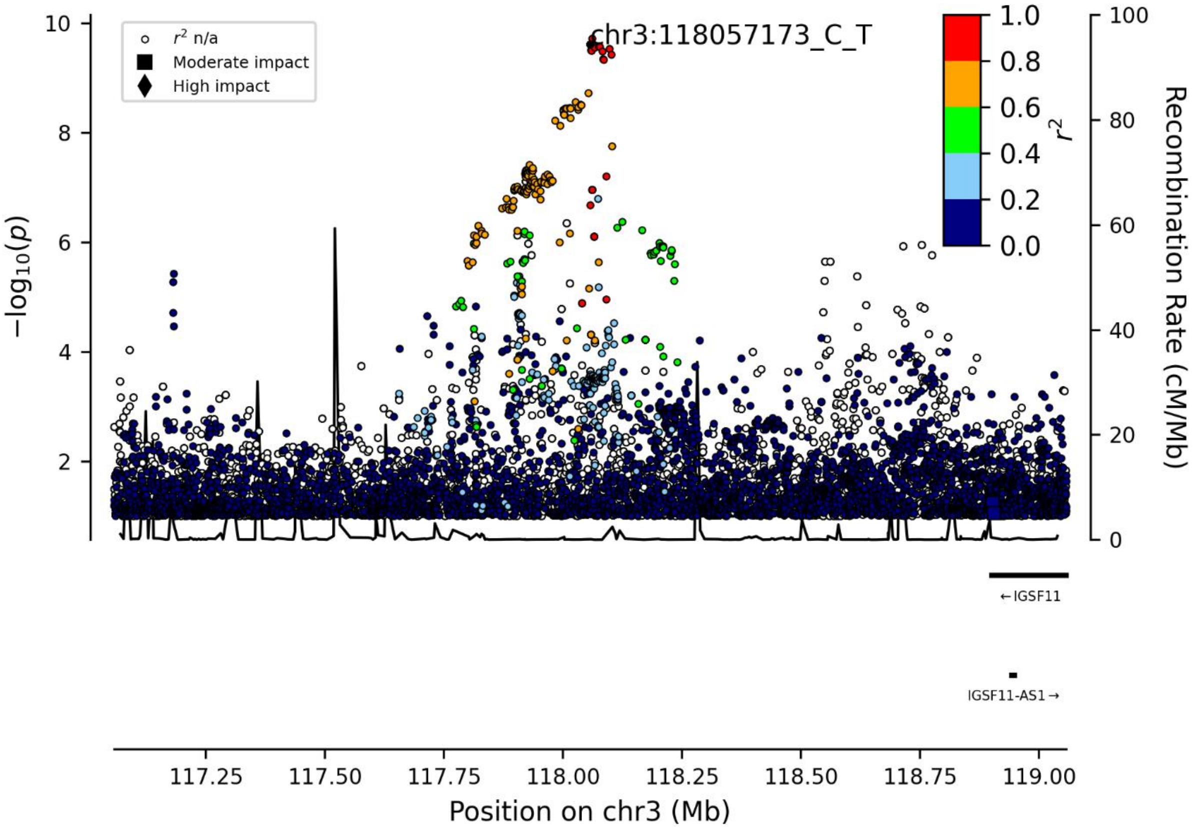

rs4865462

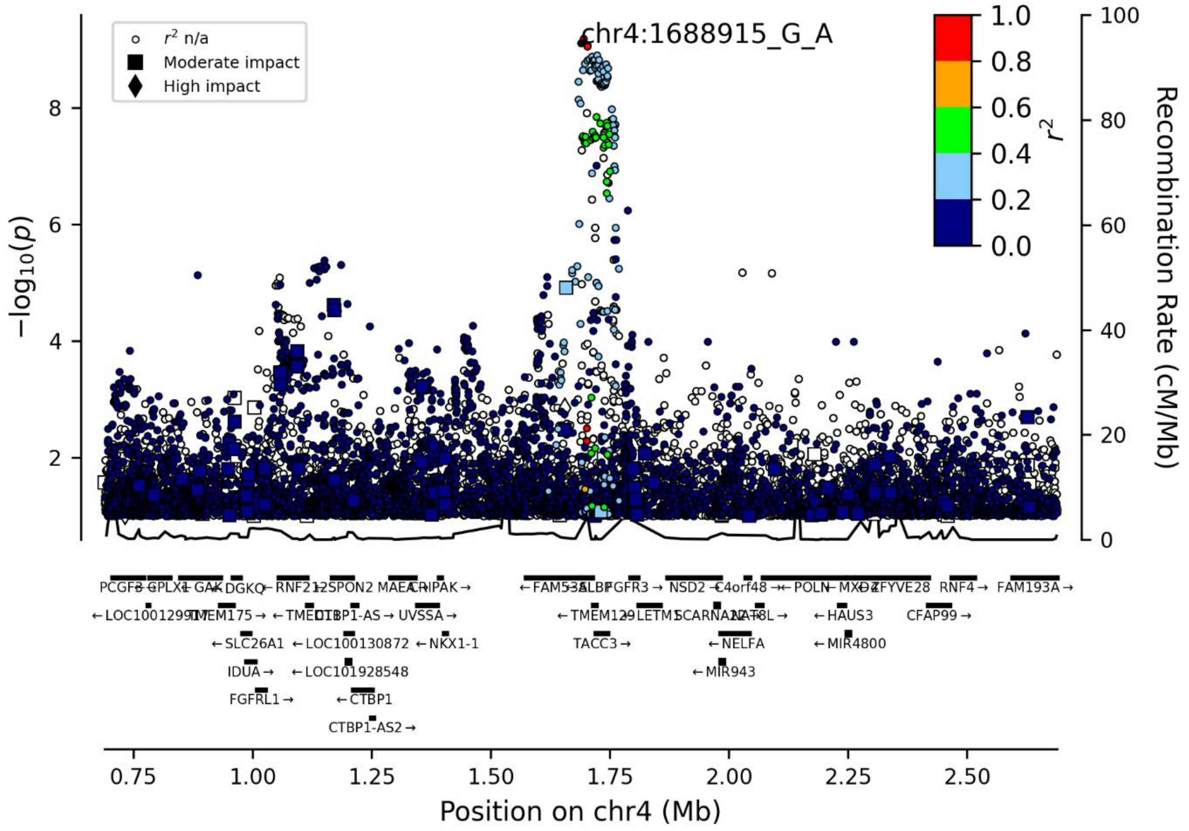

rs205262

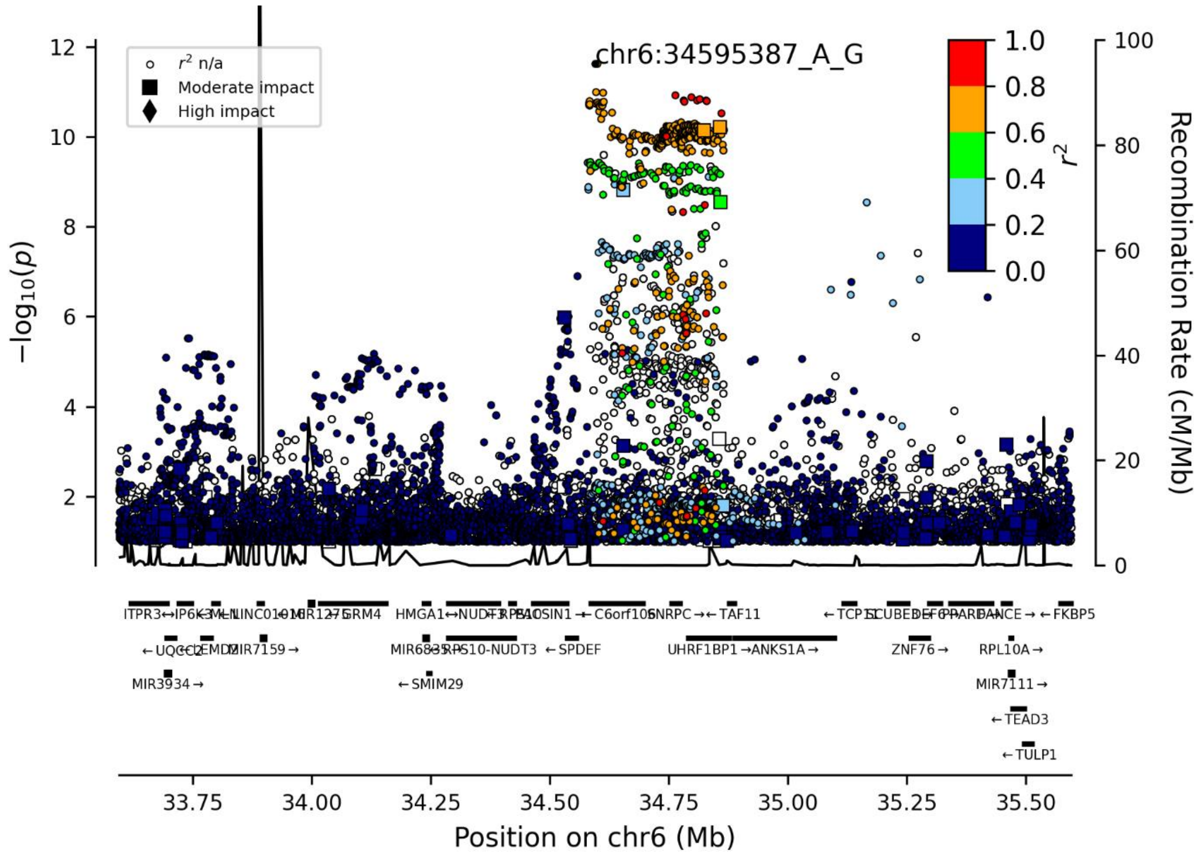

rs2272095

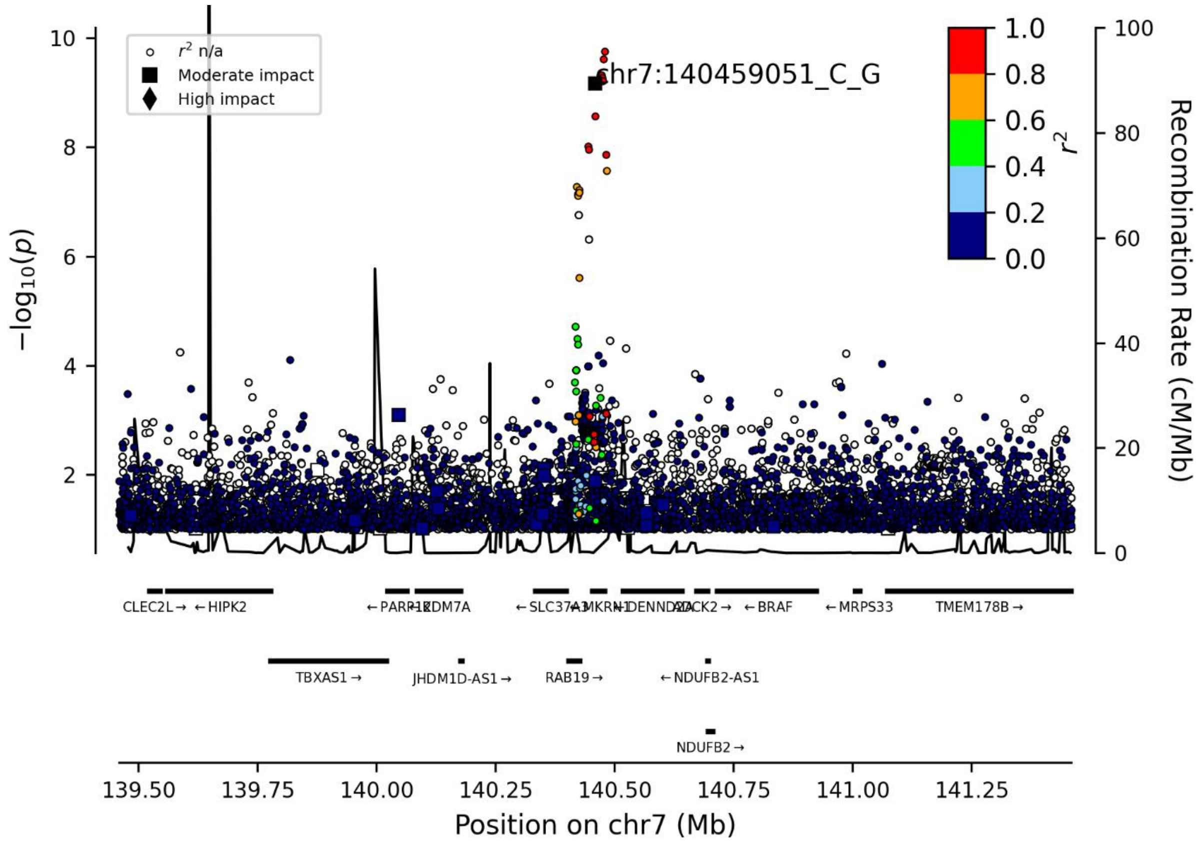

rs10110842

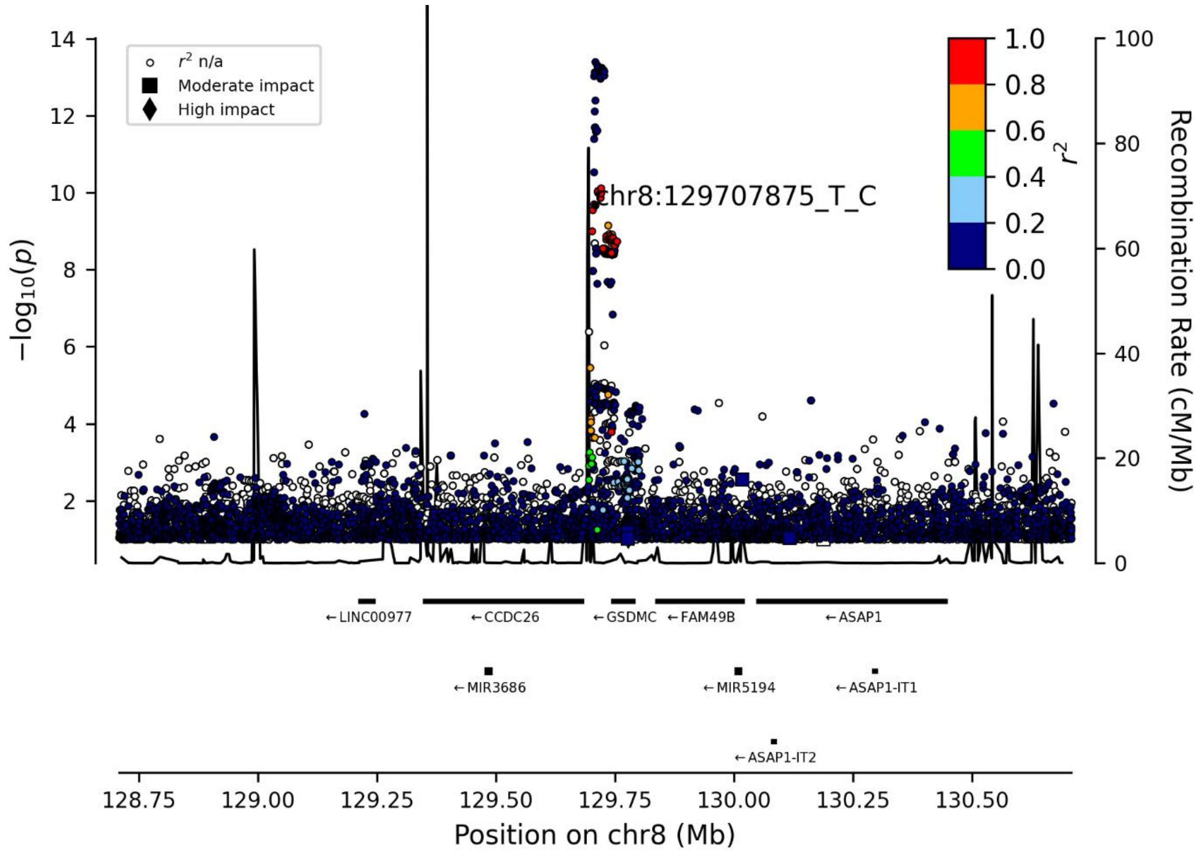

rs7826493

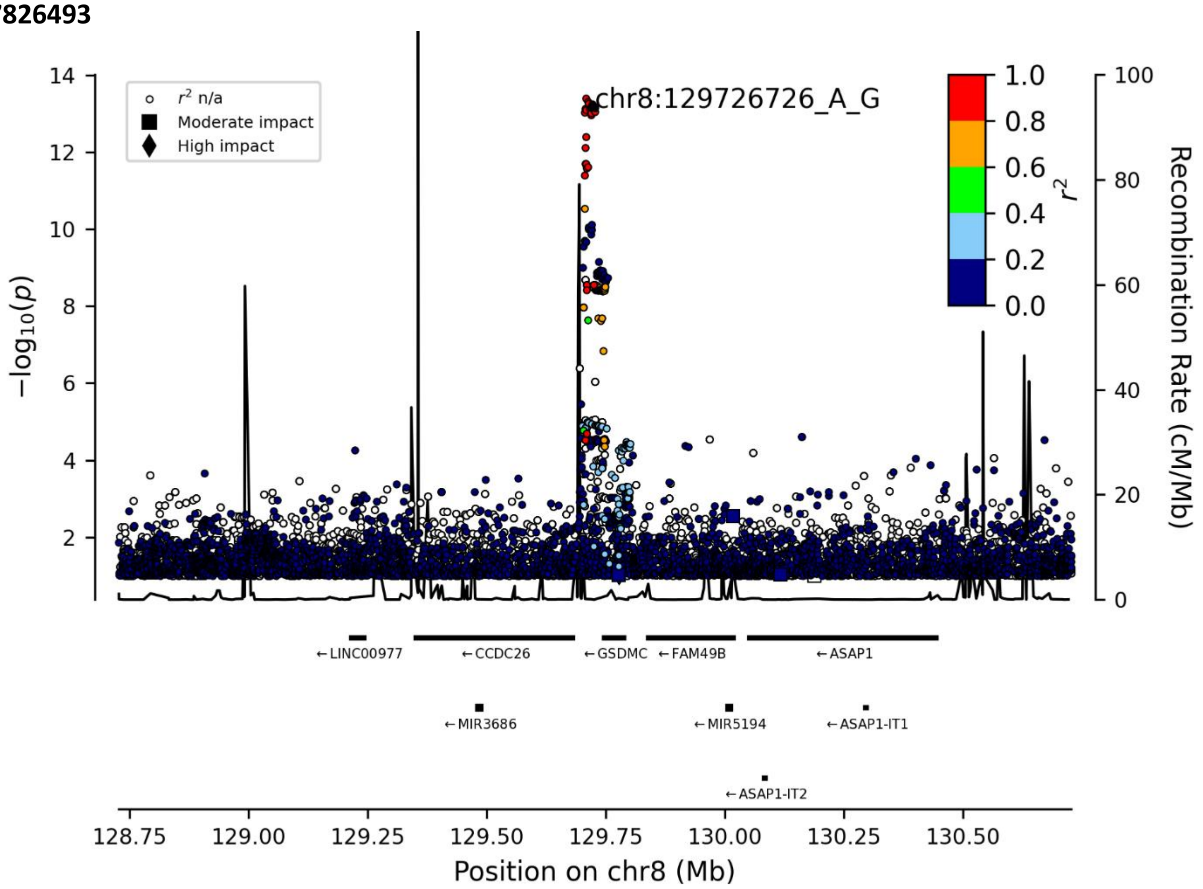

rs751450

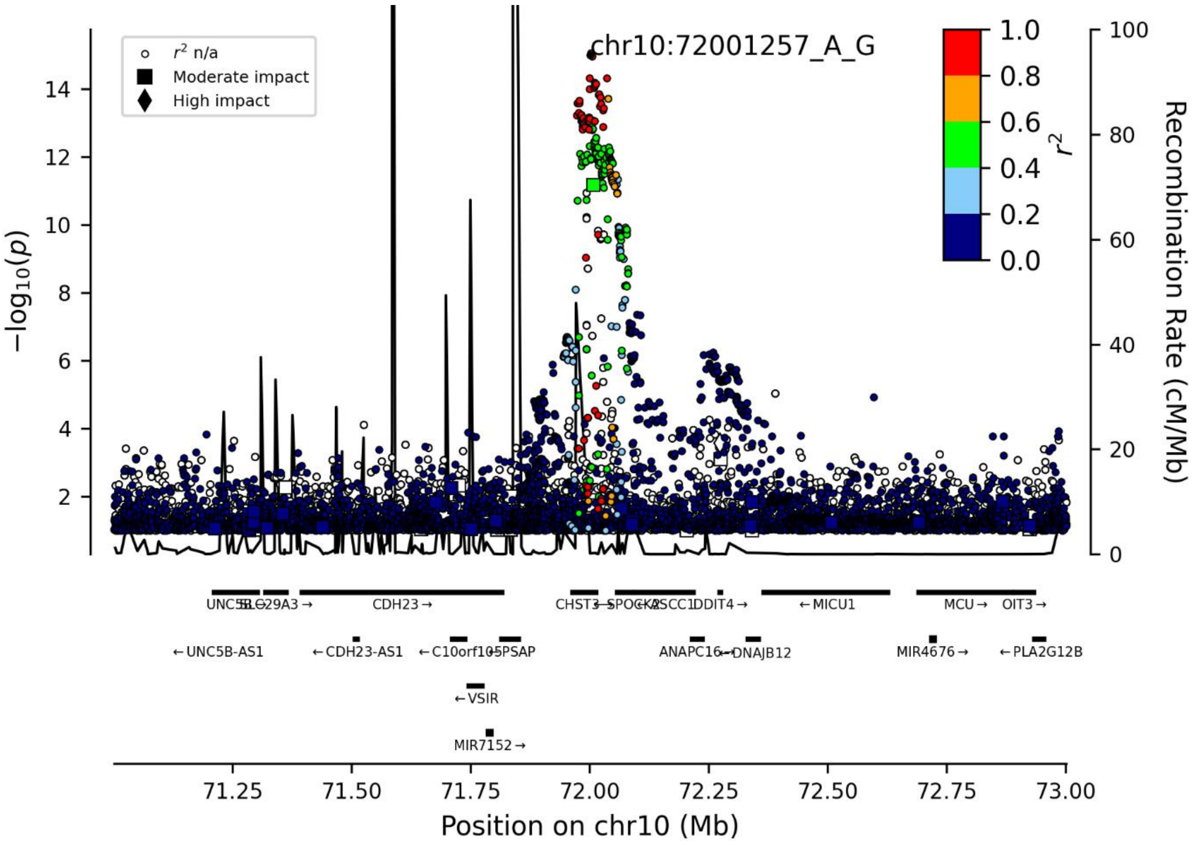

rs9953231

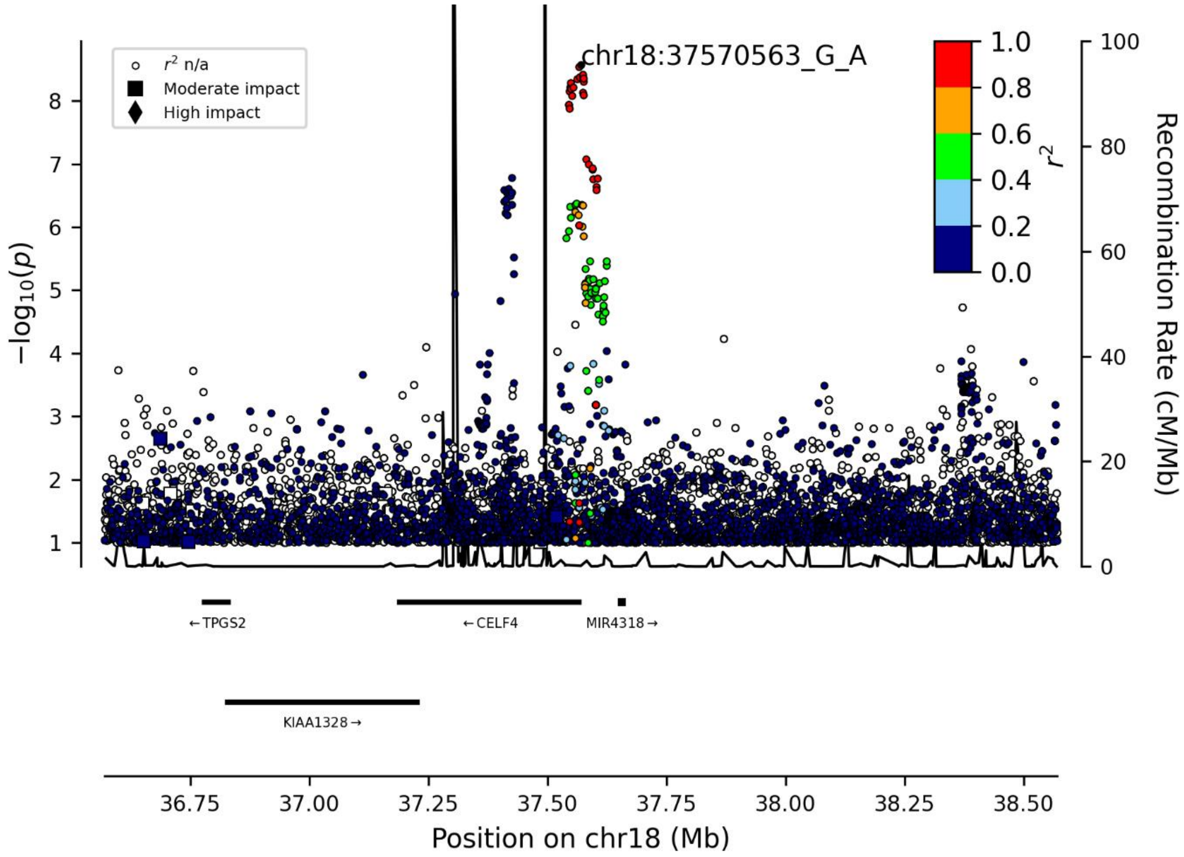

rs71338065

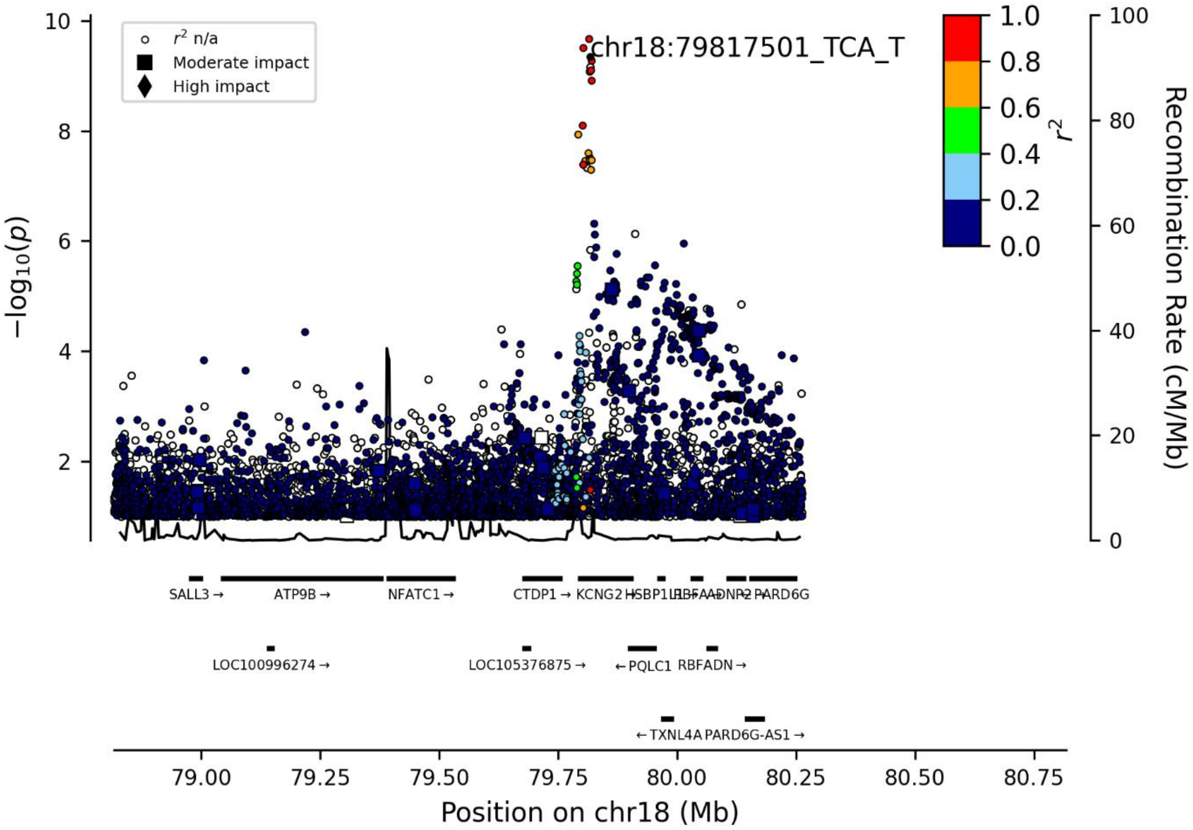

rs76838079

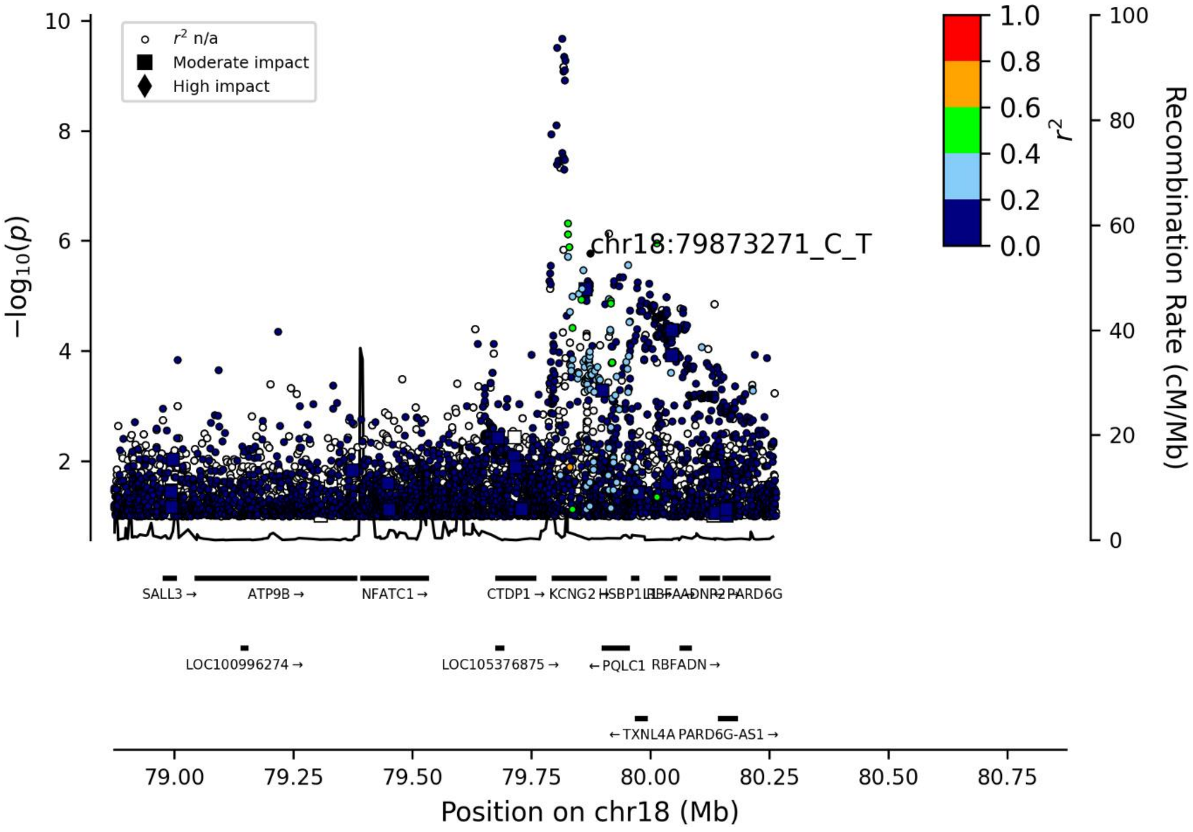

rs429358

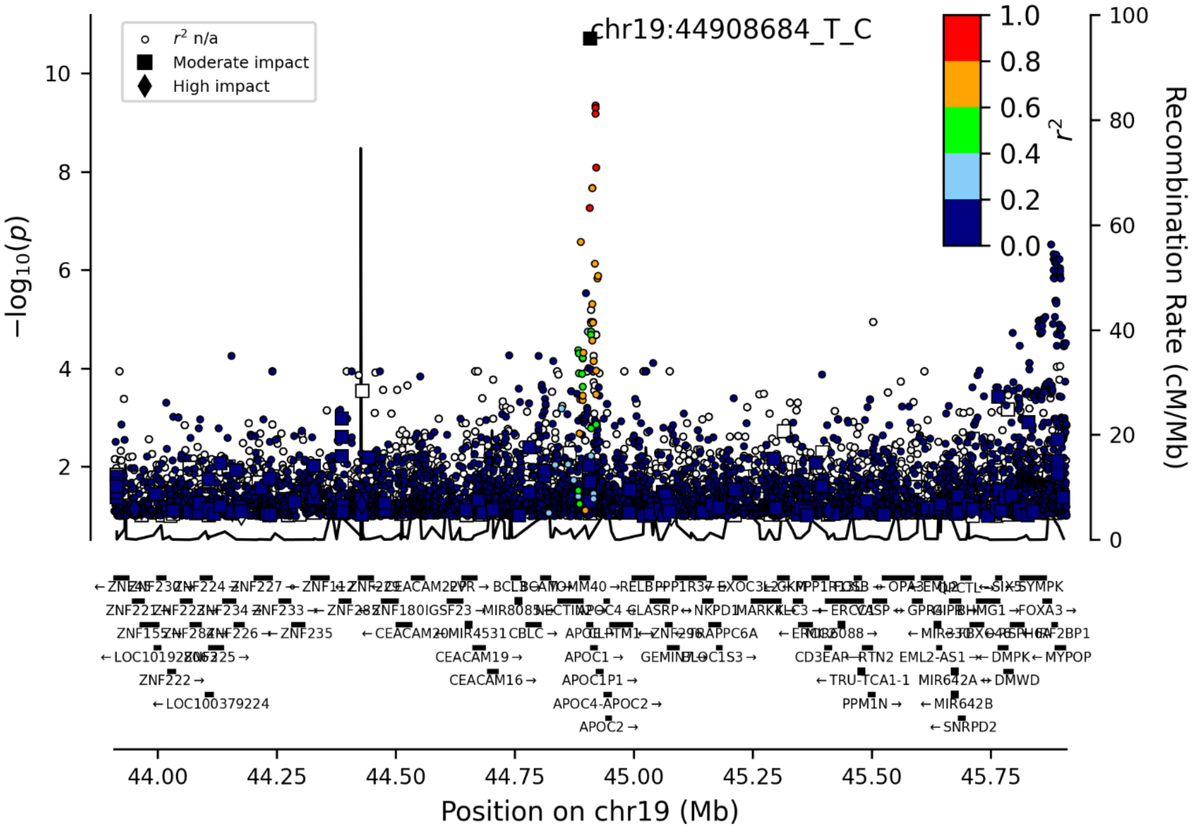

rs28536511

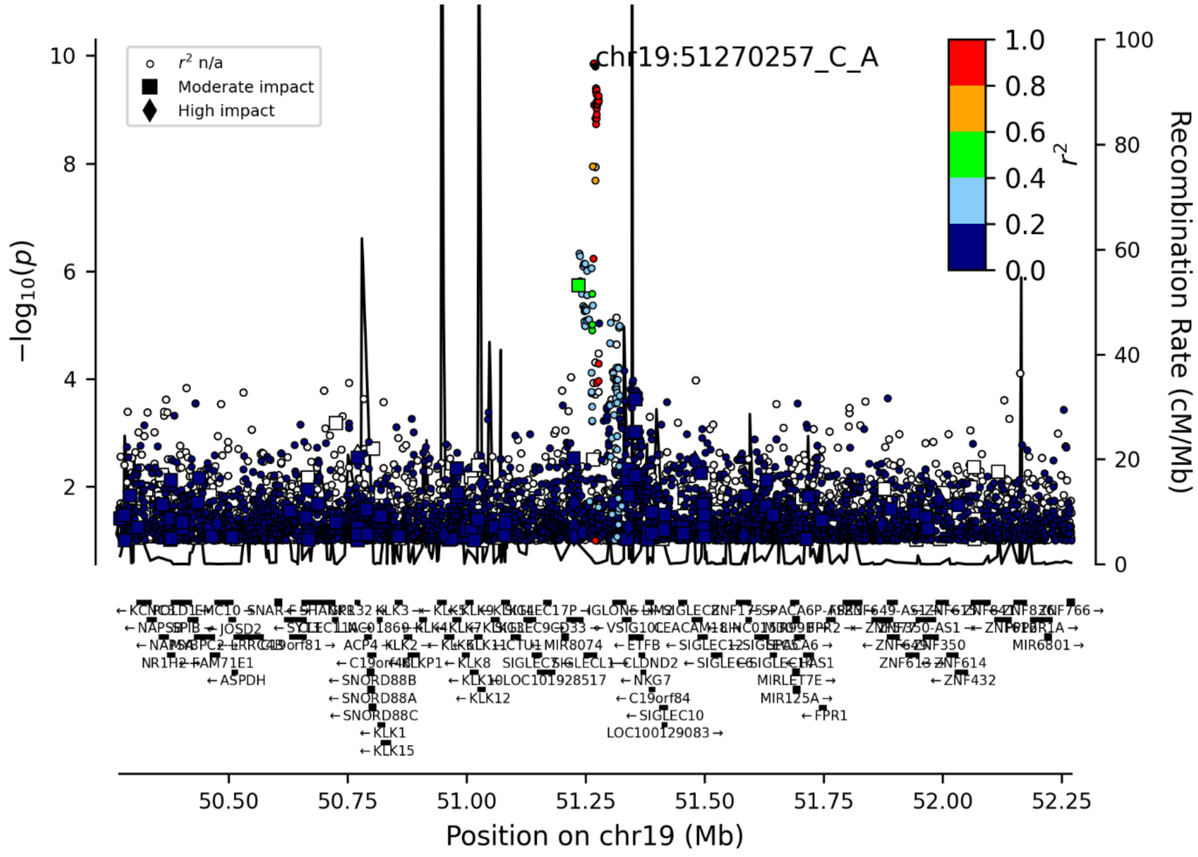

r^2 n/a
Moderate impact
High impact
chr19:51270257_C_A
$-\log_{10}(p)$
r^2
Recombination Rate (cM/Mb)
Position on chr19 (Mb)
50.50
50.75
51.00
51.25
51.50
51.75
52.00
52.25

Figure 3. Mendelian Randomization Sensitivity Analysis

We used the Heidi outlier removal approach, which is used in the Generalised Summary-data-based Mendelian Randomisation (GSMR) method[3]. We used the implementation from their published R package v1.0.9 (https://cnsgenomics.com/software/gsmr/) and used the default parameters. The method removed no outliers for IDD as exposure, but the method removed two outliers for Dorsalgia as an exposure (empty circles in figure below). The variants suggested for removal because of pleiotropy are rs751450 (chr10:72001257 *CHST3* intron variant) and rs71338065 (chr18:79817501 *KCNG2* intergenic variant), see figure below compared to Figure 3b in main text. We re-ran our analysis using this subset of markers and results are not fundamentally changed as demonstrated in the tables below, Table a) with all dorsalgia variants and Table b) with the pleiotropic variants removed.

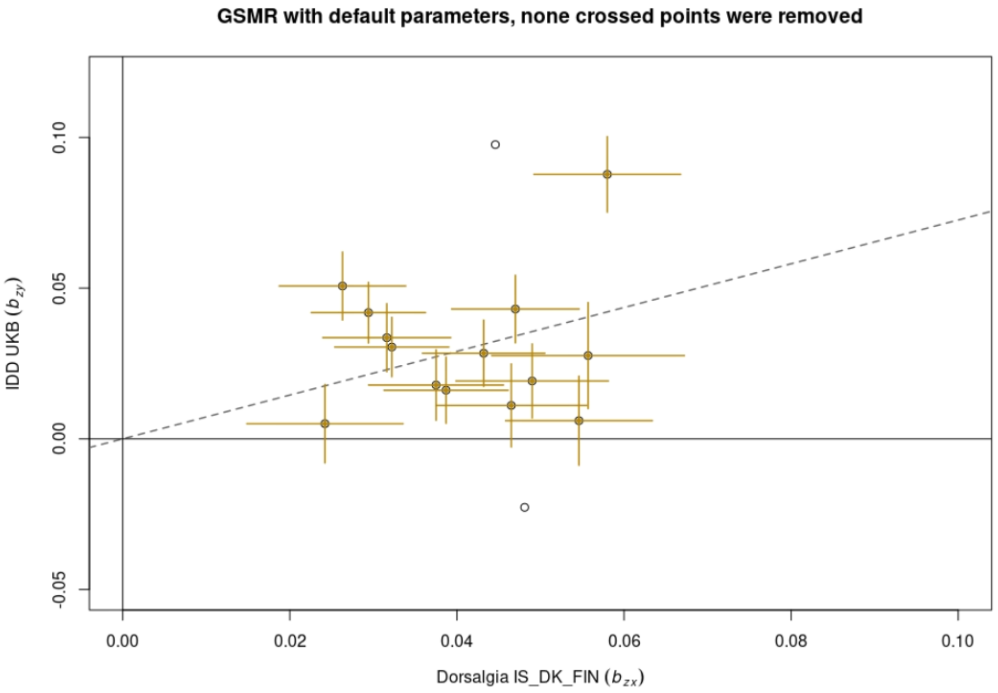

Exposure Variable

**a) All Dorsalgia variants:**	Estimate (95% CI)	P
Intercept only	0.03 (0.02, 0.05)	0.0005
Slope only[a]	0.80 (0.44, 1.17)	0.0006
Intercept given slope	0.02 (-0.05, 0.09)	0.52
Slope given intercept[b]	0.31 (-1.28, 1.86)	0.72

[a]IVW (inverse variance weighted), [b]MR Egger

Exposure Variable

**b) Dorsalgia variants, excluding two pleiotropic variants:**	Estimate (95% CI)	P
Intercept only	0.03 (0.018, 0.041)	0.00022
Slope only[a]	0.73 (0.47, 0.98)	0.0001
Intercept given slope	0.0047 (-0.04, 0.05)	0.85
Slope given intercept[b]	0.62 (-0.51, 1.75)	0.31

[a]IVW (inverse variance weighed), [b]MR Egger

Figure 4. *SLC13A1* and *SLC26A2* variants' association with IDD and sulfate levels, in Iceland and Amish (From Tise et al.[4]) (Blank cells in table refer to variants not found in the respective data)

						Intervertebral disc disorders				Sulfate effect Iceland (N = 315)		Sulfate effect Amish (N = 977)	
rsname	Gene	Pos_hg38	Coding	Hom/Het	MAF Eur/Ice	OR met	P meta	OR ice	P ice	Effect (SD)	P	Effect (ß)	P
rs28364172	*SLC13A1*	chr7:123199913	p.Arg12Ter	4/1033	0.36 / 0.32	1.414	2.54E-08	1.706	1.92E-04	-1.461	4.53E-03	-0.88	7.50E-06
rs138275989	*SLC13A1*	chr7:123181057	p.Trp48Ter	0/20	0.097 / 0.010	1.388	1.21E-04	0.017	0.215			-0.82	2.70E-08
rs2140516	*SLC13A1*	chr7:123169180	p.Asn174Ser		34.08 / 33.66	0.991	0.176	0.995	0.802	0.004	0.958	0.12	1.60E-04
rs139376972	*SLC13A1*	chr7:123147262	p.Arg237His		0.15 / 0.45	1.004	0.939	0.972	0.844	0.124	0.789	0.015	0.150
rs140244991	*SLC13A1*	chr7:123134528	p.Arg148Cys	0/100	0.032 / 0.030	1.998	6.24E-06	1.106	0.840				
rs148386572	***SLC26A1***	chr4:989896	p.Leu348Pro	4/684	0.25 / 0.20	1.120	0.012	1.258	0.241	-1.010	0.258	-0.046	4.40E-12

a)

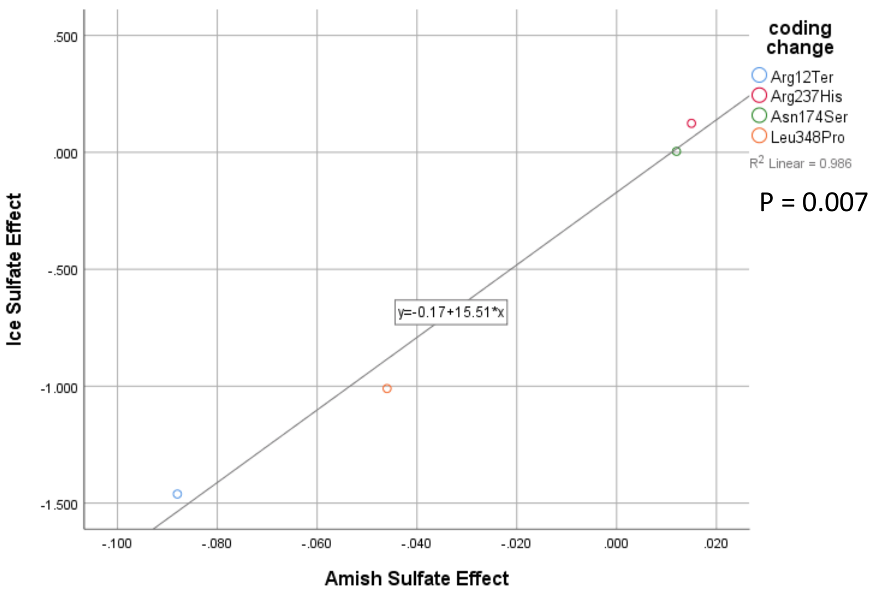

b)

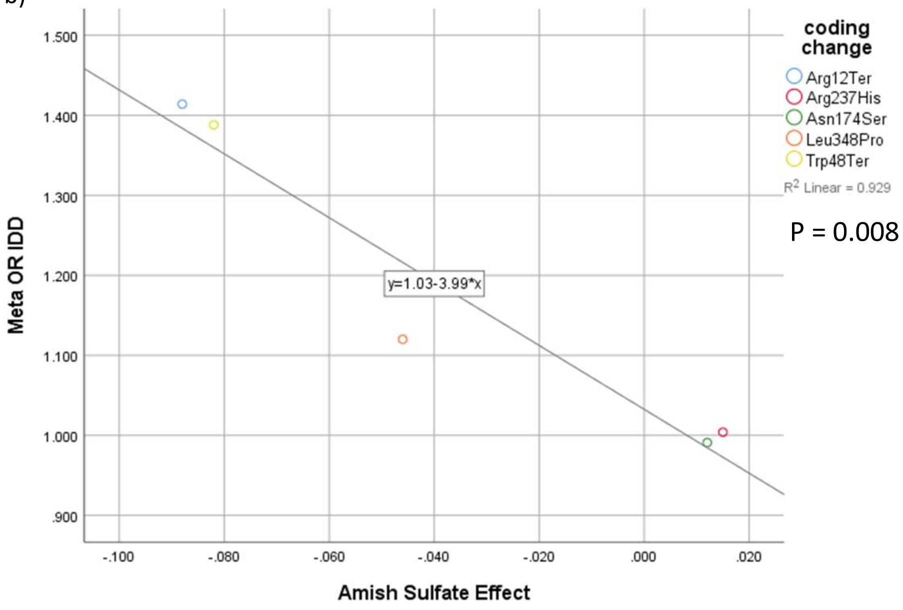

a) For each variant (color coded) in the table, its effects on sulfate levels in Icelandic data (y-axis) are plotted against its effects on sulfate levels as reported in Tise et al.
b) For each variant (color coded) in the table, its effect on IDD in the GWAS meta-analysis (Y-exis) is plotted agaits its effects on sulfate levels as reported in Tise et al.

Figure 5. Diagnoses and prescribed drugs among *SLC13A1* p.Arg12Ter homozygotes

Among whole-genome sequenced Icelanders (Methods) we identify four homozygous carriers (here identified by letters A to D) of the p.Arg12Ter loss of function mutation. Table S6 includes diagnostic data from the Icelandic Healthcare system under ICD-10 chapter M (Musculoskelatal disorders)

Carrier	Sex	Age (5yr bin)	ICD10 diagnoses from chapter M	Prescription drugs: ATC codes for Analgesics
A	Fem	75	M17 (Gonarthrosis), M75 (Shoulder lesions), M79 (Myalgia), M72 (Fibroblastic disorder), G61 (Inflammatory neuropathy), M25 (Pain in joint), **M54 (Dorsalgia)**	N02AJ06 codeine and paracetamol
B	Male	80	M75 (Shoulder lesions), M25 (Joint disorders, not elsewhere classified), **M51 (Intervertebral disc disorders)**	N02AJ06 codeine with paracetamol
C	Fem	75	M17 (Gonarthrosis), G56 (Carpal tunnel syndrome), M23 (Cystic meniscus), R52 (Pain, unspecified), M60 (Myositis), M54 (Dorsalgia), M79 (Myalgia), M25 (Joint disorders, not elsewhere classified), M77 (Other enthesopathies), M70 (Bursitis), **M51 (Intervertebral disc disorders),** M40 (Kyphosis/Lordosis)	N03AX12 gabapentin
D	Fem	60	M17 (Gonarthrosis), M23 (Cystic meniscus), M79 (Myalgia), M77 (Other enthesopathies), M25 (Joint disorders, not elsewhere classified), **M54 (Dorsalgia), M51 (Intervertebral disc disorders)**	N02AJ06 codeine with paracetamol N03AX12 gabapentin

Figure 6. Mendelian randomization: Inflammatory Bowel Disease (IBD) and dorsalgia

Inflammatory Bowel Disease (IBD) effects on Dorsalgia. Using as instruments 220 IBD variants from Liu et al. (2015)[5]. Effects from 15 IBD GWASs and Immunochip meta-analysis on individuals of European descent (N_{total} cases = 38,155, N_{total} controls = 48,485).

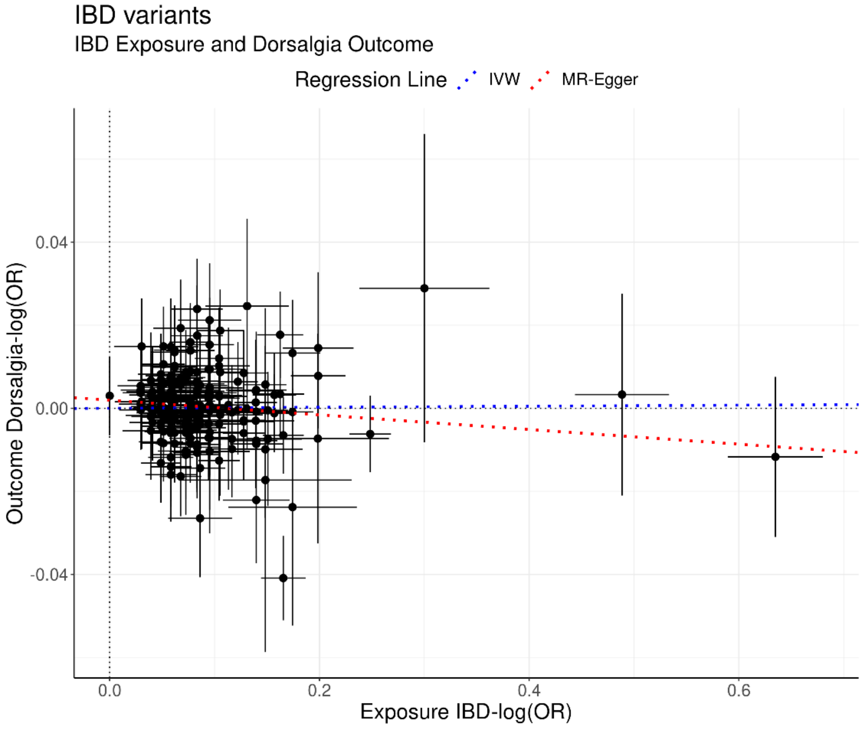

Variable	Dorsalgia Estimate (95% CI)	P
IBD variants:		
Intercept only	1.00 (1.00, 1.00)	0.30
Slope only (IVW)	1.00 (0.99, 1.01)	0.83
Intercept given slope	0.98 (0.96, 1.01)	0.14
Slope given intercept (MR-Egger)	1.00 (1.00, 1.00)	0.08

IVW (inverse variance weighted)

The figure S6. above shows effects of variants associating with IBD at genome-wide significance, on IBD and dorsalgia. Effects are expressed as logarithms of odds ratios (log(OR)) and black crosses indicate 95% confidence intervals (CI) around effects. The dashed blue line shows the linear regression fit through the origin, weighting variants according to the square of the standard error of their effect estimates (also known as inverse variance weighted, IVW)). The IVW-MR method is a multiplicative random effects model, where the test statistic is from a t-distribution, the test is two-sided. No multiple comparison adjustments were made. The dashed red lines show the weighted linear regression fit not constrained to go through the origin (also known as MR Egger).

Figure 7. Mendelian randomization: Osteoarthritis – IDD & Dorsalgia

A) Osteoarthritis (OA) effects on IDD. Using as instruments 18 OA variants from Boer et al (in press)[6]. Effects from OA GWAS meta-analysis on 16 groups of European descent (N_{total} cases = 78,610, N_{total} controls = 100,164).

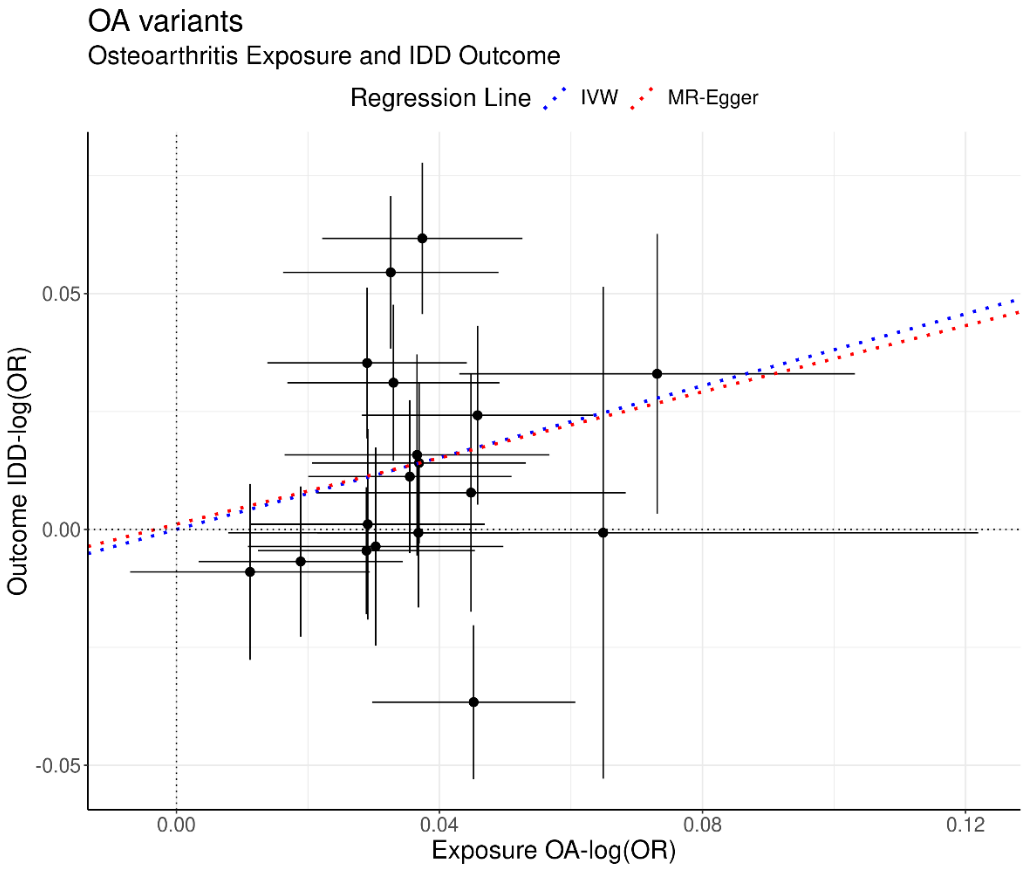

Variable	IDD Estimate (95% CI)	*P*
<u>*OA variants*</u>*:*		
Intercept only	1.01 (1.00, 1.03)	0.05
Slope only (IVW)	1.46 (1.05, 2.05)	0.04
Intercept given slope	1.00 (0.96, 1.04)	0.96
Slope given intercept (MR-Egger)	1.42 (0.44, 4.62)	0.57

IVW (inverse variance weighted)

The figure above shows effects of variants associating with OA at genome-wide significance, on OA and IDD. Effects are expressed as logarithms of odds ratios (log(OR)) and black crosses indicate 95% confidence intervals (CI) around effects. The dashed blue line shows the linear regression fit through the origin, weighting variants according to the square of the standard error of their effect estimates (also known as inverse variance weighted, IVW)). The IVW-MR method is a multiplicative random effects model, where the test statistic is from a t-distribution, the test is two-sided. No multiple comparison adjustments were made. The dashed red lines show the weighted linear regression fit not constrained to go through the origin (also known as MR Egger).

b) Osteoarthritis (OA) effects on Dorsalgia. Using as instruments 18 OA variants from Boer et al (in press)[6]. Effects from OA GWAS meta-analysis on 16 groups of European descent (N_{total} cases = 78,610, N_{total} controls = 100,164).

B) Osteoarthritis (OA) effects on Dorsalgia. Using as instruments 18 OA variants from Boer et al (in press)[6]. Effects from OA GWAS meta-analysis on 16 groups of European descent (N_{total} cases = 78,610, N_{total} controls = 100,164).

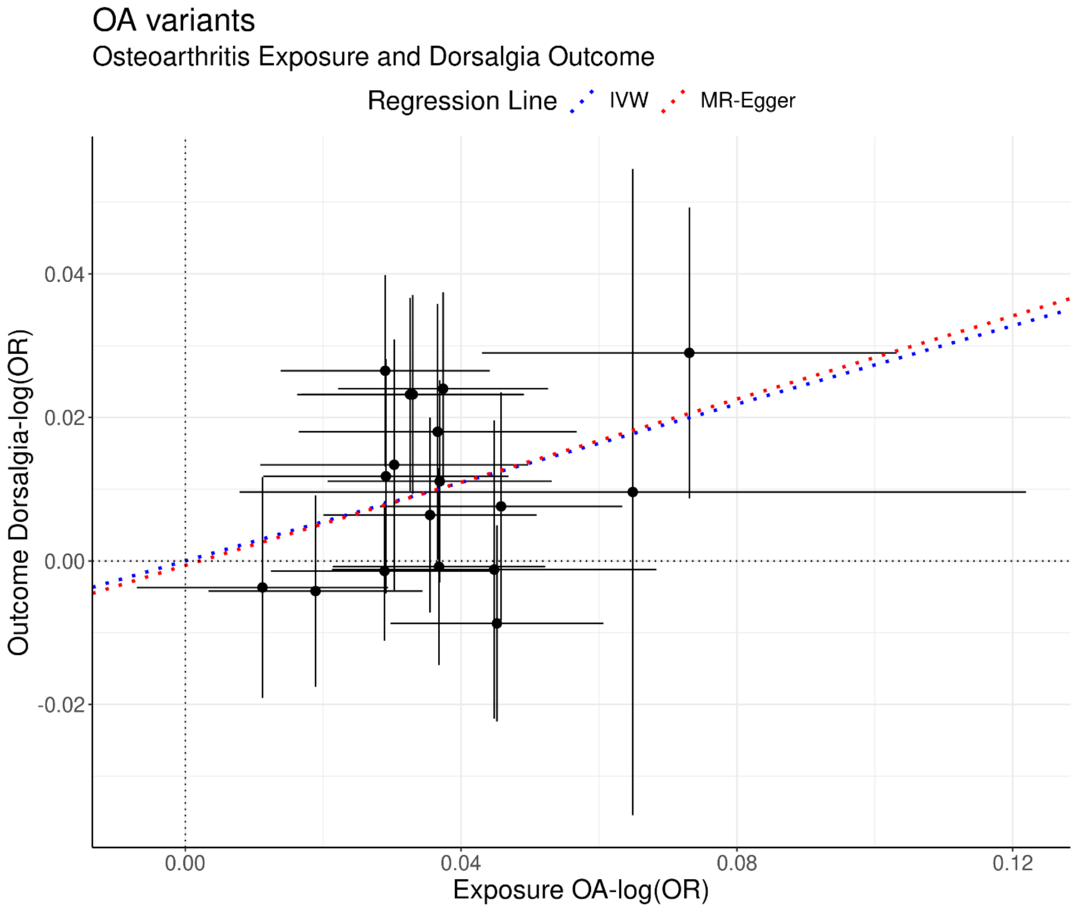

Variable	Dorsalgia Estimate (95% CI)	P
OA variants:		
Intercept only	1.31 (1.13, 1.53)	0.003
Slope only (IVW)	1.01 (1.00, 1.02)	0.005
Intercept given slope	1.00 (0.98, 1.02)	0.95
Slope given intercept (MR-Egger)	1.34 (0.80, 2.23)	0.29

IVW (inverse variance weighted)

The figure above shows effects of variants associating with OA at genome-wide significance, on OA and Dorsalgia. Effects are expressed as logarithms of odds ratios (log(OR)) and black crosses indicate 95% confidence intervals (CI) around effects. The dashed blue line shows the linear regression fit through the origin, weighting variants according to the square of the standard error of their effect estimates (also known as inverse variance weighted, IVW)). The IVW-MR method is a multiplicative random effects model, where the test statistic is from a t-distribution, the test is two-sided. No multiple comparison adjustments were made. The dashed red lines show the weighted linear regression fit not constrained to go through the origin (also known as MR Egger).

Supplementary Notes

Note 1. Role of *SLC13A1* and *CHST3* in sulfate availability and sulfation in proteoglycan synthesis

Sulfate is an obligate nutrient for fetal growth and development[7-9]. Here, we focus on its role in healthy intervertebral disc development[10-12] and refer to informative figures in A)Paganini et al. (2019)[13], B) Tise et al. (2016)[4], and, C) at: http://www.medicinalchemistry.org.

A) We refer to Paganine et al.[13] for a schematic view of proteoglycan (PG) synthesis which is a complex mechanism that can be divided in four main steps. (1) Core protein synthesis occurs in the rough endoplasmic reticulum (RER). Once PG core protein has been synthesized, it moves from the RER to the Golgi apparatus where the first sugar of glycosaminoalycan (GAG) chain is added on Ser residues. (2) GAG synthesis continues by glycosyltransferases that transfer sugar moieties from UDP-sugars to GAG chains. UDP-sugars are synthesized in the cytoplasm and are translocated in the Golgi apparatus by an antiporter with UMP. Then UDP, the by-product of glycosyltransferase reactions, is hydrolyzed to UMP and phosphate by calcium activated nucleotidase 1 (CANT1). (3) The third step is GAG sulfation. Sulfate enters in cells through the SLC26A2 transporter and it is activated to 3'-phosphoadenosine 5'-phosphosulfate (PAPS) by PAPS synthase (PAPSS) in the cytosol. Through a PAPS transporter (PAPST), PAPS moves to Golgi apparatus where it is used as sulfate donor by sulfotransferases to sulfate GAGs. This reaction also produces phosphoadenosine phosphate (PAP), that is hydrolyzed into AMP and phosphate by a Golgi resident phosphoadenosine phosphate phosphatase (gPAPP). (4) Once synthesized, PGs are secreted in extracellular space.

B) We refer to Tise et al.[4] for a figure depicting how sulfate availability in blood is regulated. Step 3 in PG synthesis as depicted in A) Paganine et al.[13] , requires adequate sulfate availability in blood which is regulated by SLC13A1, an apical sodium-sulfate cotransporter (NaS1) responsible for sulfate (re)absorption in the kidneys and intestine, and SLC26A1, a basolateral membrane, sulfate-anion transporter 1 (Sat1) that is expressed at high levels in developing and mature cartilage, as well as in lung, placenta, colon, kidney, pancreas and testis.

C) The figure at http://www.medicinalchemistry.org shows sulfated proteoglycans, the end product of the processes described above and the building blocks of healthy intervertebral discs.

Note 2. Back pain variants with functional evidence implicating specific genes

a) IDD genes

SLC13A1 (Solute carrier family 13 member 1) encodes a protein that is an apical membrane Na+-sulfate cotransporter involved in homeostasis function of kidneys. It is selectively expressed in renal tubule of the kidney and intestine) GTEx: https://gtexportal.org/home/) where it regulates respectively re-uptake of sulfate from urine and uptake of sulfate from diet. Inorganic sulfate is abundant in plasma and present

in nearly all cell types. Sulfate plays an essential role during growth, development, bone/cartilage formation, and cellular metabolism. Disturbances in sulfate supply and metabolism, regulated by SLC13A1 and SLC26A1, that transports sulfate across the plasma membrane, can cause a variety of fatal syndromes and several congenital chondrodysplasias (OMIM#606193 & #606718).

SERPINA1 (*Serpin family A member 1*) encodes a serine protease inhibitor belonging to the serpin superfamily whose targets include elastase, plasmin, thrombin, trypsin, chymotrypsin, and plasminogen activator. Variants in this gene associate with a range of metabolic and inflammatory traits including C-reactive protein levels, bone mineral density, lung function, height, blood pressure and more (OMIM#107400, Supplementary Data 16).

MYPOP (*Myb-related transcription factor, partner of profilin*); a gene with ubiquitous expression in brain, spleen and many other tissues. Is previously GWAS-associated with mean corpuscular hemoglobin and recently identified as a restriction factor of Human papillomavirus infection[14] (Supplementary Table 14).

CNNM2 (*Cyclin M2*), encoding a transmembrane protein involved in magnesium transport, is highly expressed in brain, kidney and lung and harbors variants that have been linked to hypomagnesemia, autosomal dominant and recessive forms with seizures and mental retardation.

FGFR3 (*Fibroblast growth factor receptor 3*) is a growth factor involved in bone development that is linked to various skeletal dysplasias, including achondroplasia and hypochondroplasia.

TGFA (*Transforming growth factor alpha*) is a growth factor involved in bone development that has been linked to familial cleft lip (OMIM). The IDD variant in *TGFA* also associates with osteoarthritis of the hip and hand grip strength (Supplementary Data 16).

GFPT1 (*Glutamine fructose-6-phosphate amidotransferase 1*) is the first, rate-limiting enzyme of the hexosamine biosynthetic pathway and has been linked to recessive congenital myasthenic syndrome (OMIM #138292) and synthesis of proteoglycans[15]. The allele of the splice-region variant in *GFTP1* that increases IDD risk, is also the top cis-eqtl association in blood at this locus ($z = 62.4$, $P = 3.3 \times 10^{-310}$).

ARNTL encodes a basic HLH protein that forms a heterodimer with CLOCK, that binds to E-box enhancer elements upstream of Period (*PER1, PER2, PER3*) and Cryptochrome (*CRY1, CRY2*) genes and activates

their transcription. PER/CRY heterodimers interact with *CLOCK/ARNTL* complexes downregulating expression of PER and CRY. Defects in *ARNTL* have been linked to infertility, gluconeogenesis and lipogenesis, and altered sleep patterns (OMIM #602550).

SPON2 (*Spondin 2*) may act as an opsonin and recognition molecule for a range of pathogens through detection of carbohydrate structures and activation of macrophages. A variant in the gene has been associated with mean corpuscular hemoglobin (Supplementary Data 16).

CD79B (*CD79B antigen*) encodes the Ig-beta protein of the B-cell antigen component, that forms a disulfide-linked heterodimer with Ig-alpha (CD79A), bringing the IgM heavy chain to the cell surface to form the functional B-cell antigen receptor complex. Missense and LOF variants in *CD79B* cause autosomal recessive Agammaglobulinemia 6 and associations of common variants have been reported with body height and BMI measures (OMIM #147245, Supplementary Data 16).

GDF5 (*Growth/differentiation factor 5*) also known as cartilage-derived morphogenic protein 1, belongs to the TGF-beta superfamily of growth factors/signaling molecules and is closely related to the bone morphogenetic proteins. Autosomal dominant and recessive variants in this gene associate with severe dysplasias (OMIM #601146) and GWAS catalogue lists several associations for the IDD variant and correlated variants, including osteoarthritis, height and bone size measures (Supplementary Data 16).

b) Dorsalgia genes

MKRN1 (*Makorin ring finger protein-1 gene*) is highly and uniformly expressed in all tissues, including different regions of the brain (GTEx: https://gtexportal.org/home/), with high levels observed in murine embryonic nervous system and adult testis[16]. Previously shown to regulate protein homeostasis and contribute to mRNA quality control, *MKRN1* has recently been found to affect neuronal membrane excitability by regulating protein homeostasis of the Eag1 potassium channel[17].

UQCC1 (*Ubiquinol-cytochrome C reductase complex chaperone 1*) encodes a transmembrane protein structurally similar to the mouse basic fibroblast growth factor repressed ZIC-binding protein, involved in

FGF-controlled growth processes in the mouse, and in humans, associations have been reported with many traits, including body height, BMI measures, and osteoarthritis (**GWAS catalogue, URLs**).

EIF4E3 (*Eukaryotic translation initiation factor 4E family member 3*) is widely expressed in tissues and involved in mRNA translation

SNRPC (*Small nuclear ribonucleprotein polypetide C*) is widely expressed in tissues and involved in mRNA translation.

SIGLECL1 (*Sialic acid-binding immunoglobulin-like lectin 12*) encodes a cell surface protein of the Ig superfamily and mainly expressed in the immune system[18].

APOE. The missense variant (p.Cys130Arg, rs429358-C), representing the *APOE4* allele that increases risk of Alzheimer's disease[19], also associates with dorsalgia (OR = 0.96, P = 1.97 x 10^{-11}), but not with IDD (OR = 0.99, P = 0.20). The reduced risk of dorsalgia associated with this variant is consistent across all four datasets with P_{het} = 0.148 (Supplementary Data 4).

Note 3. Gene set enrichment and pathway analyses

Using MAGMA[20], as implemented by FUMA v.1.3.2 (https://fuma.ctglab.nl/)[21] we performed a gene-based and gene set enrichment analysis to identify tissues and etiological pathways relevant to the back pain diagnoses IDD and dorsalgia. See interpretation of figure outputs at: https://fuma.ctglab.nl/tutorial#g2fOutputs, repeated here:

Differentially Expressed Gene (DEG) Sets

DEG sets were pre-calculated by performing two-sided t-test for any one of labels against all others. For this, expression values were normalized (zero-mean) following to a log 2 transformation of expression value (EPKM or TPM). Genes which with P-value ≤ 0.05 after Bonferroni correction and absolute log fold change ≥ 0.58 were defined as differentially expressed genes in a given label compared to others. On top of DEG, up-regulated DEG and down-regulated DEG were also pre-calculated by taking sign of t-statistics into account. Input genes were tested against each of the DEG sets using the hypergeometric test. The background genes are genes that have average expression value > 1 in at least one of the labels and exist in the user selected background genes. Significant enrichment at Bonferroni corrected P-value ≤ 0.05 are colored in red. Note that for DEG sets, Bonferroni correction is performed for each of up-regulated, down-regulated and both-sided DEG sets separately.

Gene Expression Heatmap

The heatmap displays two expression values.

1) Average expression per label

This is an averaged expression value per label (e.g. tissue types or developmental stage) per gene following to winsorization at 50 and log 2 transformation with pseudocount 1. The expression value depends on the data set, RPKM (Read Per Kilobase per Million) for GTEx v6 and BrainSapn, TPM (Transcripts Per Million) for GTEx v7. This allows for comparison across labels and genes. Hence, cells filled in red represent higher expression compared to cells filled in blue across genes and labels.

2) Average of normalized expression per label

This is the average of normalized expression (zero mean across samples) following to winsorization at 50 and log 2 transformation of the expression value with pseudocount 1. This allows comparison of gene expression across labels (horizontal comparison) within a gene. Thus, expression values of different genes within a label (vertical comparison) are not comparable. Hence, cells filled in red represents higher expression of the genes in a corresponding label compared to other labels, but it DOES NOT represent higher expression compared to other genes.

3.1. Intervertebral disc disorder (IDD) gene sets

3.1.1. IDD - Differentially Expressed Gene (DEG) Sets - 54 GTEx tissue types

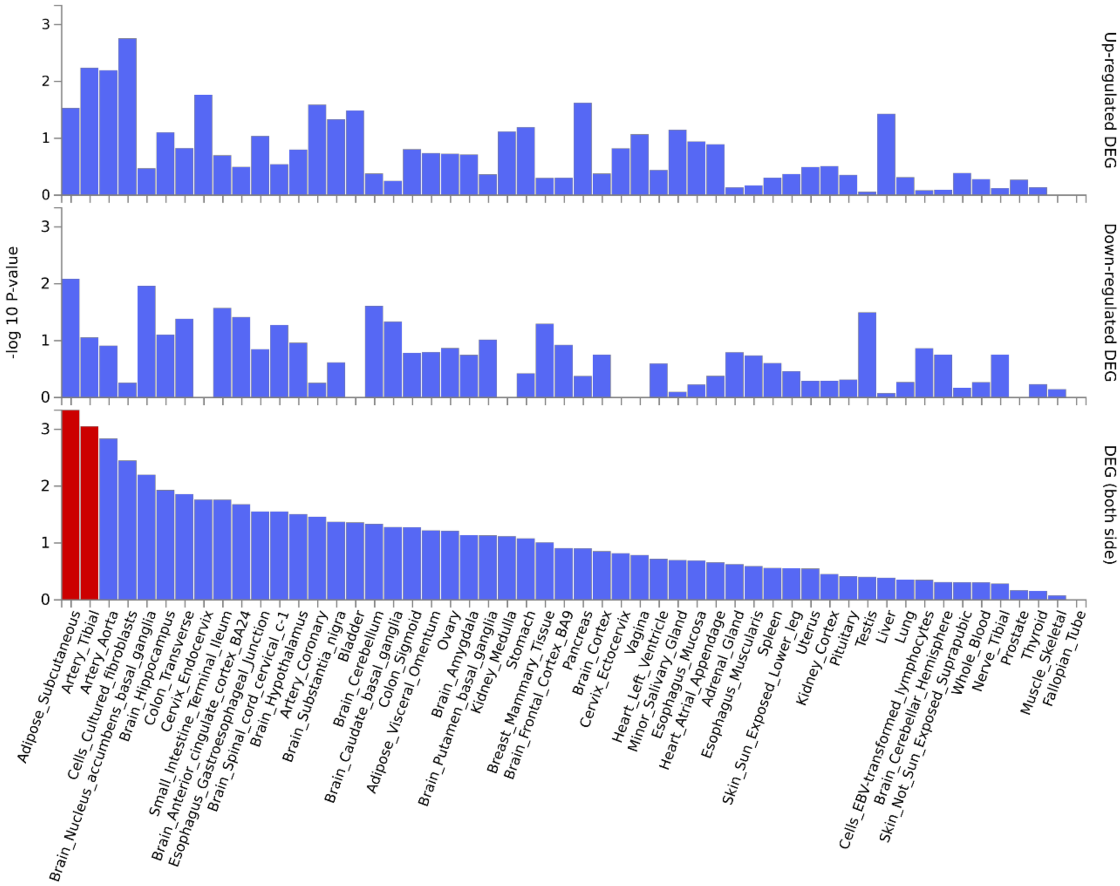

3.1.2. IDD – Gene Expression Heatmap

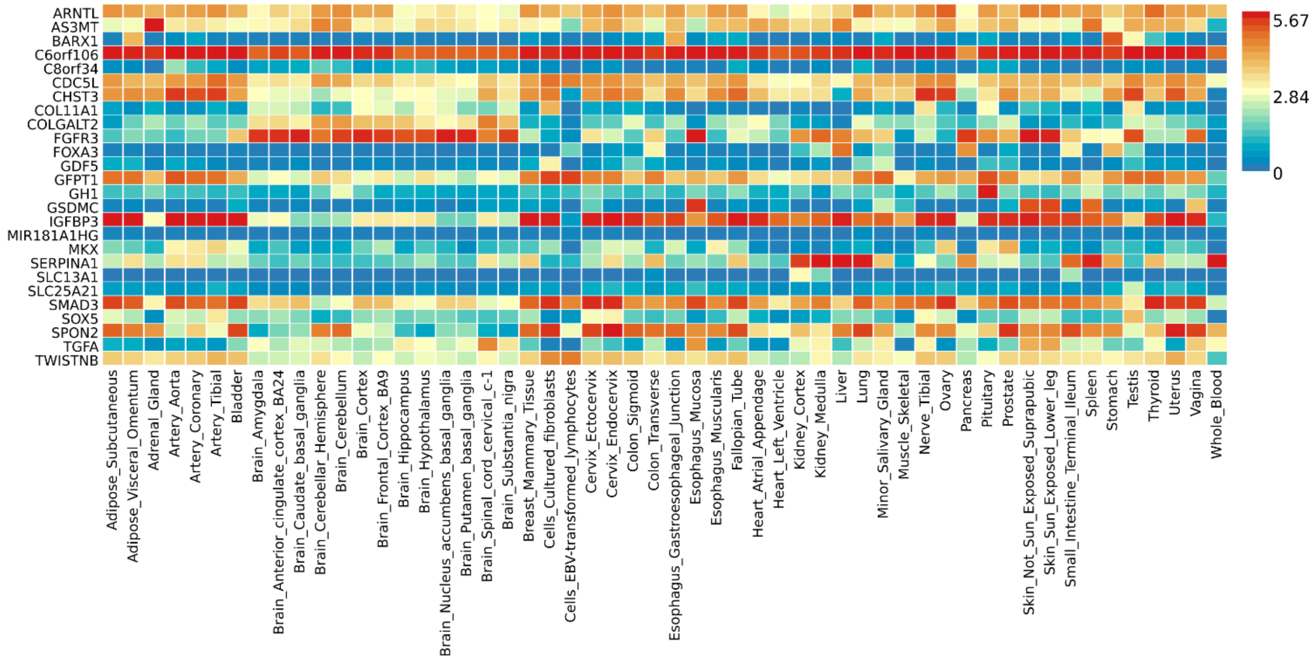

3.1.3 IDD - Brainspan Differentially Expressed Gene (DEG) Sets

BrainSpan expression for 11 general developmental stages of brain samples.

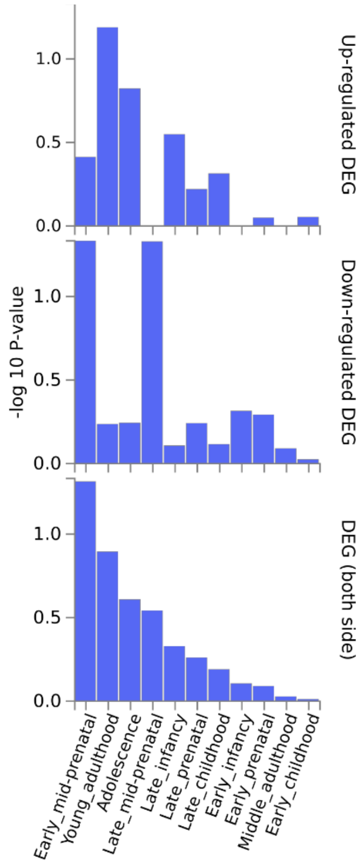

3.1.4. IDD - Brainspan Gene Expression Heatmap

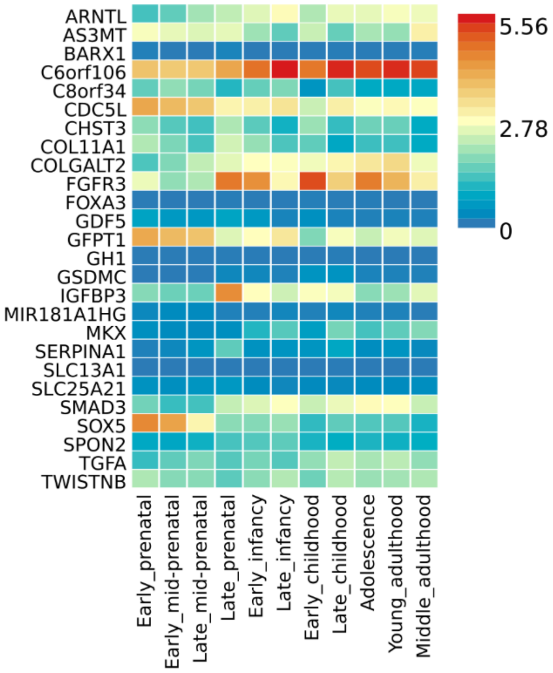

3.1.5. IDD gene sets – GWAS catalogue reported genes / WikiPathways

From https://fuma.ctglab.nl/tutorial#g2fOutputs: Hypergeometric tests are performed to test if genes of interest are overrepresented in any of the pre-defined gene sets. Multiple test correction is performed per category, (i.e. canonical pathways, GO biological processes and so on, separately). Gene sets were obtained from MsigDB, WikiPathways and reported genes from the GWAS-catalog.

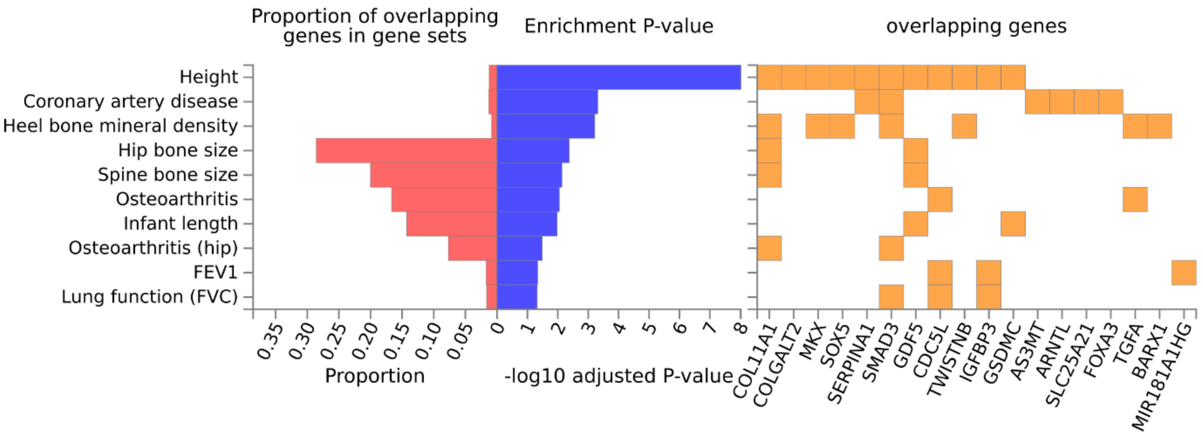

3.1.6. IDD Gene Sets – GO

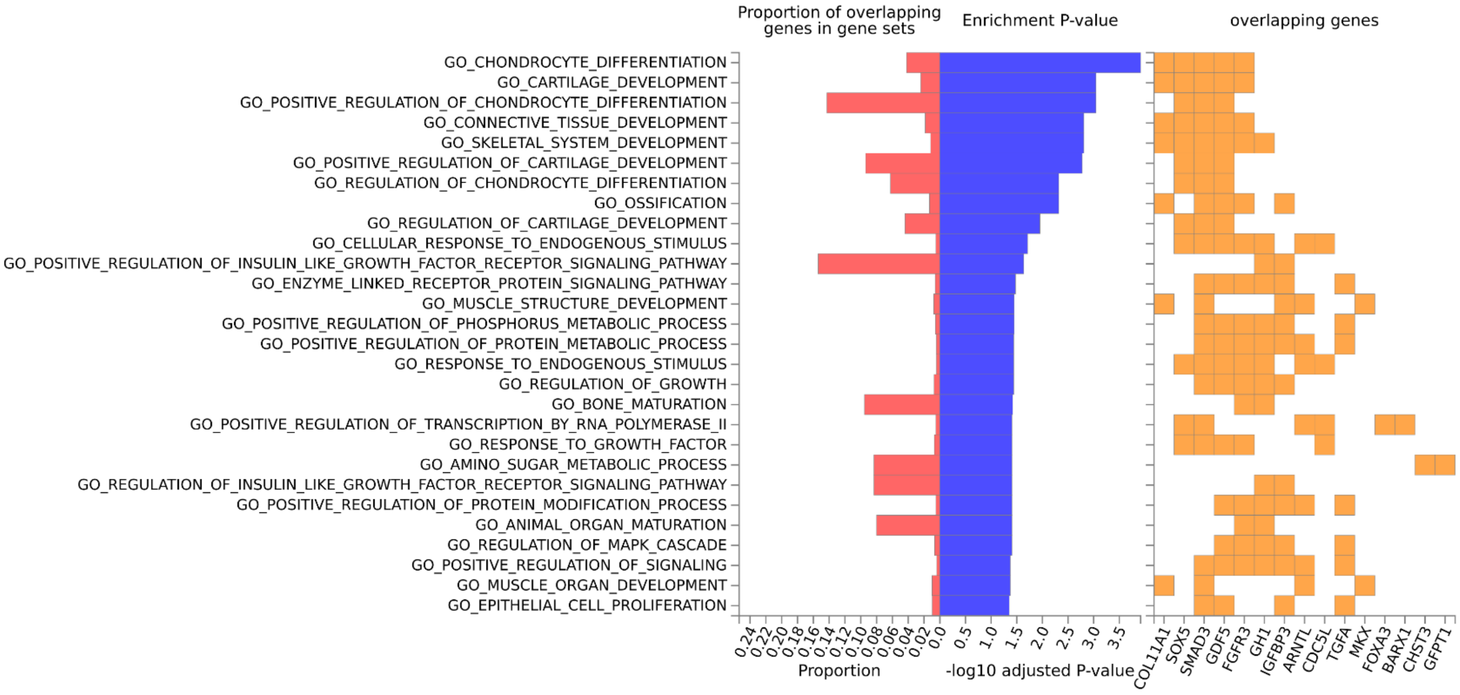

3.1.7. IDD Gene Sets – All canonical pathways

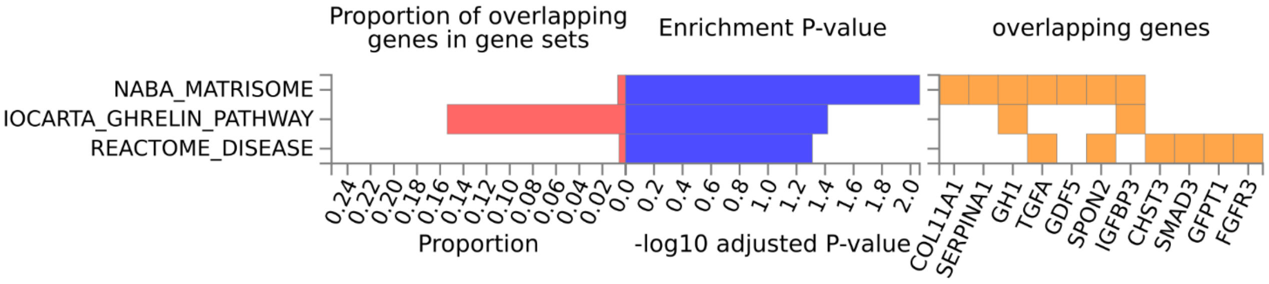

3.1.8 IDD Gene Sets – Immunological signature

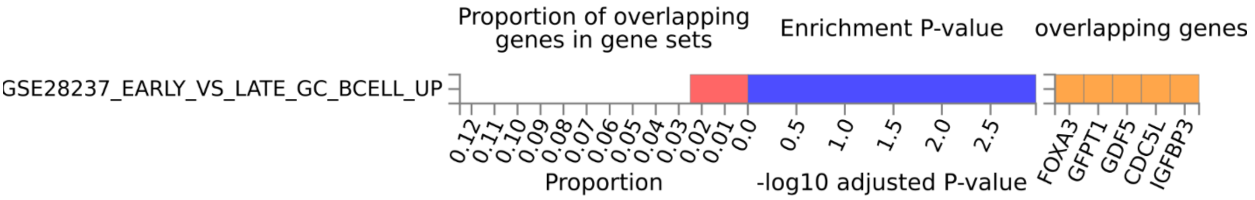

3.1.9. IDD Gene Sets – Curated gene sets

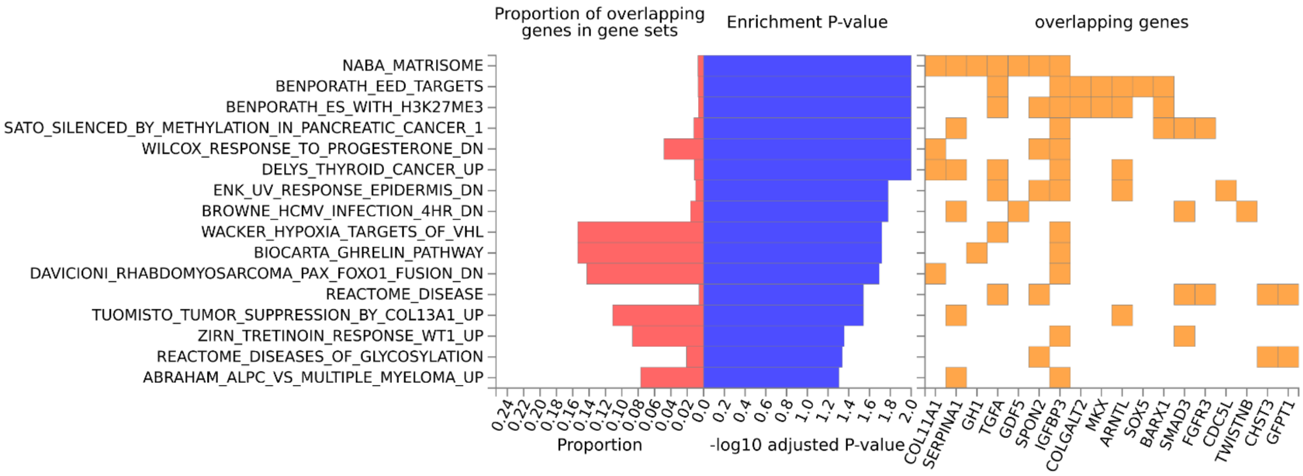

3.1.10. IDD Gene Sets – Chemical and genetic Perturbation

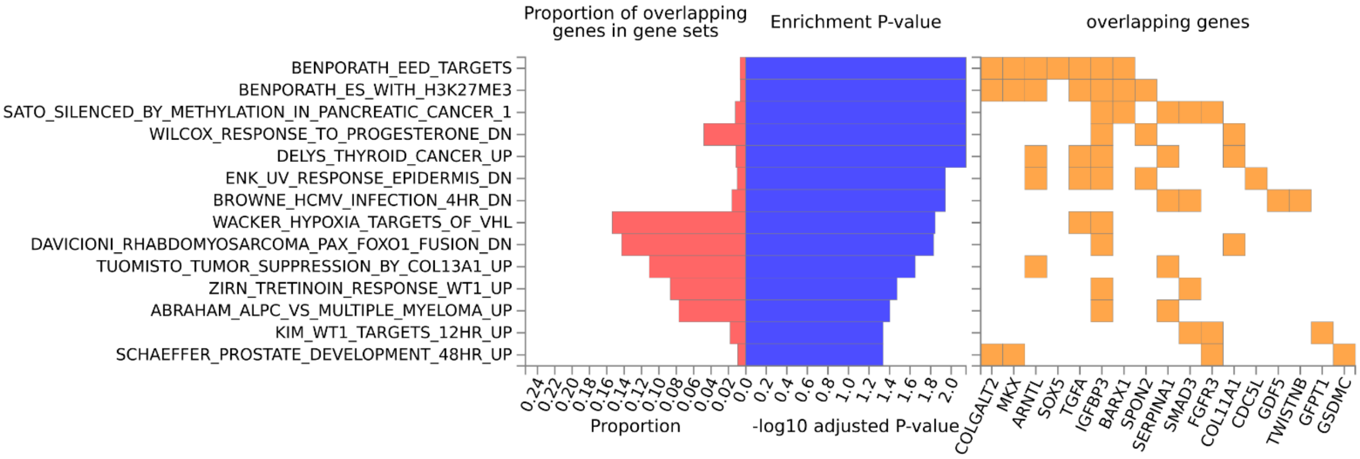

3.1.11. IDD Gene Sets – Hallmark

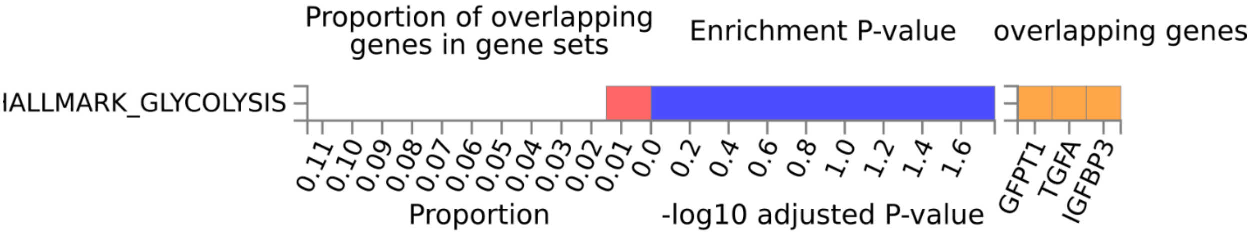

3.1.12. IDD Gene Sets – Cancer modules

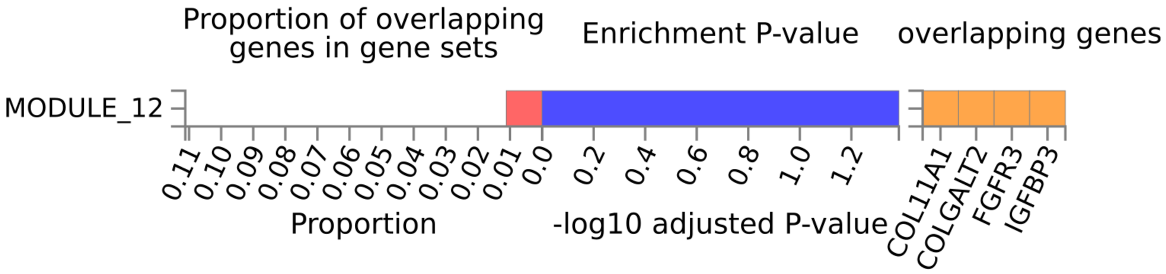

3.2. Dorsalgia gene sets

See interpretation of figure outputs on page 39 above.

3.2.1. Dorsalgia - Differentially Expressed Gene (DEG) Sets

Tissue specificity analysis across 54 tissue types from GTEx.

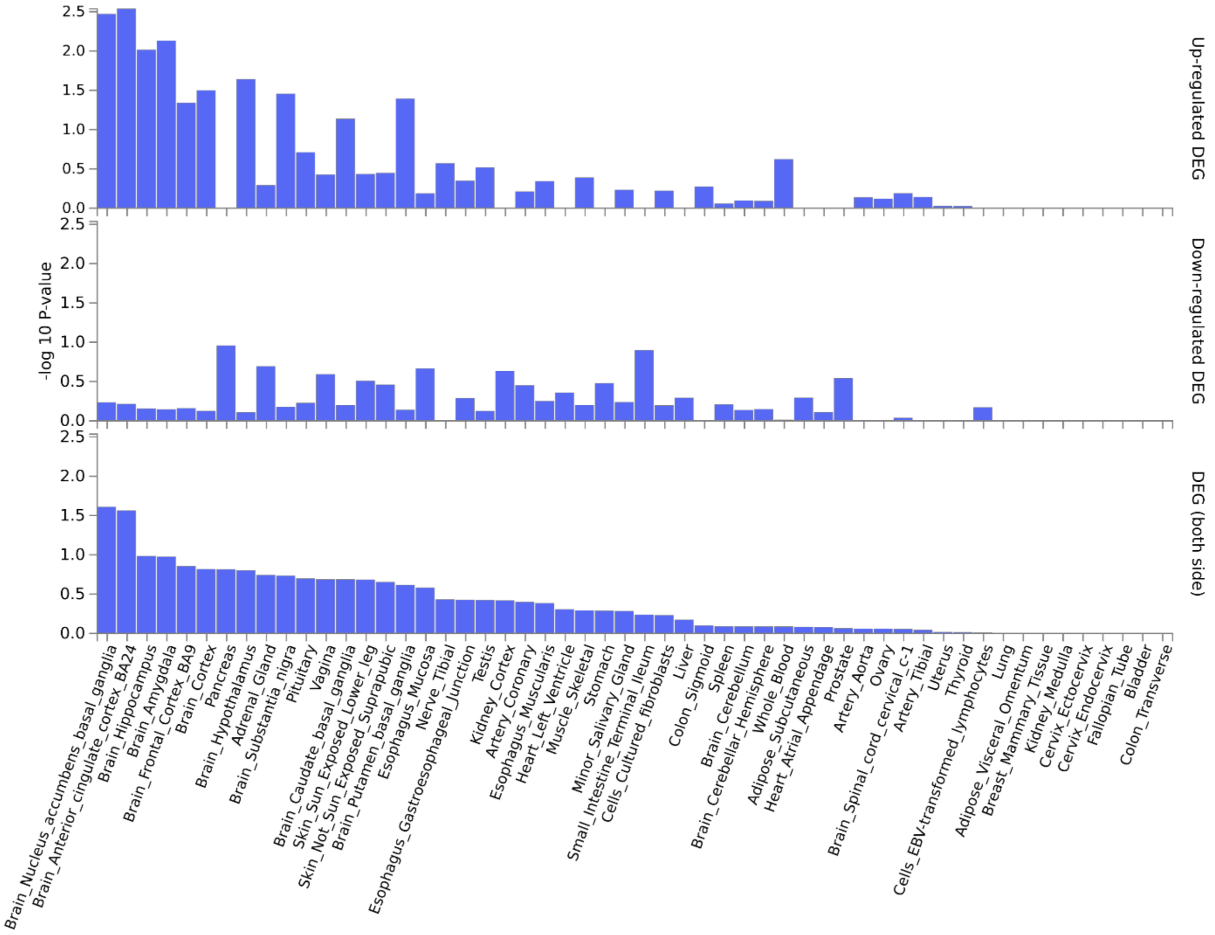

3.2.2. Dorsalgia – GTEx Expression Heatmap (54 tissue types)

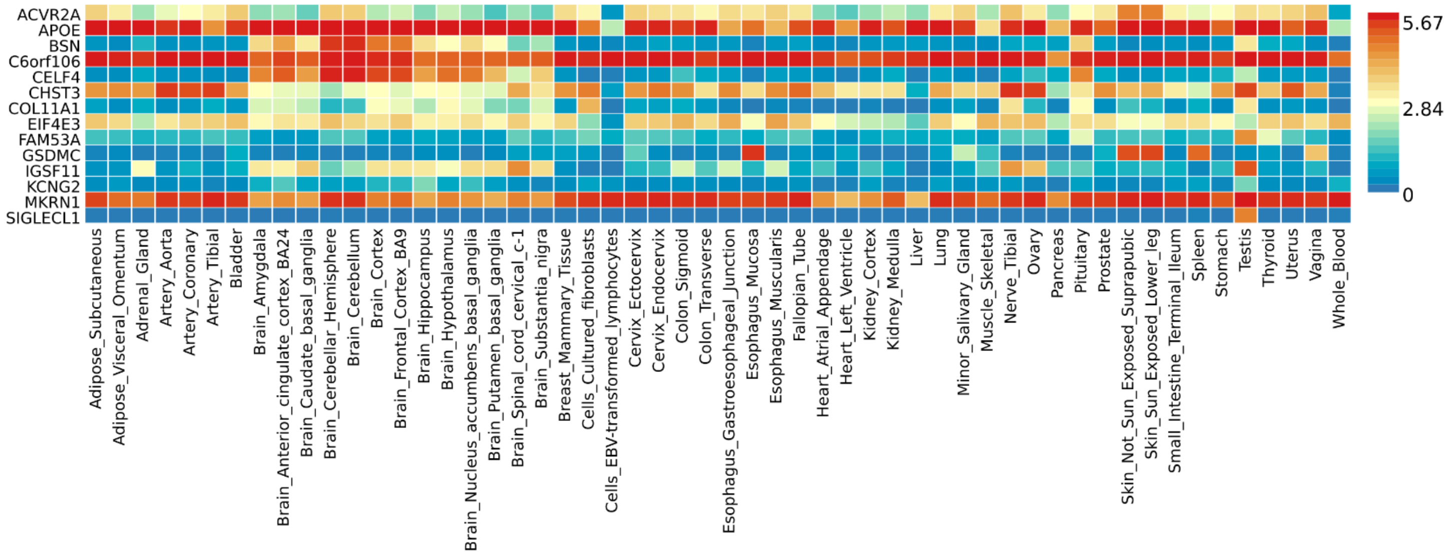

3.2.3 Dorsalgia - Brainspan Differentially Expressed Gene (DEG) Sets

11 general developmental stages of brain samples

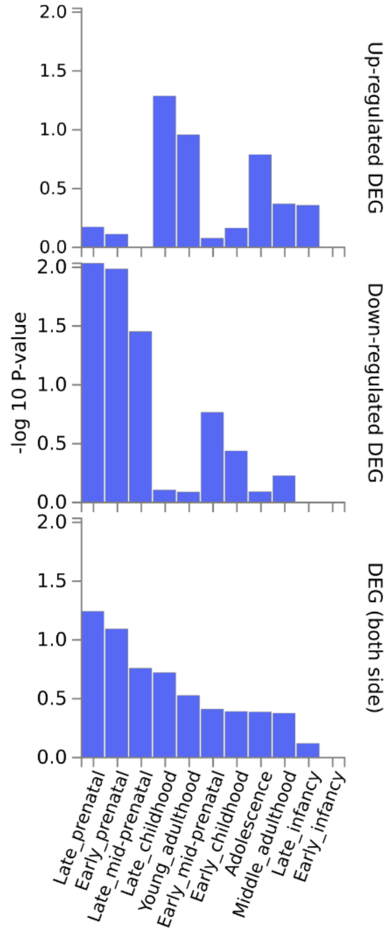

3.2.4. Dorsalgia - Brainspan Gene Expression Heatmap

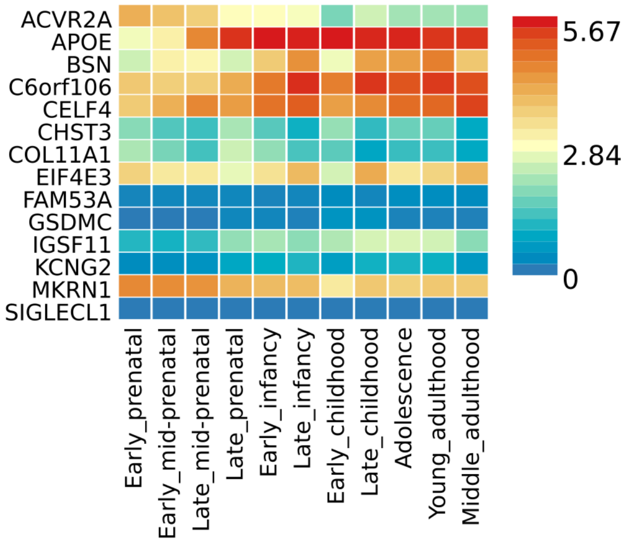

3.2.5. Dorsalgia gene sets – GWAS catalogue

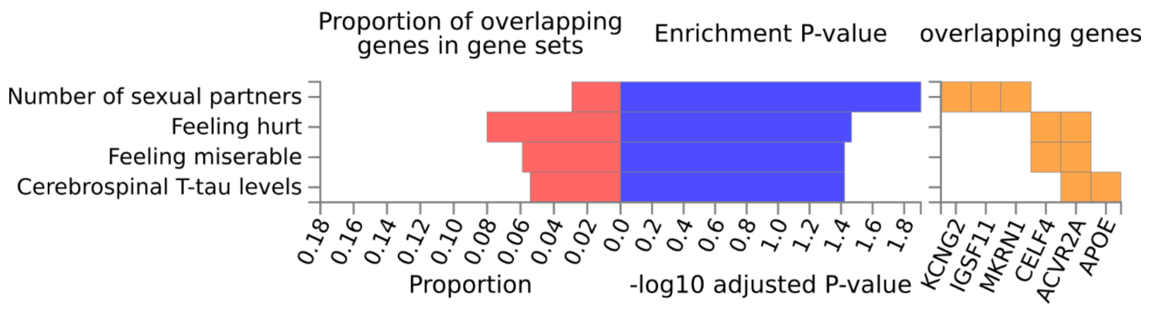

3.2.6. Dorsalgia gene sets – MicroRNA targets

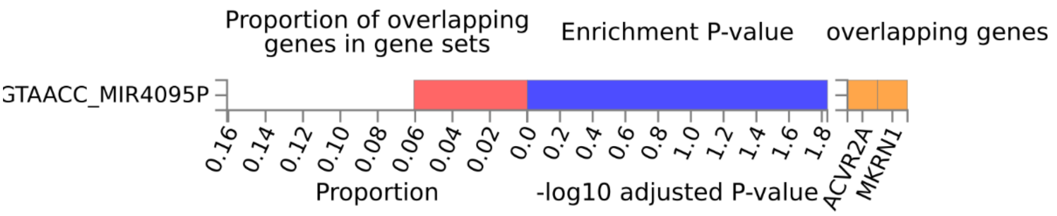

References

1. World Health Organization (WHO). ICD-10 : International statistical classification of diseases and related health problems : tenth revision. 2nd ed (World Health Organization, Geneva, 2004).
2. Leutgeb, R., Engeser, P., Berger, S., Szecsenyi, J. & Laux, G. Out of hours care in Germany - High utilization by adult patients with minor ailments? *BMC Fam Pract* **18**, 42 (2017).
3. Zhu, Z. *et al.* Causal associations between risk factors and common diseases inferred from GWAS summary data. *Nat Commun* **9**, 224 (2018).
4. Tise, C.G. *et al.* From Genotype to Phenotype: Nonsense Variants in SLC13A1 Are Associated with Decreased Serum Sulfate and Increased Serum Aminotransferases. *G3 (Bethesda)* **6**, 2909-18 (2016).
5. Liu, J.Z. *et al.* Association analyses identify 38 susceptibility loci for inflammatory bowel disease and highlight shared genetic risk across populations. *Nat Genet* **47**, 979-986 (2015).
6. Boer, C.G. *et al.* Deciphering osteoarthritis genetics across 826,690 individuals from 9 populations. *Cell* **184**, 4784-4818 e17 (2021).
7. Dawson, P.A. Sulfate in fetal development. *Semin Cell Dev Biol* **22**, 653-9 (2011).
8. Dawson, P.A., Beck, L. & Markovich, D. Hyposulfatemia, growth retardation, reduced fertility, and seizures in mice lacking a functional NaSi-1 gene. *Proc Natl Acad Sci U S A* **100**, 13704-9 (2003).
9. Williams, R.J. Sulfate Deficiency as a Risk Factor for Autism. *J Autism Dev Disord* **50**, 153-161 (2020).
10. Markovich, D. Slc13a1 and Slc26a1 KO models reveal physiological roles of anion transporters. *Physiology (Bethesda)* **27**, 7-14 (2012).
11. Neff, M.W. *et al.* Partial deletion of the sulfate transporter SLC13A1 is associated with an osteochondrodysplasia in the Miniature Poodle breed. *PLoS One* **7**, e51917 (2012).
12. Zhao, X. *et al.* In a shake of a lamb's tail: using genomics to unravel a cause of chondrodysplasia in Texel sheep. *Anim Genet* **43 Suppl 1**, 9-18 (2012).
13. Paganini, C., Costantini, R., Superti-Furga, A. & Rossi, A. Bone and connective tissue disorders caused by defects in glycosaminoglycan biosynthesis: a panoramic view. *FEBS J* **286**, 3008-3032 (2019).
14. Wustenhagen, E. *et al.* The Myb-related protein MYPOP is a novel intrinsic host restriction factor of oncogenic human papillomaviruses. *Oncogene* **37**, 6275-6284 (2018).
15. Senderek, J. *et al.* Hexosamine biosynthetic pathway mutations cause neuromuscular transmission defect. *Am J Hum Genet* **88**, 162-72 (2011).
16. Gray, T.A. *et al.* The ancient source of a distinct gene family encoding proteins featuring RING and C(3)H zinc-finger motifs with abundant expression in developing brain and nervous system. *Genomics* **66**, 76-86 (2000).
17. Fang, Y.C. *et al.* Identification of MKRN1 as a second E3 ligase for Eag1 potassium channels reveals regulation via differential degradation. *J Biol Chem* **296**, 100484 (2021).
18. Crocker, P.R. & Redelinghuys, P. Siglecs as positive and negative regulators of the immune system. *Biochem Soc Trans* **36**, 1467-71 (2008).
19. Kunkle, B.W. *et al.* Genetic meta-analysis of diagnosed Alzheimer's disease identifies new risk loci and implicates Abeta, tau, immunity and lipid processing. *Nat Genet* **51**, 414-430 (2019).
20. de Leeuw, C.A., Mooij, J.M., Heskes, T. & Posthuma, D. MAGMA: generalized gene-set analysis of GWAS data. *PLoS Comput Biol* **11**, e1004219 (2015).
21. Watanabe, K., Taskesen, E., van Bochoven, A. & Posthuma, D. Functional mapping and annotation of genetic associations with FUMA. *Nat Commun* **8**, 1826 (2017).
