# Supplementary material for: Rare SLC13A1 variants associate with intervertebral disc disorder highlighting role of sulfate in disc pathology
Source: Nat Commun. 2022 Feb 2;13:634. doi: 10.1038/s41467-022-28167-1 (PMC8810832; doi:10.1038/s41467-022-28167-1)
Supplement: Supplementary file 2 — Description of Additional Supplementary Files [file 41467_2022_28167_MOESM2_ESM.pdf]

Description of Additional Supplementary Files

File name: Supplementary Data 1
Description: Cohorts included in the back pain GWAS meta-analyses of Dorsalgia, Intervertebral disc disorder (IDD) and lumbar IDD requiring surgery (surgical removal of a herniated lumbar disc (LDHsurg). Number of sequence variants analyzed and number of cases and controls in each of the GWAS cohorts meta-analyzed.

File name: Supplementary Data 2
Description: Genetic correlations between intervertebral disc disorders (IDD), dorsalgia and commonly reported risk factors for back pain; Osteoarthritis (OA), Body Mass Index (BMI) Height, Weight, Bone mineral density (BMD) of the lumbar spine DXA (Dual energy X-ray Absorptiometry) area L1234 (lumbar spine 1 through 4), Major depressive disorder, and Stress.

File name: Supplementary Data 3
Description: List of independent sequence variants associated with back pain defined by IDD (ICD10-M51) in genome-wide meta-analysis on study populations from Iceland, UK Biobank, Denmark, and Finland. Association results are shown per GWAS cohort and combined in GWAS meta-analyses. Also shown are dorsalgia association results for the IDD variants.

File name: Supplementary Data 4
Description: List of independent sequence variants associated with back pain defined by dorsalgia (ICD10-M54) in genome-wide meta-analysis on study populations from Iceland, UK Biobank, Denmark, and Finland. Association results are shown per GWAS cohort and combined in GWAS meta-analyses. Also shown are IDD association results for the dorsalgia variants.

File name: Supplementary Data 5
Description: Coding variants among IDD variants and/or correlated ($r^2 \geq 0.8$) with IDD variants as defined by variant effect predictor
https://m.ensembl.org/info/docs/tools/vep/script/vep_cache.html

File name: Supplementary Data 6
Description: Coding variants among dorsalgia variants and/or correlated ($r^2 \geq 0.8$) with dorsalgia variants as defined by variant effect predictor
https://m.ensembl.org/info/docs/tools/vep/script/vep_cache.html

File name: Supplementary Data 7
Description: Expression quantitative trait loci (eQTL) effects in standard deviations (SD) for IDD variants identified in this study and correlated ($r^2 \geq 0.8$) variants.

File name: Supplementary Data 8
Description: Expression quantitative trait loci (eQTL) effects in standard deviations (SD) for dorsalgia variants identified in this study and correlated ($r^2 \geq 0.8$) variants.

File name: Supplementary Data 9
Description: Loci where the lead variants for IDD (shown in Supplementary Data 3) or highly correlated ($r^2 \geq 0.8$) variants associate with plasma protein levels (pQTL) (SomaScan, Methods)

File name: Supplementary Data 10
Description: Loci where the lead variants for dorsalgia (shown in Supplementary Data 4) or highly correlated ($r^2 \geq 0.8$) variants associate with plasma protein levels (pQTL) (SomaScan, Methods)

File name: Supplementary Data 11
Description: Test of markers associating with self-reported back pain in the GWAS meta-analysis of Freidin et al., 2019, in these current meta-analyses of dorsalgia and intervertebral disc disorders (IDD). Here, results are presented in GWAS-meta-analyses using only the non-UK data to prevent sample overlaps (excluding UK Biobank data, see Supplementary Data 1).

File name: Supplementary Data 12
Description: GWAS meta-analysis results for the surgically defined lumbar disc disease (surgical removal of herniated lumbar discs, LDHsurg) phenotype as defined in Bjornsdottir et al. (2017). Here we meta-analyze GWAS results from the three cohorts where this surgical phenotypes was available; from Iceland, UKBiobank and Denmark with Total Ncases = 9,188, Nctrls = 780,323

File name: Supplementary Data 13
Description: *SLC13A1* loss of function (LOF) variants identified in the three datasets for which individual level genotypes were available (Iceland, Denmark, UKBiobank) and their associations with intervertebral disc disorders (IDD) in the respective datasets. Also shown are all *SLC13A1* markers that were not tested individually, but tested combined in the *SLC13A1* burden test.

File name: Supplementary Data 14
Description: Identified high impact variants in *SLC13A1* and *SLC26A1* and their associations with IDD and serum sulfate measures in Icelanders and published serum sulfate GWAS by Tise et al. (2016).

File name: Supplementary Data 15
Description: *SLC13A1* loss of function variant (p.Arg12Ter) association with selected phenotypes in Icelandic and UKB data, phenotypes selected based on association reports from human and animal studies cited in main text.

File name: Supplementary Data 16.
Description: Lead IDD markers and correlated ($r^2 \geq 0.8$) markers and their previously reported associations listed in the GWAS catalogue.

File name: Supplementary Data 17.
Description: Lead dorsalgia markers and correlated ($r^2 \geq 0.8$) markers and their previously reported associations listed in the GWAS catalogue.
