# Supplementary material for: Rare SLC13A1 variants associate with intervertebral disc disorder highlighting role of sulfate in disc pathology
Source: Nat Commun. 2022 Feb 2;13:634. doi: 10.1038/s41467-022-28167-1 (PMC8810832; doi:10.1038/s41467-022-28167-1)
Supplement: Supplementary file 4 — Reporting Summary [file 41467_2022_28167_MOESM4_ESM.pdf]

Corresponding author(s): Gyda Bjornsdottir, Thorgeir E. Thorgeirsson, Kari Stefansson,

Last updated by author(s): Dec 1, 2021

Reporting Summary

Nature Portfolio wishes to improve the reproducibility of the work that we publish. This form provides structure for consistency and transparency in reporting. For further information on Nature Portfolio policies, see our Editorial Policies and the Editorial Policy Checklist.

Statistics

For all statistical analyses, confirm that the following items are present in the figure legend, table legend, main text, or Methods section.

n/a	Confirmed	
☐	☒	The exact sample size (*n*) for each experimental group/condition, given as a discrete number and unit of measurement
☐	☒	A statement on whether measurements were taken from distinct samples or whether the same sample was measured repeatedly
☐	☒	The statistical test(s) used AND whether they are one- or two-sided<br>*Only common tests should be described solely by name; describe more complex techniques in the Methods section.*
☐	☒	A description of all covariates tested
☐	☒	A description of any assumptions or corrections, such as tests of normality and adjustment for multiple comparisons
☐	☒	A full description of the statistical parameters including central tendency (e.g. means) or other basic estimates (e.g. regression coefficient) AND variation (e.g. standard deviation) or associated estimates of uncertainty (e.g. confidence intervals)
☐	☒	For null hypothesis testing, the test statistic (e.g. *F*, *t*, *r*) with confidence intervals, effect sizes, degrees of freedom and *P* value noted<br>*Give P values as exact values whenever suitable.*
☒	☐	For Bayesian analysis, information on the choice of priors and Markov chain Monte Carlo settings
☒	☐	For hierarchical and complex designs, identification of the appropriate level for tests and full reporting of outcomes
☒	☐	Estimates of effect sizes (e.g. Cohen's *d*, Pearson's *r*), indicating how they were calculated

Our web collection on statistics for biologists contains articles on many of the points above.

Software and code

Policy information about availability of computer code

Data collection	Phenotype and genotype data used in this study was from four GWAS studied cohorts. No special software was used for data collection.
Data analysis	GraphTyper (version 2.0-beta) is software that uses pangenome graphs to genotype structural variants and small variants using short reads. Graphtyper is available at https://github.com/DecodeGenetics/graphtyper/<br><br>Eagle2 is a phasing algorithm that attains high accuracy across a broad range of cohort sizes by efficiently leveraging information from large external reference panels (such as the Haplotype Reference Consortium; HRC). Eagle2 is open source and freely available for HRC-based phasing (see ref. 63, Loh et al.). Eagle2 software and source code: http://www.hsph.harvard.edu/alkes-price/software/.<br><br>BOLT-LMM is software used to test linear and mixed-model associations in large samples. BOLT-LMM is open source and available at http://www.hsph.harvard.edu/alkes-price/software/.<br><br>For IVW-MR and MR-Egger we used functions from the stats package in R (version 3.6.3) to fit weighted linear models (see: http://r.meteo.uni.wroc.pl/web/packages/dplR/vignettes/intro-dplR.pdf).<br><br>For visualizations we used the R package ggplot2 (see: https://wires.onlinelibrary.wiley.com/doi/abs/10.1002/wics.147).<br><br>We used the Heidi outlier removal approach, which is included in the Generalised Summary-data-based Mendelian Randomisation (GSMR) method. For GSMR, we used the implementation from the R package v1.0.9 available at https://cnsgenomics.com/software/gsmr/.<br><br>MAGMA is a tool for gene analysis and generalized gene-set analysis of GWAS data and is available at http://ctglab.nl/software/magma

FUMA is a platform that can be used to annotate, prioritize, visualize and interpret GWAS results and is available at https://fuma.ctglab.nl/.

No custom code was written for this study.

For manuscripts utilizing custom algorithms or software that are central to the research but not yet described in published literature, software must be made available to editors and reviewers. We strongly encourage code deposition in a community repository (e.g. GitHub). See the Nature Portfolio guidelines for submitting code & software for further information.

Data

Policy information about availability of data

All manuscripts must include a data availability statement. This statement should provide the following information, where applicable:

- Accession codes, unique identifiers, or web links for publicly available datasets
- A description of any restrictions on data availability
- For clinical datasets or third party data, please ensure that the statement adheres to our policy

The GWAS results from this study are deposited at https://www.decode.com/summarydata/.
GWAS summary statistics from Finland are restricted to researchers representing the consortium partners and were downloaded from https://rf.finngen.fi
Other data generated or analyzed during this study are included in Supplementary Data.

Field-specific reporting

Please select the one below that is the best fit for your research. If you are not sure, read the appropriate sections before making your selection.

☒ Life sciences ☐ Behavioural & social sciences ☐ Ecological, evolutionary & environmental sciences

For a reference copy of the document with all sections, see nature.com/documents/nr-reporting-summary-flat.pdf

Life sciences study design

All studies must disclose on these points even when the disclosure is negative.

Sample size	The sample size was determined by combining with Icelandic data from GWAS's of the back pain phenotypes of interest to the study, GWAS data from three other cohorts where cases with IDD and and dorsalgia were available. We sought data from the largest datasets with comparable data to the Icelandic GWASs and with participants of European descent. In addition to the Icelandic dataset, we analyzed data from the UK Biobank, Denmark and Finnland, resulting in total in 119,110 dorsalgia cases vs 909,847 controls and 58,854 IDD cases vs 922,958 controls. Thus, this is the largest GWAS meta-analysis of these back pain phenotypes to date.
Data exclusions	We excluded sequence variants with imputation information below 0.8 and MAF below 0.01% for quality reasons.
Replication	Our study is a meta-analysis of GWAS studies with no direct replication. However, we studied associations of previously published variants associating in UK data with self-reported back pain (Freidin, M.B. et al. (2019) Insight into the genetic architecture of back pain and its risk factors from a study of 509,000 individuals. Pain 160, 1361-1373). We studied these reported variants in IDD and Dorsalgia meta-analyses of Icelandic, Danish and Finnish data, excluding the UKB dataset to avoid potential data overlap. Furthermore, we meta-analyzed GWASs of the severest IDD cases who required surgical interventions, from the three cohorts where this data was available: Iceland, UK Biobank and Denmark (9,188 cases and 780,323 controls).
Randomization	No randomizations were used in this study as it is based on GWAS meta-analyses.
Blinding	This is an observational association study and no blinding was required.

Reporting for specific materials, systems and methods

We require information from authors about some types of materials, experimental systems and methods used in many studies. Here, indicate whether each material, system or method listed is relevant to your study. If you are not sure if a list item applies to your research, read the appropriate section before selecting a response.

Materials & experimental systems

n/a	Involved in the study
x	Antibodies
x	Eukaryotic cell lines
x	Palaeontology and archaeology
x	Animals and other organisms
	x Human research participants
x	Clinical data
x	Dual use research of concern

Methods

n/a	Involved in the study
x	ChIP-seq
x	Flow cytometry
x	MRI-based neuroimaging

Human research participants

Policy information about studies involving human research participants

Population characteristics	All subjects from the 4 studied GWAS cohorts were adults with IDD or Dorsalgia over 18 years of age of European descent. For the Icelandic and Danish data the study did not have age at first event but both cohorts included cases with population representative sex distributions and large representative control samples, as does the data obtained from Finngen and UKBiobank. Case details for the Finngen cohort are available at https://risteys.finngen.fi/phenocode and for the UK Biobank cohort at https://biobank.ndph.ox.ac.uk/showcase/index.cgi.
Recruitment	All cohorts recruit from the general population (deCODe Genetics in Iceland) and/or large volunteer research organizations (CHB-DBDS in Denmark, UKBiobank in the United Kingdom and Finngen in Finland).<br><br>In Iceland, a large fraction of adults (18 years and older) in the population of 360,000 inhabitants, has participated in a nationwide research program at deCODE Genetics. Participants in this study on back pain phenotypes were selected based on diagnostic data from the Registry of Primary Health Care Contacts, Registry of Hospital Diagnoses, and Registry of Contacts with Medical Specialists in Private Practice, which cover the entire population of Iceland.<br><br>The UK Biobank study is a large cohort (just over 500,000 participants) in the age range of 40-69 year old at recruitment. Participants in this study were identified from data gathered by the study from The UK National Health System's General Practice Records and Hospital Diagnostic Data.<br><br>The Danish participants are from the Danish Blood Donor Study (DBDS) GWAS study, which is a large prospective cohort study of ~110,000 blood donors across Denmark, and from the Copenhagen Hospital Biobank (CHB). which is a research bank, collecting left-over samples obtained from diagnostic procedures on hospitalized and outpatient patients in the Danish Capital Region hospitals.<br><br>Finnish data were obtained from the ongoing FinnGen study, that collects samples and phenotype data from a nationwide network of Finnish biobanks and digital health care data from national health registries. Currently, the Finngen study includes 356,000 participants with both genotype and health registry data.<br><br>Using this approach, it is unlikely that recruitment methods within the cohorts selected for the GWAS meta-analyses, entail selection bias or have other impact on our results.
Ethics oversight	All cohorts collected data and samples under local and/or institutional approved ethics committee reviews.<br><br>Icelandic data for this study were analyzed under National Bioethics Committee (NBC) Licenses #VSN-17-035 and #VSN-12-162 (with amendments), issued following review by the Icelandic Data Protection Authority (DPA).<br><br>The UKBiobank's scientific protocol was reviewed and approved by The North West Research Ethics Committee (REC Reference Number: 06/MRE08/65). Data for this study were obtained and research conducted under the UKB application license number 24898.<br><br>The Danish Data Protection Agency (P-2019-99) and the Danish National Committee on Health Research Ethics (NVK-1700704) approved the studies under which genetic data on DBDS participants were obtained. The DBDS data requested for this study was approved by the DBDS steering committee. Danish samples from the Copenhagen Hospital Biobank (CHB) were also included as part of the study on pain-related diseases under the genetics of pain and degenerative musculoskeletal disease protocol (NVK-1803012).<br><br>The Coordinating Ethics Committee of the Helsinki and Uusimaa Hospital District evaluated and approved the FinnGen research project. The project complies with existing legislation (in particular the Biobank Law and the Personal Data Act). The official data controller of the study is University of Helsinki.

Note that full information on the approval of the study protocol must also be provided in the manuscript.
